# Pan‐Arctic Peatlands Have Expanded During Recent Warming

**DOI:** 10.1111/gcb.70684

**Published:** 2026-02-13

**Authors:** J. Handley, R. E. Fewster, T. G. Sim, S. Hodson, B. Parker, K. Crichton, D. Charman, K. Anderson, M. Garneau, M. Väliranta, D. W. Beilman, G. T. Swindles, M. Aquino‐López, M. Blaauw, X. Comas, E. Levesque, V. Maire, H. Addis, M. Amesbury, D. Fortier, M. Mleczko, A. Gallego‐Sala

**Affiliations:** ^1^ Department of Geography University of Exeter Exeter UK; ^2^ Geography and ^14^Chrono, School of Natural and Built Environment Queen's University Belfast Belfast UK; ^3^ Geography The University of Manchester Manchester UK; ^4^ Forest Research, Northern Research Station Midlothian UK; ^5^ Environment and Sustainability Institute, Centre for Geography and Environmental Sciences University of Exeter Penryn UK; ^6^ Department of Geography and Geotop Research Center‐Université du Québec à Montréal Montréal Canada; ^7^ Environmental Change Research Unit (ECRU), Ecosystems, Environment Research Programme University of Helsinki Helsinki Finland; ^8^ Department of Geography and Environment University of Hawaiʻi at Mānoa Honolulu USA; ^9^ Ottawa Carleton Geoscience Centre and Department of Earth Sciences Carleton University Ottawa Ontario Canada; ^10^ Centre for Climate Repair, Department of Geography University of Cambridge Cambridge UK; ^11^ Department of Geosciences Florida Atlantic University Boca Raton Florida USA; ^12^ Department of Earth and Environment Florida International University Boca Raton Florida USA; ^13^ Département Des Sciences de L'environnement Université du Québec à Trois‐Rivières Trois‐Rivières Quebec Canada; ^14^ Centre for Northern Studies (CEN) Quebec City Quebec Canada; ^15^ School of Geography University of Leeds Leeds UK; ^16^ Département de Géographie Université de Montréal Montreal Quebec Canada

**Keywords:** Arctic peatlands, carbon, environmental change, lateral expansion, peatland extent

## Abstract

The fate of carbon stored in Arctic peatlands remains uncertain because of the complex nature of the effects of climate change on permafrost and peatland carbon cycling. Expansion and/or shrinkage of Arctic peatlands under climate change also remain unknown due to lack of ground data and difficulties detecting changes in the extent of these ecosystems, meaning that land surface model predictions currently inadequately quantify Arctic terrestrial carbon storage changes. Pan‐Arctic shifts in peatland extent would profoundly change the fate of carbon in the terrestrial Arctic. Here, we tackle this knowledge gap by answering three main questions: (a) has lateral expansion occurred in Arctic peatlands as a response to recent warming? (b) if so, how fast has this occurred? (c) how does the response vary regionally? To answer these, we collected a dataset (12 peatland sites, 91 peat cores) combining peat cores collected across two latitudinal (north–south) transects: one in the European Arctic and one in the Canadian Arctic. In each region, we selected three peatland sites, with cores collected from transects spanning the peat‐edge to the peat‐centre. Our large‐scale dataset shows that peatlands have expanded, often rapidly, with some rates exceeding ~1 m per year since 1950 AD. This rapid expansion has occurred during a period of widespread Arctic warming and is still ongoing: two thirds (8/12) of our peatland sites evidence new peat formation after ~1990 AD based on age‐depth models constrained by ^14^C and ^210^Pb dating. Given that our sites comprise a broad range of Arctic conditions, we expect peatland expansion to be a pan‐Arctic phenomenon. Within specific regions, there are constraints on peat expansion including topographical limits, but we present the basis for future work to estimate pan‐Arctic peatland expansion, plus the associated carbon cycle implications under future climate change.

## Graphical abstract

Arctic peatland carbon is important because it represents a large carbon stock that may be vulnerable to climate change. This work explores the expansion and/or shrinkage of Arctic peatlands using observations in two north–south transects in Canada and in Europe. We find that lateral expansion has occurred in Arctic peatlands as a response to recent warming, often rapidly, with some rates exceeding ~1 m per year since 1950 AD. Given the differing conditions of our selected sites, we expect peatland expansion to be a pan‐Arctic phenomenon.

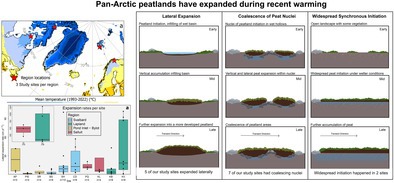

## 1 Introduction

Peatlands in the perennially frozen (permafrost) region of the pan‐Arctic represent a vast, but fragile, terrestrial carbon store (~185 ± 66 Gt C), covering an estimated 1.7 ± 0.5 million km^2^ (Hugelius et al. 2020). Peat accumulates wherever plant litter production exceeds the rate of decomposition for a sustained period (Ruppel et al. 2013). This positive mass balance primarily requires an environment that inhibits plant litter decay (Clymo 1984), but may also occur where plant productivity and litter production substantially increase. The cold climate of the Arctic and the varying prevalence of permafrost has enabled plant litter to slowly accumulate as peat over several millennia (Gorham et al. 2007; Hugelius et al. 2020). This is largely because low‐lying, Arctic landscapes are frequently waterlogged due to low rates of evapotranspiration and exhibit shallow active layers, below which microbial decomposition is suppressed by cold temperatures and minimal water availability (Swindles et al. 2015). Arctic temperatures have increased by almost four times the global average since 1979 (Rantanen et al. 2022), resulting in observations of rapid and widespread environmental changes, including permafrost peatland thaw (Swindles et al. 2015; Olefeldt et al. 2021; Olvmo et al. 2020) and ecological shifts linked to enhanced plant productivity (e.g., Crichton et al. 2022). Increased plant growth and moisture availability under recent and projected temperature and precipitation increases have the potential to generate new loci for peat formation and/or enhance existing peat accumulation (Cleary et al. 2024; Juselius‐Rajamäki et al. 2025, 2023); similar climatic changes in high elevation regions have led to new peats initiating as recently as 50–70 years ago (Fewster et al. 2025). The future trajectory of Arctic peatlands remains highly uncertain because of complex ecohydrological responses to rising temperatures, which have deepened active layers (Strand et al. 2021) and accelerated aerobic decomposition and CO_2_ emissions (Treat et al. 2014) and permafrost thaw, driving widespread surface collapse and inundation, resulting in heightened CH_4_ release (Hodgkins et al. 2014). These factors may promote surface drying and peatland shrinkage in some areas, for example, as a result of enhanced evaportranspiration and modified drainage patterns, whilst others may experience inundation and expand (Kreplin et al. 2021). This highlights the complex feedbacks between climate warming and Arctic peatland dynamics and can have differing scales of implications for the current and future carbon balance of the Arctic.

Currently, the pan‐Arctic permafrost region represents a weak but seemingly increasing CO_2_ sink, with wetlands representing minor CH_4_ sources (Hugelius et al. 2024; Treat et al. 2024; Virkkala et al. 2025). Hypothetical near‐future increases in the spatial coverage of Arctic peatlands could represent a northwards shift of the peatland biome and increase high‐latitude C sequestration. However, the dynamics of recent peatland expansion at latitudinal extremes remain poorly constrained because of limited availability of long‐term palaeoecological data.

Patterns of vertical peat accumulation in northern peatlands have been studied for several decades (e.g., Ovenden 1990; Beilman et al. 2009; Yu 2012; Charman et al. 2013; Loisel et al. 2014; Swindles et al. 2025), but comparatively less attention has been given to the outwards expansion of peatland margins (hereafter, termed lateral expansion). Variation in vertical peat accumulation has been linked to site‐specific, autogenic factors, for example fen‐bog ecological transitions (Liu et al. 2023; Loisel and Yu 2013), and regional‐scale, allogenic controls, most notably declining rates during cool, late‐Holocene climates (Beilman et al. 2009; Charman et al. 2013; Mauquoy et al. 2002).

Climate may exhibit a more complicated control on regional patterns of lateral expansion than vertical accumulation, because marginal peat growth is strongly constrained by local topography (Peng et al. 2024). Adjacent, low‐lying, waterlogged landscapes with a sufficient rate of plant productivity are prerequisites for any lateral expansion to occur (Ehnvall et al. 2023; Ruppel et al. 2013). Furthermore, Arctic peat complexes are often dynamic, heterogeneous ecosystems that have been experiencing recent permafrost thaw (e.g., Germain Chartrand et al. 2023; Olefeldt et al. 2021), leading to shifts in peat‐forming vegetation (e.g., Fewster, Morris, Swindles, Baird, et al. 2023; Piilo et al. 2023), hydraulic properties (Fewster, Morris, Swindles, Ivanovic, et al. 2023) and surface wetness (Taylor et al. 2019; Sim et al. 2019, 2021; Zhang et al. 2022). These dynamic processes have the potential to drive more complicated patterns of peatland expansion in the Arctic than elsewhere, but lateral expansion dynamics of Arctic peatlands remain understudied outside of Fennoscandia (Juselius‐Rajamäki et al. 2023; Mäkilä and Moisanen 2007; Piilo et al. 2020; Weckström et al. 2010). Uncertainty regarding the timing, nature and extent of any recent lateral expansion of Arctic peatlands presents a critical research gap that our study aims to address. One well established method to determine the spatiotemporal history of lateral peatland expansion is to use numerous, well‐dated cores, often positioned along linear transects from the peatland margin towards its centre (e.g., Korhola 1995, 1994; Juselius‐Rajamäki et al. 2023, 2025). Given the difficulties in extracting, transporting, and dating multiple cores from a single site, minimal data exist to derive rates of recent lateral expansion for Arctic peatlands. Several previous studies have considered this phenomenon in subarctic Fennoscandia, recording lateral expansion rates of up to 80 cm yr.^−1^ (Juselius‐Rajamäki et al. 2023), with the greatest expansion occurring during the wetter early Holocene (~10,000–~7000 cal. yr. BP) and Neoglacial (since ~4000 cal. yr. BP; Mäkilä and Moisanen 2007; Mathijssen et al. 2014; Piilo et al. 2020; Weckström et al. 2010). Recently Juselius‐Rajamäki et al. 2023, showed that at Syysjärvi, Finnish Lapland, peatlands are still expanding, with late‐20th century basal dates recorded at the peat margins, while the same authors identified non‐linear dynamics of expansion in another subarctic peatland in Finland (Juselius‐Rajamäki et al. 2025). This evidence suggests that post‐industrial climate warming and wetting may have recently renewed expansion of existing Finnish‐Arctic peatlands and increased the availability of peat‐forming locations at northern high‐latitudes. However, core‐based information on past lateral expansion is unavailable for much of the pan‐Arctic, including the Canadian Arctic Archipelago and Svalbard, meaning the regional‐scale areal response of Arctic peatlands to changing climate remains unknown.

The latest Earth system models (e.g., Eyring et al. 2016) recently projected that substantially higher warming may occur during the 21st century than previously suggested (Sobie et al. 2021; Tokarska et al. 2020), which could accelerate high‐latitude ecosystem changes with consequences for the net carbon balance of the Arctic. There is a pressing need, therefore, to establish how Arctic peatlands respond to climate warming, but no study has investigated long‐term observational evidence for recent peatland expansion at a pan‐Arctic scale. The current absence of such empirical data for Arctic peatlands limits modelling of the developmental trajectories of these dynamic carbon sinks.

In this study, we employ a palaeoecological framework to investigate evidence for recent expansion of Arctic peatlands by addressing the following research questions:

- Has lateral expansion occurred in Arctic peatlands as a response to recent warming?
- If this is the case, how fast have Arctic peatlands expanded, and how do these rates vary between regions and across latitudinal gradients?
- What are the topographic control and mechanisms of expansion within the peatlands?

## 2 Materials and Methods

### 2.1 Study Sites

**FIGURE 1:**
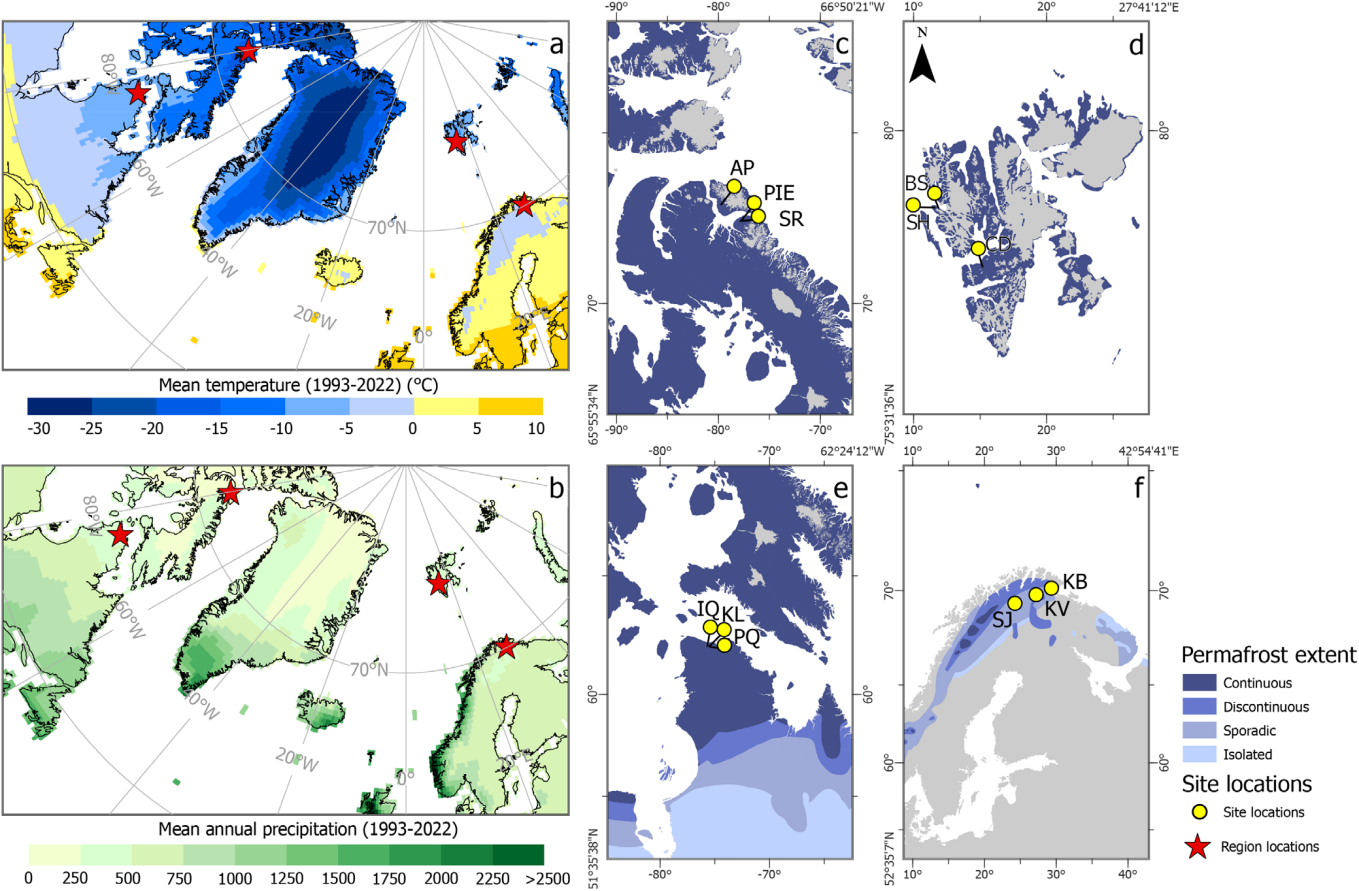
Location of study sites and regional site locations shown with regional climate and permafrost distributions. Mean annual temperature (^o^C) and mean annual precipitation (mm) for the period 1993–2022, (30‐year average covering our last coring date: 2022) are shown in the two left panels. Continuous, Discontinuous, Sporadic, and Isolated Permafrost Zones are shown as different shades of blue in right hand panels. The sites are indicated by letter codes (from top left to bottom right); AP (Angela's Paradise); PIE (Pond Inlet East); SR (Salmon River); BS (Blomstrand); SH (Stuphallet); CD (Colesdalen); IQ (Iqatutuut Valley); KL (Kissuujaaluk Low); PQ (Peat Qilaliariak); SJ (Suossjavri); KV (Kevo); KB (Karlebotn).

**FIGURE 2:**
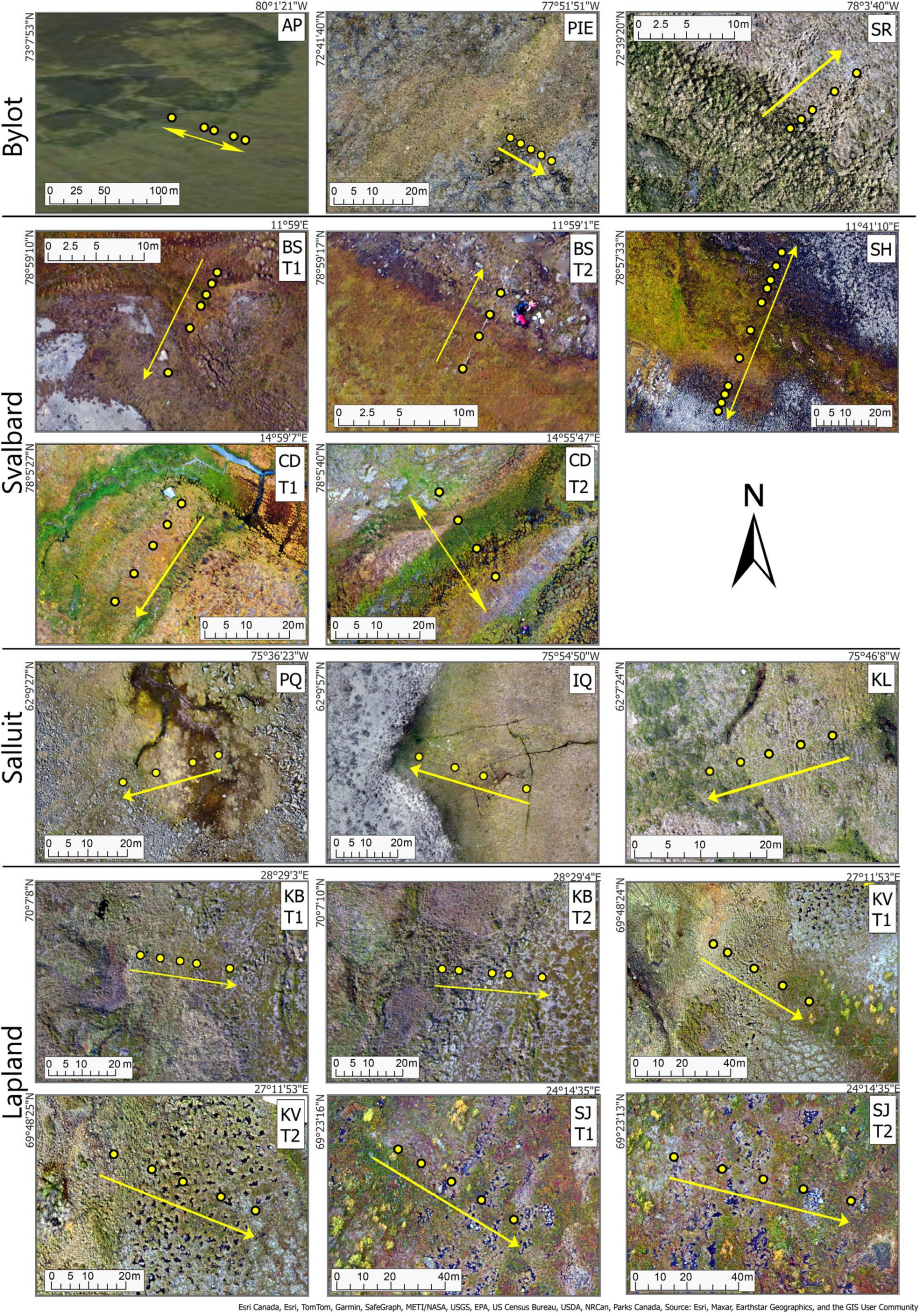
Coring locations along each site transect, shown on site‐level drone aerial photographs. Site naming follows that in Figure 1, with the number of the transect added (e.g., BS T1 = Blomstrand Transect 1). Arrows point in the direction of the edge of the peatland. Where a double‐ended arrow is shown, peatlands were sampled from edge to edge across the peatland as opposed to centre to edge for the single directional arrows. Satellite imagery for Angela's Paradise obtained from ArcGIS World Imagery due to drone use not being permitted at this site.

A total of 12 representative sites were selected for studying peatland expansion across the Arctic (Figures 1 and 2). We focused our analysis on a total of four regions in the Arctic to cover the spatial variability of the Arctic and covering two North‐to‐South transects: one in Europe (Svalbard, Lapland) and one in Canada (Bylot, Salluit). The more northerly regions (Bylot, Svalbard) are situated within the continuous permafrost zone, where peatland development is expected to be sporadic; however, more recent temperature change may have begun to drive vegetation changes, encouraging the early stages of peatland spread. The more southerly region of Lapland is already known to have extensive peatland coverage within the sporadic and discontinuous permafrost zone. Salluit represents the southern extent of the Canadian North‐to‐South transect and experiences a warmer annual temperature range than that in Bylot, despite being within the continuous permafrost zone (Figure 1, Table 1). Using these two latitudinal transects permits us to test the effect of differing climatic conditions and latitudinal gradient on the rates of peatland lateral extension in relation to recent warming. At present, mean annual temperatures across the sites range from −13.5°C in Bylot Island in the High Arctic region to +0.1°C in Lapland within the Low Arctic; annual mean precipitation ranges from 208.4 mm yr.^−1^ (Svalbard, High Arctic) to 602.8 mm yr.^−1^ (Salluit, Low Arctic) for the period of 1985–2020 C.E., based on nearest weather station data (Table 1; see also Crichton et al. 2022, Supporting Information, references therein).

**TABLE 1:** Overview of study sites, including local climatic conditions, dominant peat vegetation and underlying sedimentology.

Region	Permafrost zone	Sub‐ region	Site	Latitude	Longitude	Nearest weather station	Mean annual temperature (°C)	Dominant vegetation	Underlying sedimentology	Peatland type
High‐Arctic	Continuous	Bylot Island	Angela's Paradise	73.13028	−80.022987	Bylot Lac aux Goélands (73.1505, −80.004), Bylot Lac du camp (73.156, −79.957), Bylot SILA (73.152, −79.989 ; Centre d'études nordiques (CEN) 2024)	−13.5	Herbaceous plants (e.g., *Eriophorum* spp.) and *Sphagnum* mosses	Clay	Low‐centred polygon mire
		Pond Inlet	Salmon River	72.655515	−78.061646	Pond Inlet Airport (72.69, −77.97)	−13.3	Brown mosses, sedges (e.g., *Carex aquatilis* , *Eriophorum angustifolium* ), grasses and some dwarf shrubs (e.g., *Salix reticulata* )	Sandy clay	Stream associated/valley mire
			Pond Inlet East	72.694207	−77.864603			Mosses (e.g., *Sphagnum* spp. and *Aulacomnium* spp.), sedges (e.g., *Carex* spp. and *Eriophorum arcticum*) and grasses, some dwarf shrubs (e.g., *Salix reticulata* )	Rocks and gravel	Stream associated incipient peatland
		Svalbard	Blomstrand	78.98595	11.98246	Ny‐Ålesund (78.923, 11.932)	−3.2	Mosses with some grasses and a few vascular plants (e.g., *Saxifraga* spp. and *Salix* spp.)	Rocks and gravel	High‐Arctic peatland
			Colesdalen	78.09065	14.98407	Svalbard Airport (78.246, 15.493)	−4.6	Mosses, with few vascular plants (e.g., *Salix* spp.), and few grasses	Silt	Overgrown polygon mire
			Stuphallet	78.95872	11.68331	Ny‐Ålesund (78.923, 11.932)	−3.2	Mosses, some vascular plants (e.g., *Anagallis* spp.), and few grasses	Rocks and gravel	High‐Arctic incipient peatland
Low‐Arctic	Continuous	Salluit	Iqalutuut Valley	62.165681	−75.915017	Salluit SILA (62.192, −75.636)	−6.26	Mainly mosses (e.g., *Polytrichum* spp. and *Sphagnum squarrosum* ) some sedges ( *Carex aquatilis* ), and grasses	Sand	Low‐Arctic incipient peatland
			Kissuujaaluk Low	62.123168	−75.769467			Mainly mosses (e.g., *Sphagnum* spp., *Polytrichum* spp., and *Aulacomnium* spp.), some sedges (e.g., *Carex aquatilis* ), and grasses (e.g., *Eragrostis* spp.)	Sand, clay and rocks	Overgrown polygon mire
			Peat Qilaliariak	62.157355	−75.607301			Mainly mosses (e.g., *Polytrichum* spp. and *Scorpidium* spp.), some sedges (e.g., *Carex aquatilis* ), and grasses	Clay and rocks	Stream associated/valley mire
	Discontinuous/Sporadic	Lapland	Karlebotn	70.11876	28.48351	Rustefjelbma (70.39885, 28.19316)	0.1	Small shrubs (e.g., *Betula nana* ), lichens, mosses (e.g., *Sphagnum* spp, and some sedges and grasses)	Sand and gravel	Degraded palsa mire
	Discontinuous		Kevo	69.80659	27.19732	Utsjoki Kevo (69.75, 27.01667)	−1.3	Small shrubs, lichens, mosses (e.g., *Sphagnum* spp. *Polytrichum* spp. and *Dicranum* spp.), and some grasses	Sand and rocks	Degraded palsa mire
			Suossjavri	69.38787	24.24123	Cuovddatmohkki (69.3667, 24.4333)	−1.5	Mosses (e.g., *Sphagnum* spp., *Polytrichum* spp. And *Dicranum* spp.) and grasses, few woody plants and shrubs (e.g., *Betula* spp. and *Salix* spp.)	Sand and rocks	Subarctic peatland

### 2.2 Field Sampling

The selection of sites in the High‐Arctic and Low‐Arctic in two regions, Canada and Europe, allows for the comparison of two North‐to‐South transects. Fieldwork was conducted during July–August 2019 (Svalbard and Lapland) and July–August 2022 (Bylot Island, Pond Inlet, and Salluit), where the focus was on recently developed peatlands. Prior to fieldwork, possible locations for nascent peatlands were identified from local expert knowledge and observations supported by satellite data where recent changes (between 1985 and 2020) in a greenness proxy (NDVI) and moisture proxy (NDMI) were analysed. At each site, the surface vegetation, water table depth, and active‐layer thickness (where possible) were also recorded.

**FIGURE 3:**
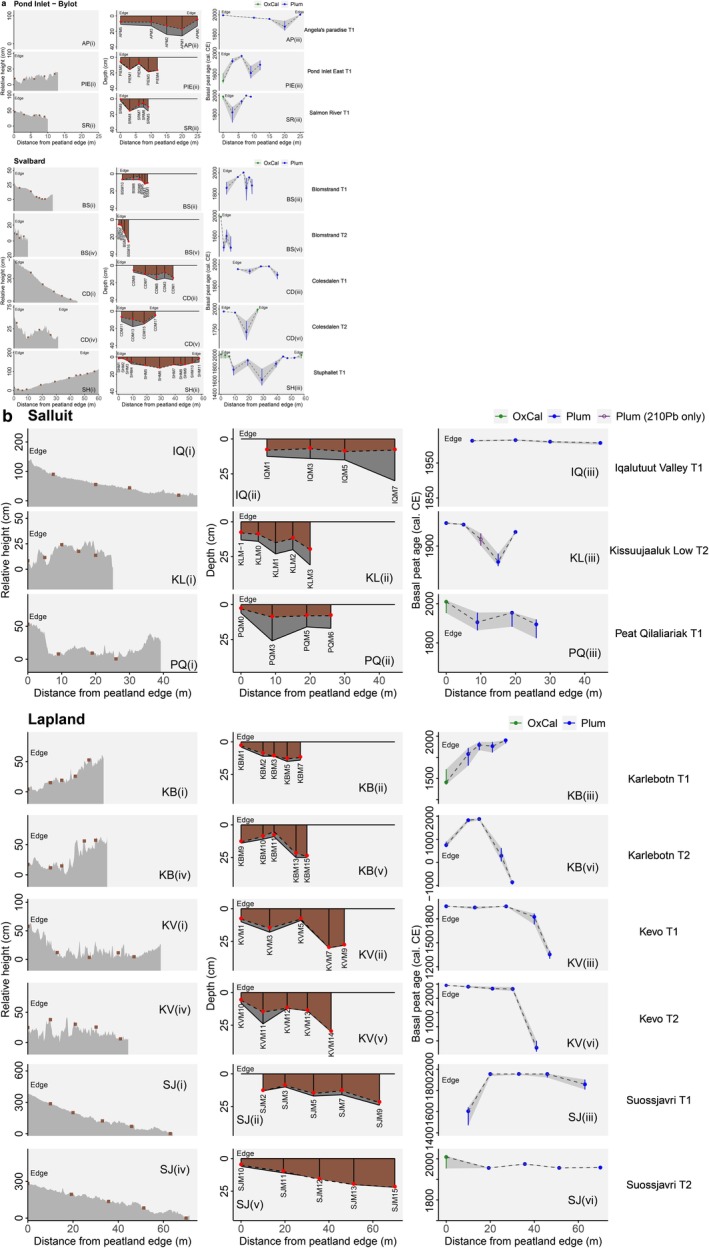
(a) Evidence used to establish lateral expansion for peatlands in the High Arctic. Surface topography presented as relative surface height (cm), except for Angela's paradise where no drone data was available, peat depth of cores along the transect (red dots represent depth of ^14^C date for each core), and basal peat age (cal. C.E) for transects in the High Arctic. Sites have been plotted according to temperature from coldest (Angela's Paradise) to warmest (Stuphallet), see Table 1. (b) Evidence used to establish lateral expansion for peatlands in the Low Arctic. Surface topography presented as relative surface height (cm), peat depth of cores along the transect (red dots represent depth of ^14^C date for each core), and basal peat age (cal. C.E) for transect in the Low Arctic. Sites have been plotted according to temperature from coldest (Iqalutuut) to warmest (Suossjavri), see Table 1.

A lightweight consumer drone (DJI Mavic Pro 2) equipped with a 3‐axis gimbal stabilised optical (red, green, blue) camera was used to capture centimetric spatial resolution aerial photography at all but one site so that the surface expression of ecosystem properties on the peatland surface could be understood; we could not obtain the necessary permissions to implement drone surveys at the site named “Angela's Paradise” on Bylot Island (73.13028, −80.022987). Ground targets were deployed to constrain the subsequent photogrammetric processing of overlapping images (using Agisoft Metashape Professional version 1.5). This workflow generated two products: a generalised ortho‐mosaic showing spatial ecology, and a point cloud quantifying the surface morphology. QGIS (version 3.28.11) was used for spatial product analysis including the generation of a surface topography anomaly product (expressed relative to the minimum elevation along the transect) at fine spatial resolution (2 cm per pixel) over each of the sites. The networks of 11 ground control targets deployed and surveyed with handheld GPS at each site were used to check on the accuracy of the photogrammetric reconstruction, acknowledging that these would themselves contain some spatial inaccuracies due to acknowledged limitations of hand‐held GPS records (+/− ~5 m accuracy). During post‐processing, the ground control targets were used as guides to correctly locate the transect on the drone‐captured topography data in order to retrieve peatland surface heights. These data were downscaled to 0.1 m resolution to allow for direct comparisons with core sampling locations, as shown in Figure 3a,b.

From each of the 12 peatlands, a total of 91 monolith cores were recovered from 14 centre‐edge and three edge‐centre‐edge transects (Figure 2) in order to document lateral expansion rates. Peatland margins were determined from an absence of peat during exploratory sampling or by field observations of a distinct surface vegetation change to non‐peatland communities. Transects were positioned in areas that evidenced recent peat formation, for example, as indicated by early vegetation succession, and which demonstrated gradients from shallow, marginal peats to deeper, central peats. It was not possible in the field to know for certain whether older parts of the sequence were in the centre, as peat could have formed from different nuclei and coalesced over time. Instead, this methodology focused on retrieving the shallower sediments that potentially represented recent expansion at the edges of peatlands.

Unfrozen active‐layer peat samples were collected by inserting a monolith tin into the peatland as deep as possible until basal sediments or permafrost were reached. Surrounding material was cut away with a long, serrated knife before extracting each monolith. For some deeper, permafrost peats, such as those from Angela's Paradise, frozen samples up to 1 m in depth were collected using a permafrost corer. Cores were wrapped tightly with cling film for transport off site in polyvinyl chloride (PVC) pipes or in Correx boxes sealed in plastic bags. Permafrost cores were kept frozen during transit. All cores were transported back to the University of Exeter laboratories; unfrozen cores were stored in a cold store (~4°C) and frozen cores were kept frozen (−26°C).

### 2.3 Measurements of Peat Properties

The 91 monoliths were subsampled at 1 cm resolution for the analysis of bulk density, moisture content, and C/N analysis to aid in the determination of the total peat depth of each of the monolith cores (see below and Dataset S1). For the frozen permafrost samples, cores were cut into 1 cm slices using a fine‐toothed hacksaw after being left to thaw for 30 min. Dry bulk density (g cm^−3^), organic matter content (via Loss on Ignition), and moisture content (%) analysis of 4 cm^3^ samples from each 1 cm slice followed an adapted version of Chambers et al. (2010). Samples were placed in a freeze‐drier for 48 h and sample weights were recorded before and after drying. For C/N analysis, samples were weighed into tin capsules using a microbalance. Dried peat samples were weighed to within 0.5 mg of 5 mg. EDTA standards were used to calibrate the C/N analysis, these were weighed to within 0.2 mg of 3 mg. Prepared samples were analysed using an Organic Element Analyser (Flash 2000) to determine %C and %N.

The contact between organic and mineral sediment was determined using a combination of carbon content (%) and bulk density. There is no universally accepted definition for the mineral‐peat boundary (Lourenco et al. 2022; Quik et al. 2022) so we studied existing pan‐Arctic data syntheses of peat properties (Zoltai et al. 2000; Treat et al. 2016). In our study, we define peat to have a C content of ≥ 25% and a dry bulk density of ≤ 0.25 g cm^−3^. We used these values to determine the deepest depth in each core where peat exists and dated these layers to derive basal ages. Where a clear transition across these thresholds was not available, we primarily determined peat depths to be the deepest point where C content was > 25%. For some of the incipient peatlands in Iqalutuut (Salluit), carbon content was generally quite low (< 25%), which made it harder to define peat depth using this threshold. This was primarily as a result of the high sand content in the profiles, deposited on the peatland from the surrounding sand dunes. However, the mossy peat grew on top of sand, which made for a sharp transition from organic to mineral sediments. Loss on ignition (LOI) was additionally carried out on larger subsamples to check whether this method could better capture the organic portion of the peat. After the initial ashing in the furnace at 550°C, some organic matter still remained; consequently, the samples were returned to the furnace at 925°C for 1 h to combust any remaining organic matter. LOI still yielded relatively low organic matter content. For these sequences, visible changes in the cores in combination with the LOI, C% and BD were used to determine peat depth.

### 2.4 Core Chronologies

A combination of ^14^C and ^210^Pb dating techniques was used to construct age‐depth models for each of the cores. Basal peats (as identified above) were dated by ^14^C analysis using primarily plant macrofossils; however, where no suitable macrofossils were present, bulk peat samples were prepared (26/90 samples). Any surface material, or material exposed on the outside of the core, was removed in case of contact with modern material and contamination. Samples were sieved through a 250‐μm sieve. Material > 250 μm was transferred to a petri dish and dispersed in deionised water. The samples were analysed under a 10×–60× magnification stereo microscope and visually assessed for suitable above‐ground material for dating. Picked plant macrofossils were transferred to a glass vial with deionised water and a couple of drops of HCl. These were sent to the University of Ottawa, Canada (Svalbard samples) or Queen's University, Belfast (Lapland and Canada samples) for radiocarbon dating via accelerated mass spectrometry (AMS).

^210^Pb dating was conducted on near‐surface sections (< 30 cm) until background or the end of the core was reached, for each of the 91 monoliths. Sample preparation for analysis via alpha spectrometry was carried out following methods outlined in Sanderson (2016). Samples were analyzed within the Department of Geography at the University of Geography. Samples were left in the detectors until a minimum of 400 ^210^Po counts were reached. Where this was not achievable, samples were left for a maximum of 2 weeks.

**FIGURE 4:**
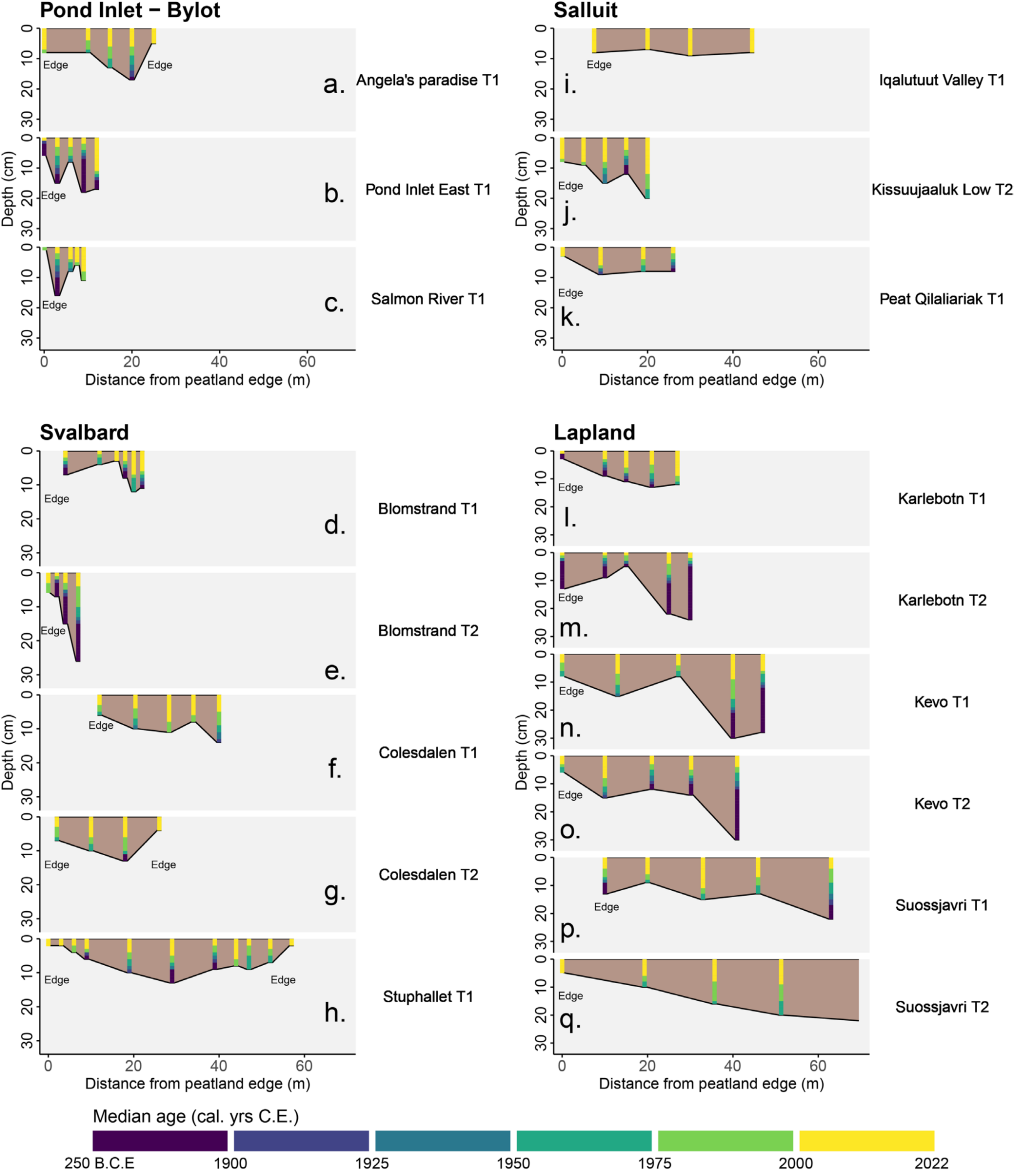
Modelled peat ages with depth for each monolith and transect from our studied Arctic peatlands. Accumulation prior to 1900 is illustrated by the purple bar, with the oldest basal peat age being 250 cal B.C.E. For the Bayesian age depth models for each core see Figure S1.

Bayesian age depth models were fitted for each monolith core using the ^14^C dates and ^210^Pb activity in the rPlum package v0.3.0 (Aquino‐López et al. 2018) for 78/91 cores. Each ^14^C date was calibrated using the IntCal20 (Reimer et al. 2020) and NH1 post‐bomb (Hua et al. 2021) radiocarbon calibration curves. For those cores with a peat depth of ≤ 6 cm (12/91), a Bayesian approach was unsuitable. For these cores, median ages and 95% confidence intervals were instead calculated using OxCal v4.4 for basal horizons (Ramsey 2009). In some cases (*n* = 7) it was not possible to resolve disagreements in the models between the ^14^C and ^210^Pb data, as the ^14^C date appeared too old in comparison to the ^210^Pb profile for the core. This problem largely arose in very shallow cores, in which measured ^210^Pb had not reached supported background levels. In these cases, we interpret these age‐depth models more cautiously and favoured older basal ages that aligned with our ^14^C data. Binned chronological information from the Bayesian age‐depth models is presented as a function of transect distance in Figure 4, with the raw, calibrated ages from each model provided in Dataset S2.

To calculate rates of lateral expansion (cm yr.^−1^), we divided the distance between each monolith by the difference in calibrated modelled basal ages between monolith cores, as detailed in Equation (1) and outlined in Juselius‐Rajamäki et al. (2023).

(1) $$\text{Lateral Expansion}=\frac{\text{distance between cores}}{\text{basal}\;{\mathrm{age}}_{a}-\text{basal}\;{\mathrm{age}}_{b}}$$

**FIGURE 5:**
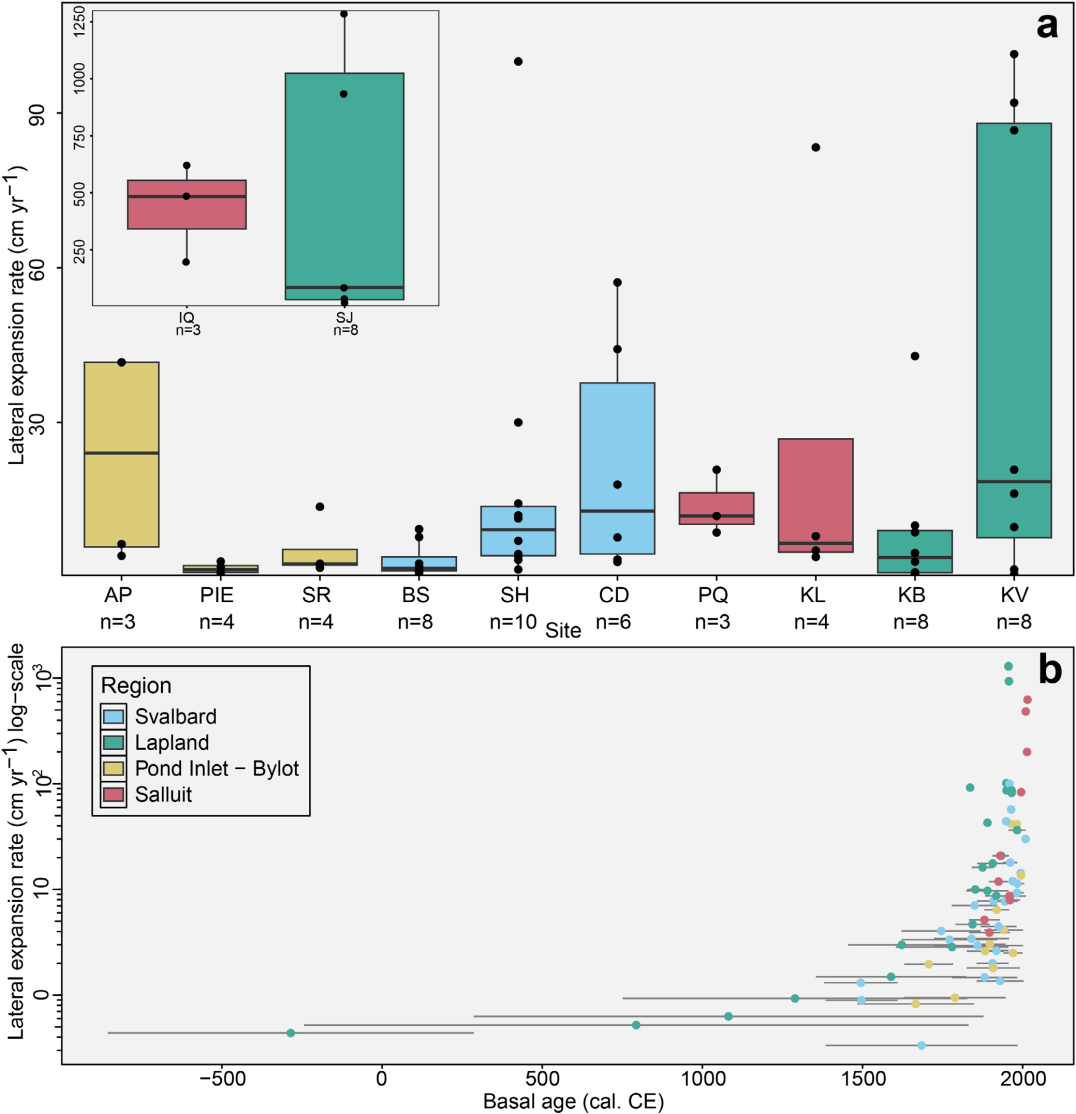
Lateral expansion rates (cm yr.^−1^), from the studied Arctic peatlands, presented as a boxplot (a) and as scatter on a log‐transformed scale, plotted against basal age (cal. C.E.) (b). In panel a: Centerlines indicate median values; boxes indicate the interquartile range (IQR), and whiskers extend up to 1.5 times the IQR beyond the upper and lower quartiles. Sites have been plotted according to temperature, with the coldest on the left and warmest site on the right‐hand side of the plot, increasing from left to right. In panel b: Horizontal lines indicate the two data points from which each lateral expansion rate has been calculated, with the mean of these two basal ages shown as a coloured circle.

We did not calculate lateral expansion rates between outermost cores and peatland edges, because these edges were often not clearly defined from mineral (i.e., non‐peat) substrates, which may lead to some underestimation of very recent or ongoing lateral expansion. We present lateral expansion rates grouped by site location (Figure 5a) and as a function of the mean basal ages of the bounding cores for each section of each transect (Figure 5b).

## 3 Results

### 3.1 Evidence for Peatland Expansion

In all four of our study regions, peatlands have expanded in areal extent since ~1950 cal. yr. C.E., during a period of widespread Arctic warming (See Figure 3a,b and Dataset S1). This expansion is still ongoing, with two thirds (8/12 sites) of our peatland sites evidencing new peat formation after ~1990 cal. yr. C.E. (Figure 3a,b). In the majority of transects (12/17 or 71%), the youngest basal peat ages were recorded at the peatland margin, indicating recent lateral expansion in these sites (Figure 3a,b). Seven of these transects (Angela's Paradise, Salmon River, Pond Inlet‐Bylot, Colesdalen T1 (Svalbard), Iqalutuut Valley, Kissuujaaluk Low, Peat Qilaliariak (Salluit), and Suossjavri T2 (Lapland)) exclusively contained young peats that have developed after ~1800 cal. yr. C.E. For Iqalutuut Valley, Colesdalen T1, and Suossjavri T2 the development and expansion of the site has been particularly recent, with all peats on the studied transect developing after 1900 cal. yr. C.E. and 60% of these peat monoliths initiating after 2000 cal. years C.E. (Figure 4). These very young peat profiles often exhibited low dry bulk density values because they are dominated by poorly humified *Sphagnum* peats with low compaction and high rates of apparent recent vertical accumulation (Figure 4; Dataset S1).

Conversely, for the five remaining transects (Pond Inlet East, Blomstrand T1, Karlebotn T1, Karlebotn T2, and Suossjavri T1), peat monoliths from the peatland margins had older basal dates than at other locations within the site, and in three cases these marginal peats were the oldest dates for the whole transect (SJT2, KBT1, PIE). The peatlands at Blomstrand and Karlebotn appear to have developed much earlier than the younger peatlands described above and consisted of more heavily compacted peats, with respective mean dry bulk density values of 0.24 g cm^−3^ and 0.14 g cm^−3^. Indeed, age‐depth models for four of the six Low Arctic peatlands (Karlebotn, Kevo, Suossjavri (all Lalpland), and Kisssuujaaluk Low and Peat Qilaliariak (Salluit)) indicated slow rates of initial peat accumulation following inception, followed by rapid recent increases since 1900 cal. yr. C.E. (Figure 4; Figure S1ab,ac,aj,as,bc,bp). Furthermore, several sites indicated non‐linear patterns of peatland expansion. At Karlebotn T1 peats became gradually younger towards the centre of the peatland (Figure 3b), while both the Blomstrand and Pond Inlet East transects presented a pattern of basal peats alternating between older and younger ages (Figure 3a). Lastly, at Karlebotn and Pond Inlet East, it is evident that a large amount of peat has accumulated across these two sites prior to 1900 C.E. (Figure 4), with the most recent areas of peat expansion dated to ~1843 cal. yr. C.E. at Karlebotn and ~1837 cal. yr. C.E. at Pond Inlet East.

### 3.2 Rates and Regional Variability of Peatland Expansion

The rate of lateral expansion varied between regions from 0.3 cm yr.^−1^ at Blomstrand, Svalbard, to a maximum of 1300 cm yr.^−1^ at Suossjavri, Lapland. The highest rates of lateral expansion in our study sites were all recorded between young peats that formed after ~1800 cal. yr. C.E. particularly in Low Arctic regions (Lapland and Salluit; Figure 5b). Peats that formed prior to 1800 expanded more slowly, by no more than 3.5 cm yr.^−1^. Our High Arctic study sites exhibited comparatively lower regional rates of lateral peat expansion (Svalbard: mean = 14.5 cm yr.^1^, *σ* = 22.6; Pond Inlet—Bylot: mean = 10 cm yr.^−1^, *σ* = 14.5) than the Low Arctic sites (Lapland: mean = 173 cm yr.^−1^, σ = 384.9; Salluit: mean = 145 cm yr.^−1^, σ = 214.9; Figure 5a). However, this greater average was skewed by very high rates at Suossjavri, Lapland (mean = 469 cm yr.^−1^), Iqalutuut Valley, Salluit (mean = 436 cm yr.^−1^), and Kevo, Lapland (mean = 41 cm yr.^−1^). The remaining Low Arctic sites exhibited comparable lateral expansion rates (mean = 14.9 cm yr.^−1^) to our High Arctic average (Figure 4a), highlighting the substantial intra‐regional variability of rates of peatland expansion in our study. The widest range of lateral expansion values occurred in Lapland, from 0.44 cm yr.^−1^ at Karlebotn to 1300 cm yr.^−1^ at Suossjavri, while peatlands in Svalbard demonstrated more regional consistency, ranging from 3.2 cm yr.^−1^ at Blomstrand to 22 cm yr.^−1^ at Colesdalen (Figure 5a).

## 4 Discussion

### 4.1 Key Findings

This study is the first to constrain long‐term evidence for the lateral expansion dynamics of Arctic peatlands at regional scales, across latitudinal and climatic gradients. The work has highlighted that across four regions of the pan‐Arctic, peatlands show recent expansion since ~1950 cal. yr. C.E., corresponding to a period of widespread Arctic warming (e.g., Rantanen et al. 2022). 75% (9/12 sites) of our peatland sites showed evidence for new peatland formation in the most recent 50 (8 out of 9 of those had new peatland areas form within the last 30 years; Figure 3a,b). The majority of transects (12/17 or 71%) exhibited youngest basal peat ages at peatland margins, seven of which contained recent peats that have formed since ~1800 cal. yr. C.E.

These results were obtained using transect‐based sampling of peat monoliths to establish the rates of lateral expansion from the peatland centres to the edges. This technique has also recently been applied in Finnish peatlands (e.g., Juselius‐Rajamäki et al. 2023, 2025), but our study is the first to apply this technique at scale across various pan‐Arctic regions, allowing for comparisons both within individual regions and between different Arctic regions. Our study provides analysis of spatiotemporal variability in the rate and dynamics of Arctic peatland expansion during the late‐Holocene. Furthermore, our approach combined palaeoecological methods with remote sensing surveys, enabling a comparison of longer‐term peatland dynamics (i.e., through peat monoliths) to be linked to contemporary observations of peatland surface patterns and processes. The following sections provide a discussion of the findings in relation to the three major research questions posed by this study.

### 4.2 Lateral Expansion of Arctic Peatlands Under a Warming Climate

**FIGURE 6:**
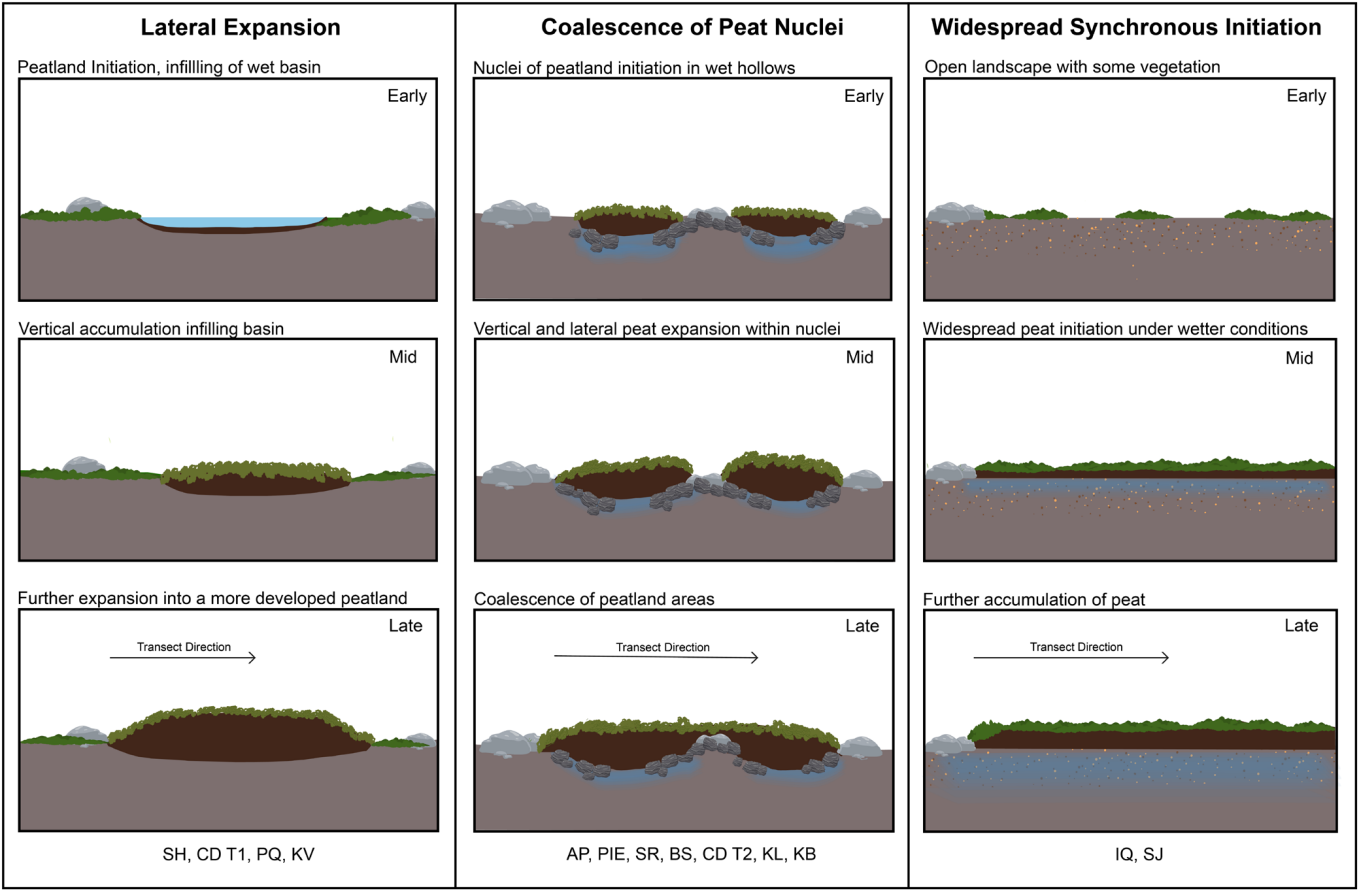
Schematic of different modes of expansion encountered in this study. Modes presented in columns from left to right with site codes detailed below each type.

Our results indicate that the 12 Arctic peatlands in our study presently cover a greater spatial area than at any point during the past 200–300 years (and potentially earlier) and are actively accumulating new peat and C across the full length of our transects (Figure 4). Our finding that lateral expansion of Arctic peatlands is ongoing at a pan‐Arctic scale corroborates previous evidence of site‐specific increases in peat accumulation in Arctic and subarctic regions (e.g., Weckström et al. 2010; Taylor et al. 2019; Sim et al. 2019; Juselius‐Rajamäki et al. 2023; Cleary et al. 2024), although decomposition in these shallow emerging peats may be incomplete (Young et al. 2019). However, our pan‐Arctic analysis has also uncovered substantial variability in the rate of lateral expansion since the LIA, likely relating to regional climatic controls and local‐scale processes. Further, we suggest that this variability is linked to differing modes of peat initiation and subsequently expansion across these rapidly changing landscapes. By synthesising our results from across the pan‐Arctic, we propose three possible models for peatland expansion (Figure 6) within Arctic systems. We outline each phenomenon of peatland expansion and identify relevant case‐studies from across our site network.

#### 4.2.1 Lateral Expansion

This is the simplest model, describing a gradual outwards creep of the peat margins over time, and has been widely observed over longer timescales in subarctic and boreal peatlands (e.g., Weckström et al. 2010; Loisel et al. 2013). In our study, we identified evidence for approximately linear patterns of lateral expansion where the ages of marginal edge peats were much younger than more central, deeper peats (Dataset S1). This phenomenon occurred in several of our study sites, including Stuphallet and Colesdalen (both Svalbard), Kevo (Lapland), and Peat Qilaliariak (Salluit).

#### 4.2.2 Coalescence of Peat Nuclei

Secondly, lateral expansion may also occur as a coalescence of small peat nuclei, which previously developed in disparate pockets in the landscape (e.g., within topographic hollows or low‐centred ice‐wedge polygons) and which eventually accumulated sufficient peat to join into an established Arctic peatland. While this still results in lateral expansion of peat across the landscape, the expansion is non‐linear, particularly where topographic barriers to outwards expansion are present. Evidence of this mode of expansion includes examples where basal peat ages are older at the peat margins than at more central points on the transect (e.g., most clearly evidenced at the margins of Blomstrand, Svalbard, and Karlebotn, Lapland; Figure 3a,b). This process has been previously documented in site‐specific studies in Arctic Alaska (Cleary et al. 2024), subarctic Finland (Juselius‐Rajamäki et al. 2025), and boreal Finland (Mathijssen et al. 2017), but our regional analysis indicates that this is a possible initiation pathway in Arctic peatlands. These peatlands may look very similar to peatlands formed by lateral expansion processes detailed above. However, it is only with analysis of subsurface monoliths and chronologies that it is possible to ascertain that expansion in these instances has occurred multi‐directionally.

#### 4.2.3 Simultaneous Expansion

Finally, we find evidence of almost simultaneous formation of peat across areas of the landscape several tens of metres apart. This process appears to occur in Arctic areas which have rapidly become suitable for peat initiation, through rapid climate shifts or widespread retreat of ice cover, and which exhibit no topographical barriers to peat formation (i.e., low lying, poorly drained landscapes). This means that peat can simultaneously form across large areas of the warming landscape, instead of expanding outwards from a single point of origin. The most notable example of simultaneous expansion occurred at Iqalutuut Valley, where the shallow peat monoliths on this transect (IQM1–IQM7) exclusively formed between ~2007–2016 cal. yr. C.E., despite being located 24 m apart (Figures 3b and 4). Similarly, at Suossjavri, Lapland, basal peats of adjacent monoliths on transect T1 (SJM3, SJM5, and SJM7) and T2 (SJM13 and SJM15) formed during ~1955–1958 cal. yr. C.E. across distances of 25 m and 19 m, respectively, in different parts of the mire (Figure 3b).

### 4.3 Spatiotemporal Variability in Lateral Expansion Rates of Pan‐Arctic Peatlands

The variable modes of Arctic peatland expansion outlined above (Figure 6) resulted in a wide range of expansion rates when these values were calculated using the assumption that peatland expansion occurs unidirectionally from the peatland centre towards the margins. Indeed, it is often challenging to determine where the centre of Arctic peatlands is actually located because these sites are heterogenous complexes of intact and degrading permafrost landforms, thermokarst pools, fens, and bogs (Table 1, Figure 2). Our analysis of monolith transects taken from peatland edges provides a useful method for analysing the marginal rates of expansion, but the complexity of Arctic peatlands resulted in relatively few examples of strictly linear gradients of increasingly shallow, younger peats towards the peat margin. However, irrespective of the method used to calculate peatland expansion, our findings overall support the hypothesis that Arctic peatlands have expanded in recent decades, which aligns with a period of increased warming in these regions.

When considering only those Arctic peatlands providing evidence of more typical, linear modes of lateral expansion, we found no clear trends in lateral expansion rates between regions, due to the substantial variability introduced by climate, local topography and site‐specific controls, including permafrost dynamics and landscape hydrology. For example, the basal ages of our marginal Salluit peats were substantially younger than marginal peats from Lapland sites (Figure 3b). Monoliths taken from peatlands in the Canadian Arctic particularly demonstrated young peat initiation ages and very recent expansion (> 1950 cal. yr. C.E.) of poorly humified *Sphagnum* moss peat (Figure 5b; Table 1). This may be indicative of a threshold response to climate warming, particularly as the Canadian peatlands in this study are colder (mean annual temperature (MAT) range = −6.3°C–13.5°C) than those in Lapland (MAT range = +0.1°C–1.5°C) and Svalbard (MAT range = −3.2°C–4.6°C) (Table 1). Recent productivity estimates derived from satellite records over 30 years for many of the peatlands included in this study (Crichton et al. 2022) show a combination of warming and wetting climates, with rising spring, summer, and previous autumn temperatures identified as important controls on satellite‐derived proxies of peatland productivity. Indeed, the rate of late 20th‐century temperature increases has been higher in Svalbard and parts of the Canadian archipelago, driven by Arctic amplification feedbacks (e.g., You et al. 2021). The cold baseline climates of the study sites on the Canadian archipelago may therefore mean that a summer temperature threshold, where plant productivity becomes sufficient for renewed peat accumulation, may have been surpassed later for these peatlands than in other regions, such as Lapland (Table 1), where the studied peatlands developed earlier and are now more spatially extensive (Figure 2).

Climate observations indicate that increases in temperature are not directly correlated with precipitation in Arctic regions (e.g., Box et al. 2019), so temperature‐dependent controls on peatland expansion should be considered in tandem with moisture availability. The latter is vital for plant growth, but also for the restricted decomposition of plant litter, and thus peat formation, under warming climates. Rising temperatures can increase convection‐driven precipitation, but also increase evapotranspiration, cause shifts from snowfall to rainfall, and alter the timing and magnitude of seasonal snowmelt (Box et al. 2019), with important implications for the landscape hydrology of Arctic environments. Evidence from the literature underlines that moisture/hydrology is a limiting factor for all modes of peatland expansion (Juselius‐Rajamäki et al. 2025; Ruppel et al. 2013), but especially for the non‐linear patterns of expansion identified in this study. We assert that hydrology is likely a main control at the Canadian sites Pond Inlet East, Salmon River, and Peat Qilaliariak, which are stream‐associated mires (Table 1, Figure 2) and the latter of which is an entirely new peatland that appears to have formed since the LIA (Figures 3b and 4). These emerging peatlands have formed in pockets of enhanced surface moisture, which drives plant growth even under cold climates, and any lateral expansion onto the bare mineral soil at the edges of the peatland has only occurred where margins have become wetter. The transect at Peat Qilaliariak spanned the entire radius from the peatland centre and exhibited shallow peat depths (< 10 cm) (Figures 3b and 4), a small areal footprint (Figure 2), and a clear linear pattern of lateral expansion (mean rate = 13.8 cm yr.^−1^; Figure 5). This may indicate a positive feedback for Arctic peatlands where expansion is moisture limited and the low hydraulic conductivity of Arctic peats (Fewster, Morris, Swindles, Ivanovic, et al. 2023) further inhibits surface drainage, thereby increasing waterlogging in Arctic landscapes and enabling incremental, areal increases to the suitable area for additional peat formation. Such ecohydrological feedbacks may also explain why shallow peats have formed in the Iqalutuut Valley, Salluit, atop a porous sandy substrate. The importance of local‐scale, hydrological controls on peat formation at this site is emphasised by the much older ages of peats found on the opposite bank of the Guichaud River, which were previously dated to ~4500 cal. yr. BP, although this may also relate to the earlier emersion of this river terrace (Ouzilleau Samson et al. 2010). This provides further evidence that climate warming is not solely responsible for enabling the recent expansion of some Arctic peatlands. Site‐specific factors must therefore be considered when analysing spatiotemporal patterns in large‐scale datasets.

An important secondary control on the rate of expansion of emerging peatlands is topography, once the climate is suitable. Topographic barriers to expansion were evident at all Svalbard sites: steep cliffs delineate the peatland margins at Blomstrand and Colesdalen, while Stuphallet has formed in a morainic basin, bordered by a moraine wall to the north and a steep cliff to the south and west of the site. This may explain the reduced rates of expansion at these sites (Figure 5). Across the High Arctic, the presence of glaciers and ice sheets has prevented peat initiation for much of the Holocene. The Angela's Paradise site on Bylot Island is situated on a proglacial delta, a landscape which has been opened for ecological colonisation by the continued retreat of glacier C79 (Dowdeswell et al. 2007). By contrast, the peatlands indicating the highest rates of lateral expansion in our study, Suossjavri (Lapland) and Iqalutuut Valley (Salluit), have both expanded across open, gently sloped substrates (Figure 3b). This suggests that in the absence of topographical barriers, new peat areas can form simultaneously across large areas of Arctic landscapes as soon as bioclimatic conditions become suitable. This mode of peatland expansion has the potential to rapidly transform large areas of the Arctic within decades. Further spatial modelling is now needed to identify areas which have the potential to rapidly accumulate new peat in this manner, namely low‐lying, gently sloped, and poorly drained environments in warming and wetting Arctic regions.

Lastly, permafrost and the presence of ice lenses or ice wedges present further complications for lateral expansion processes, particularly for those sites with degrading palsas (e.g., Kevo, Karlebotn) and high and low‐centred polygons (e.g., Angela's Paradise, Kissuujaaluk Low). Permafrost aggradation can uplift peats, creating raised landforms with drier surfaces (Seppälä 2011), and form hydrological barriers which impede vertical and/or horizontal drainage (Helbig et al. 2013). The presence of a widespread, impermeable permafrost table may present a plausible mechanism for the recent peat initiation at Iqalutuut Valley, Salluit, which formed atop a porous sandy substrate. Permafrost thaw can subsequently drive surface subsidence and collapse (Olefeldt et al. 2021), surface inundation (Swindles et al. 2015), and increase the hydrological connectivity of the landscape (e.g., Haynes et al. 2018). Further, ecological succession associated with the surface collapse and inundation of degrading permafrost peatlands can increase rates of vertical peat accumulation in permafrost‐free fens and bogs (Treat et al. 2016). In regions of discontinuous permafrost, permafrost is now limited to peatland areas where organic soils provide insulation from rising air temperatures, due to low dry bulk densities and thus low thermal conductivities (Kujala et al. 2008). Ongoing permafrost thaw was also evident within many of our study sites, indicated by the presence of surface cracking and thermokarst ponds at Karlebotn and Kevo in Lapland (Figure 2). Permafrost thaw may become an increasingly important driver of peatland initiation, expansion, and stability in future because suitable climates for permafrost peatlands are projected to disappear entirely from some Arctic regions within decades (Fewster et al. 2022; Ruuhijärvi et al. 2022; Leppiniemi et al. 2023).

### 4.4 Implications for Radiative Forcing of Pan‐Arctic Peatlands

Our finding of recent areal expansion of Arctic peatlands across large spatial gradients has important, concomitant implications for understanding the radiative forcing potential of pan‐Arctic wetlands and their C sequestration capacity. Our analysis shows that Arctic peatlands are expanding, even if this is not always laterally, and that peat is now accumulating in greater areas of these Arctic environments than at any point in the late‐Holocene. The increasing spatial extent of Arctic peatlands suggests a great potential for the northern high latitudes to become increasingly productive C sinks in future decades, which could partially offset some losses from the expected climate‐induced degradation of peatlands at tropical and temperate regions (Swindles et al. 2019; Gallego‐Sala et al. 2018). Furthermore, the direction, magnitude, and speciation (e.g., methane or carbon dioxide) of peatland greenhouse gas emissions are closely tied to local ecohydrology, with previous studies observing considerably higher methane emissions from inundated, permafrost‐free fens than bogs and intact permafrost landforms (Varner et al. 2021; Holmes et al. 2022). Given that the shallow peats found at the marginal areas of our study sites have only recently initiated (Figures 3a,b and 4), many of these emerging peatland areas are likely to be minerotrophic, particularly Peat Qilaliariak where spring water seems an important driver of peat initiation. The high rate of peat accumulation in these cores (Figure 4) suggests our studied peat margins are highly productive areas, with peat surfaces now rapidly elevating beyond the influence of groundwater sources. However, a previous study of the lateral expansion of Lompolovuoma, a subarctic fen in Finnish Lapland, found that marginal peats can persist as fens for several centuries, due to the hydrological conditions of the wider catchment, which can result in a net climate warming effect, due to methane production (Juselius‐Rajamäki et al. 2025). Some marginal areas at Lompolovuoma have recently undergone a fen‐bog transition (Juselius‐Rajamäki et al. 2025), which will likely increase their C sink capacity, and thus lead to a net climate cooling effect in the longer term. However, this example highlights the need for caution in drawing simplistic conclusions about the potential radiative forcing effects of the observed expansion of these Arctic peatlands, particularly given the pronounced spatiotemporal variability and non‐linear patterns evident in our results. To more explicitly elucidate the radiative forcing of these expanding peatland margins, further research is needed to quantify contemporary greenhouse gas fluxes (e.g., chamber measurements and eddy covariance gas exchange monitoring) and to reconstruct the recent palaeoecology (e.g., plant macrofossil records) and palaeohydrology (e.g., testate amoebae records) of these emerging ecosystems.

Our observations of rapid Arctic peatland expansion may not necessarily continue indefinitely in future decades, particularly if suitable climates for peat formation shift into areas with less favourable topography, for example the presently glaciated, mountainous regions of Ellesmere Island (Canada) and Greenland. Climate warming has driven rapid, recent retreat of glaciers in these regions (References needed). Our study sites in Svalbard (Blomstrand, Colesdalen and Stuphallet) and Bylot Island (Angela's Paradise) illustrate evidence that peatland expansion is already ongoing in some recently deglaciated Arctic environments. However, once glaciers and snowpacks disappear and permafrost thaws, the persistence of Arctic peatlands will become reliant on precipitation inputs. Observed Arctic temperatures have increased almost four times as quickly as the global average since 1979 (Rantanen et al. 2022), a trend which is projected to continue during the 21st century and drive increases in precipitation and shifts from snow to rain (Davy and Outten 2020). Projections of precipitation and the timing and duration of snowmelt are less certain than temperature‐derived variables (Meredith et al. 2022), but both appear critical for Arctic peatland development. Our findings suggest that such climatic changes will likely render Arctic regions productive environments for peatland initiation and expansion, provided that suitably low‐lying, gently‐sloped, and poorly‐drained topography is available for ecological colonisation. Over the longer term, the poleward expansion of peatlands under a warming climate will also be latitudinally constrained by the northernmost extent of terrestrial land.

Previous large‐scale modelling has projected a shrinking of pan‐Arctic wetland extents and C sink capacity under scenarios of strong future warming, because of temperature‐driven increases to evapotranspiration and respiration (Müller and Joos 2021; Chaudhary et al. 2022; Zhao and Zhuang 2023). However, such modelling is restricted by inadequate mapping of peatland extents at high latitudes (Chaudhary et al. 2020), and the dynamic range of processes that have driven recent Arctic peatland expansion, as outlined in our study (Figure 6), is poorly represented in large‐scale, land‐surface models. This further highlights the critical need for the continued collection of empirical, field‐based data to support satellite‐ and modelling‐based approaches to understand ecosystem responses to climate change. The timescales of new peat accumulation are also important to consider when attempting to reconcile the radiative forcing potential of such large‐scale ecosystem shifts. Additionally, it remains unclear if similar modes and rates of peatland expansion are ongoing in high latitude regions not included in our study, including Siberia, northern Alaska, and indeed Antarctica. Recently published studies have identified a similar coalescence of emerging peat patches in the northern foothills of the Brooks Range in Arctic Alaska (Cleary et al. 2024), while widespread, sustained greening of the Antarctic Peninsula has occurred since 1986 C.E. (Roland et al. 2024). The alignment of these findings with our own study, which itself has encompassed a broad latitudinal gradient, suggests that the patterns of peatland expansion we have observed are likely reflective of broader changes occurring across Arctic regions. To better constrain the plausible extent of future peatland expansion in the Arctic, further bioclimatic modelling is now needed that explicitly incorporates the peat expansion processes identified in this study.

Establishing the timing and rate of lateral expansion uncovers only part of the C accumulation histories of Arctic peatlands because vertical peat accumulation can occur non‐linearly (Belyea 2009). Indeed, across our study sites and transects, our age‐depth models indicate higher apparent peat accumulation rates in recent decades, with many of the highest rates recorded after ~2000 cal. yr. C.E. (Figure 4), which could indicate a climate‐induced productivity response to recent warming (Gallego‐Sala et al. 2018). However, due to the challenges in reconciling net rates of ongoing vertical peat accumulation from peat cores (see Young et al. 2019), we do not interpret vertical rates further in this study. To more confidently quantify past rates of vertical peat accumulation, core profiles may be reconstructed using process‐based models that account for changes in decomposition through time (e.g., Frolking et al. 2010; Treat et al. 2021). While beyond the scope of this study, integrating confidently resolved vertical peat accumulation histories, remote sensing data on recent greening and moisture changes (e.g., Crichton et al. 2022, 2025), and long‐term lateral expansion data such as that provided in our study could offer valuable new insights into the C sink capacity and radiative forcing potential of Arctic peatlands in a changing and warming climate.

## 5 Conclusions

Our study presents new observational evidence that Arctic peatlands have expanded in spatial extent, often rapidly, during recent decades across multiple regions. At a continental scale, this peatland expansion appears to be linked to rising temperatures, because the primary period of lateral expansion at all sites occurred during the post‐industrial period of anthropogenic climate warming. Therefore, we expect this expansion to be occurring across the Arctic. However, our analysis of 12 Arctic peatlands spanning a latitudinal gradient from the Low to High Arctic regions of Europe and Canada indicated substantial spatiotemporal variability in the timing, rate, and modes of lateral expansion. This variability is indicative of additional non‐climatic constraints on peatland extent, including local topography, hydrology, and the presence of permafrost landforms. While a few Arctic peatlands have expanded linearly from the peatland centre towards the edges, others appear to have formed through simultaneous peat initiation across large spatial areas, while others have formed as disparate peat patches within the landscape and have eventually coalesced. Our work has presented new evidence for recent expansion of peatlands in the Arctic which now needs to be explicitly included in process‐based models to better understand whether the creation of new peatland areas and peatland expansion will persist under future climate warming.

## Author Contributions

**J. Handley:** writing – review and editing, writing – original draft, visualization, formal analysis, data curation, investigation, methodology, validation. **R. E. Fewster:** writing – original draft, writing – review and editing, visualisation, formal analysis, data curation. **T. G. Sim:** writing – review and editing, visualisation, formal analysis, data curation. **S. Hodson:** data acquisition, data curation, writing – review and editing. **B. Parker:** data acquisition, data curation, writing – review and editing. **K. Crichton:** writing – original draft, writing – review and editing, visualisation, formal analysis, data curation. **D. Charman:** conceptualisation, investigation, writing – review and editing, funding acquisition. **K. Anderson:** conceptualisation, investigation, writing – review and editing, supervision, funding acquisition. **M. Garneau:** conceptualisation, writing – review and editing, funding acquisition. **M. Väliranta:** conceptualisation, writing – review and editing, investigation, funding acquisition, data acquisition. **D. W. Beilman:** writing – review and editing, investigation, data acquisition. **G. T. Swindles:** writing – review and editing, investigation, data acquisition. **M. Aquino‐López:** writing – review and editing, formal analysis. **M. Blaauw:** writing – review and editing, formal analysis. **X. Comas:** writing – review and editing, investigation, data acquisition. **E. Levesque:** writing – review and editing, investigation, data acquisition, funding acquisition. **V. Maire:** investigation, data acquisition, funding acquisition. **H. Addis:** data acquisition, data curation, writing – review and editing. **M. Amesbury:** conceptualization, writing – review and editing, data curation. **D. Fortier:** writing – review and editing, funding acquisition. **M. Mleczko:** writing – review and editing, investigation, data acquisition, formal analysis. **A. Gallego‐Sala:** conceptualization, investigation, funding acquisition, writing – original draft, methodology, project administration, supervision, resources, writing – review and editing.

## Funding

This work was supported by the Natural Environment Research Council, NE/S001166/1; Leverhulme Trust, RPG‐2021‐354; Quaternary Research Association. Department of Agriculture and Rural Development, Northern Ireland. Department of Agriculture, Environment and Rural Affairs, UK Government; Polar Continental Shelf Program; Natural Sciences and Engineering Research Council of Canada; the Network of Centers of Excellence of Canada; Polar Knowledge Northern Scientific Training Program.

## Conflicts of Interest

The authors declare no conflicts of interest.

## Supporting information


Dataset S1:

Dataset S2:

**Figure S1:** gcb70684‐sup‐0003‐FigureS1.pdf.

## Data Availability

The data that supports the findings of this study are available in Supporting Information of this article.
