# Supplementary figures and images for: Pan‐Arctic Peatlands Have Expanded During Recent Warming
Source: Glob Chang Biol. 2026 Feb 13;32(2):e70684. doi: 10.1111/gcb.70684 (PMC12902909; doi:10.1111/gcb.70684)

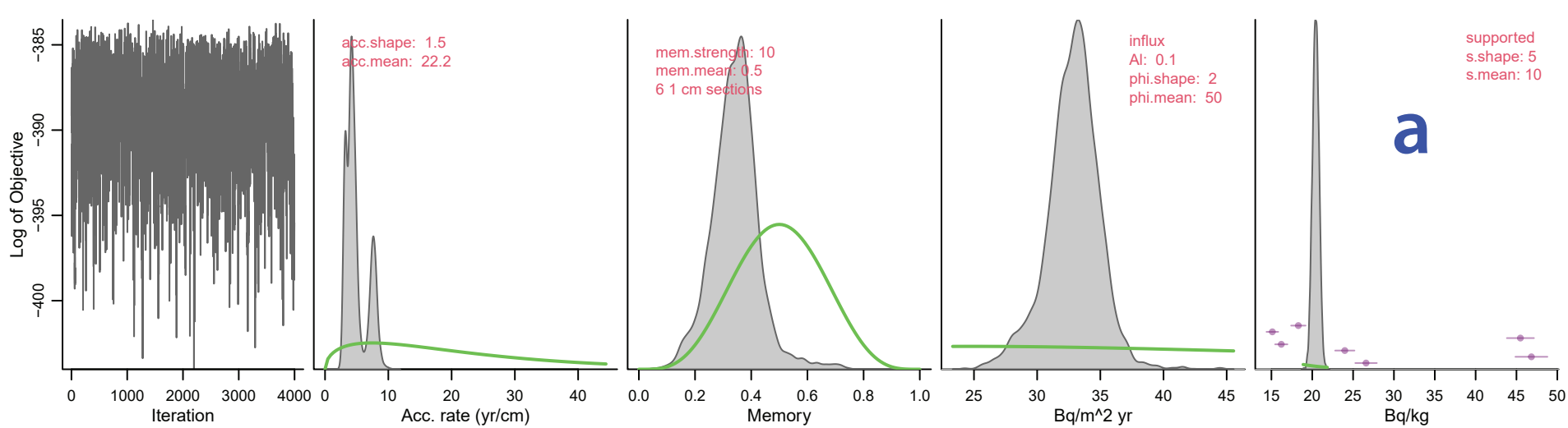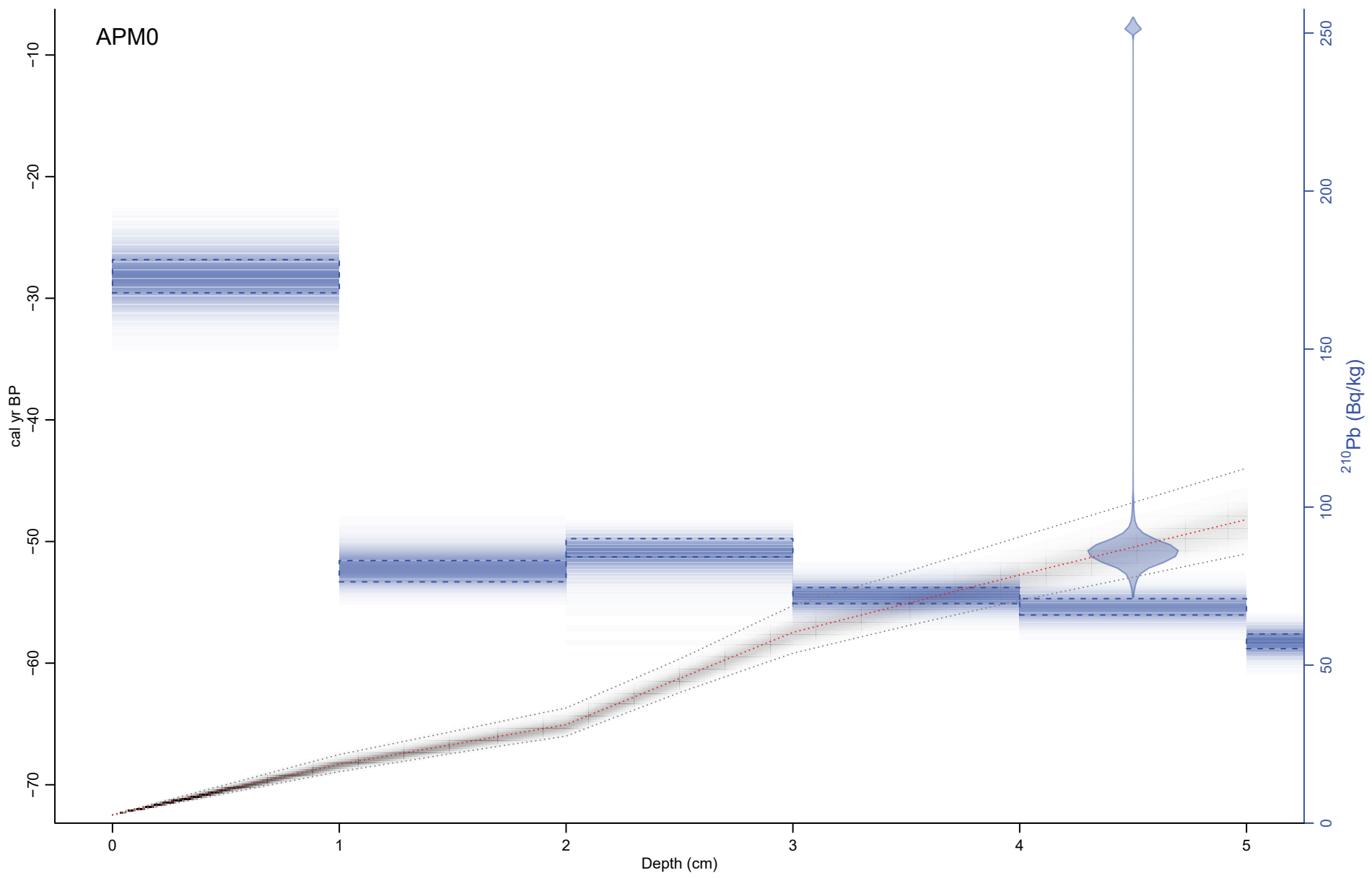

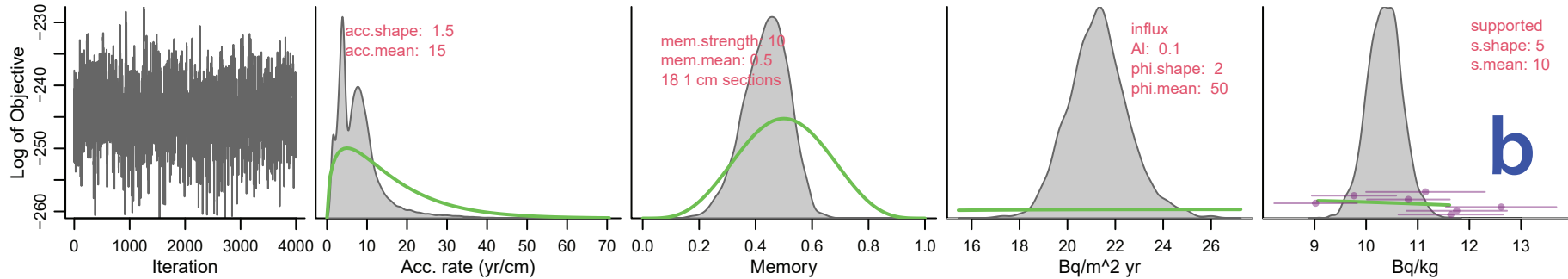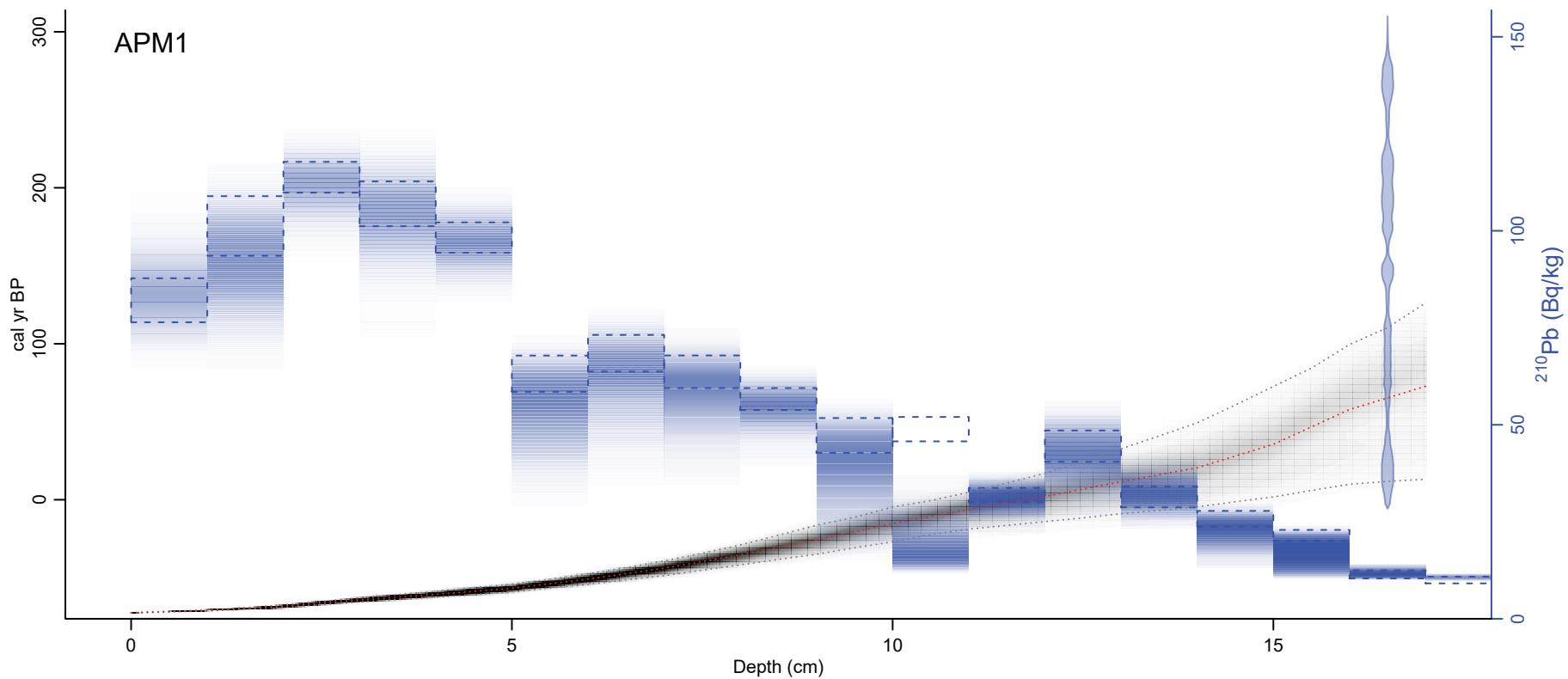

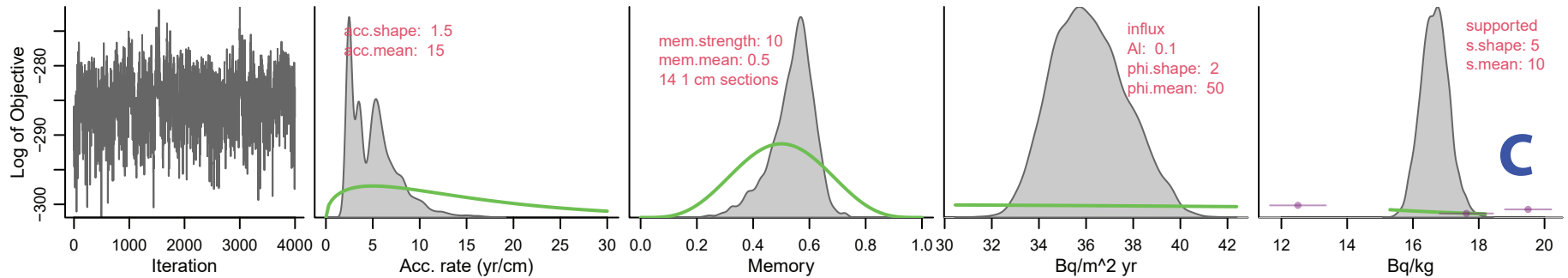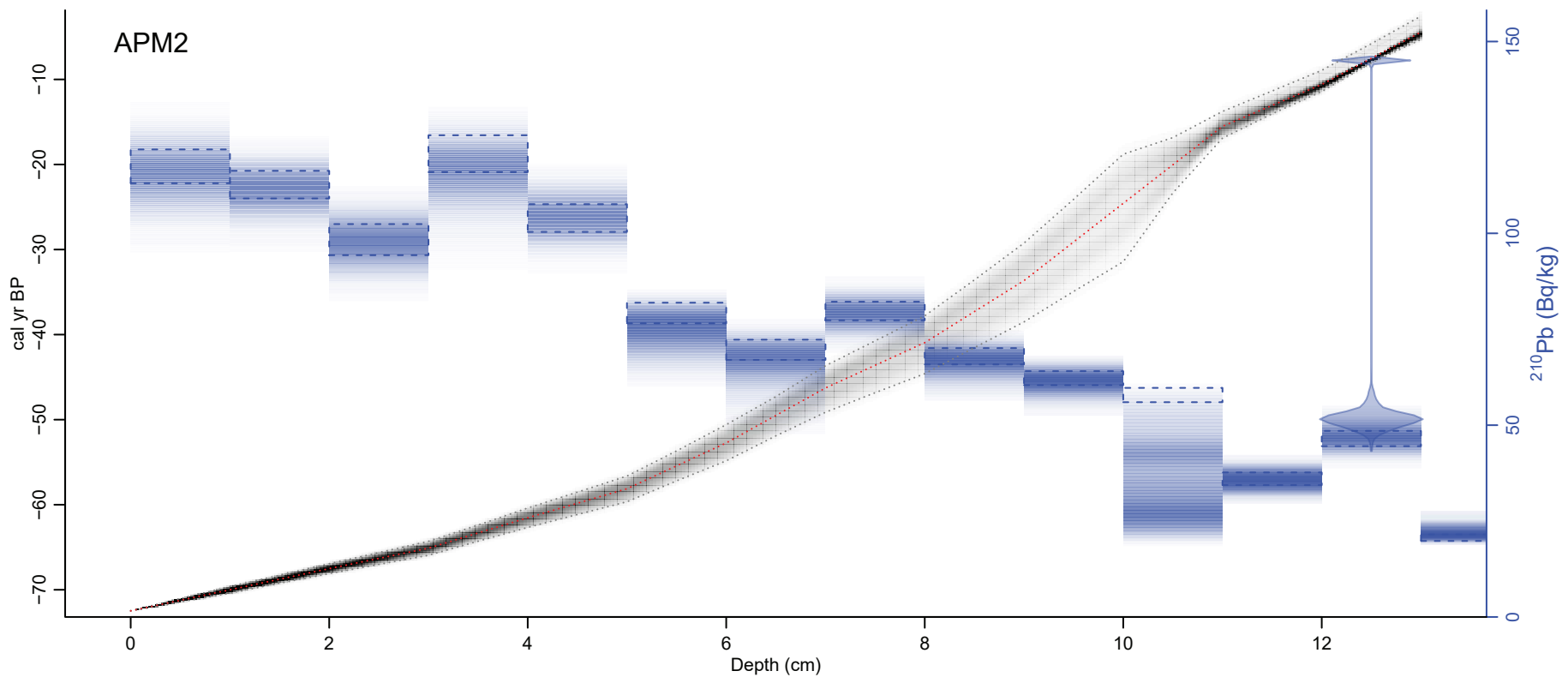

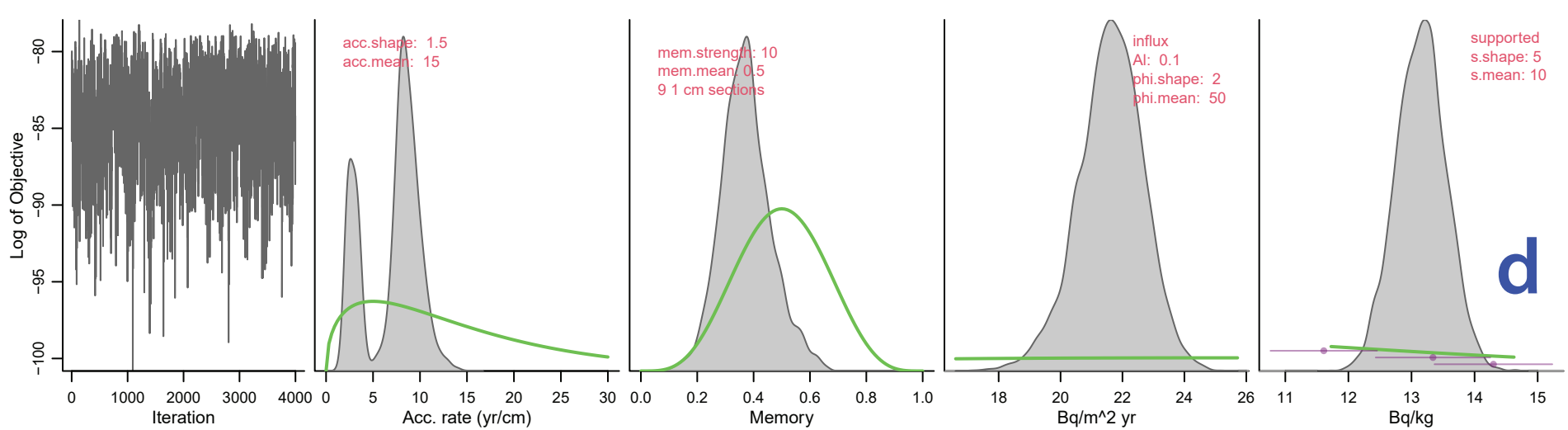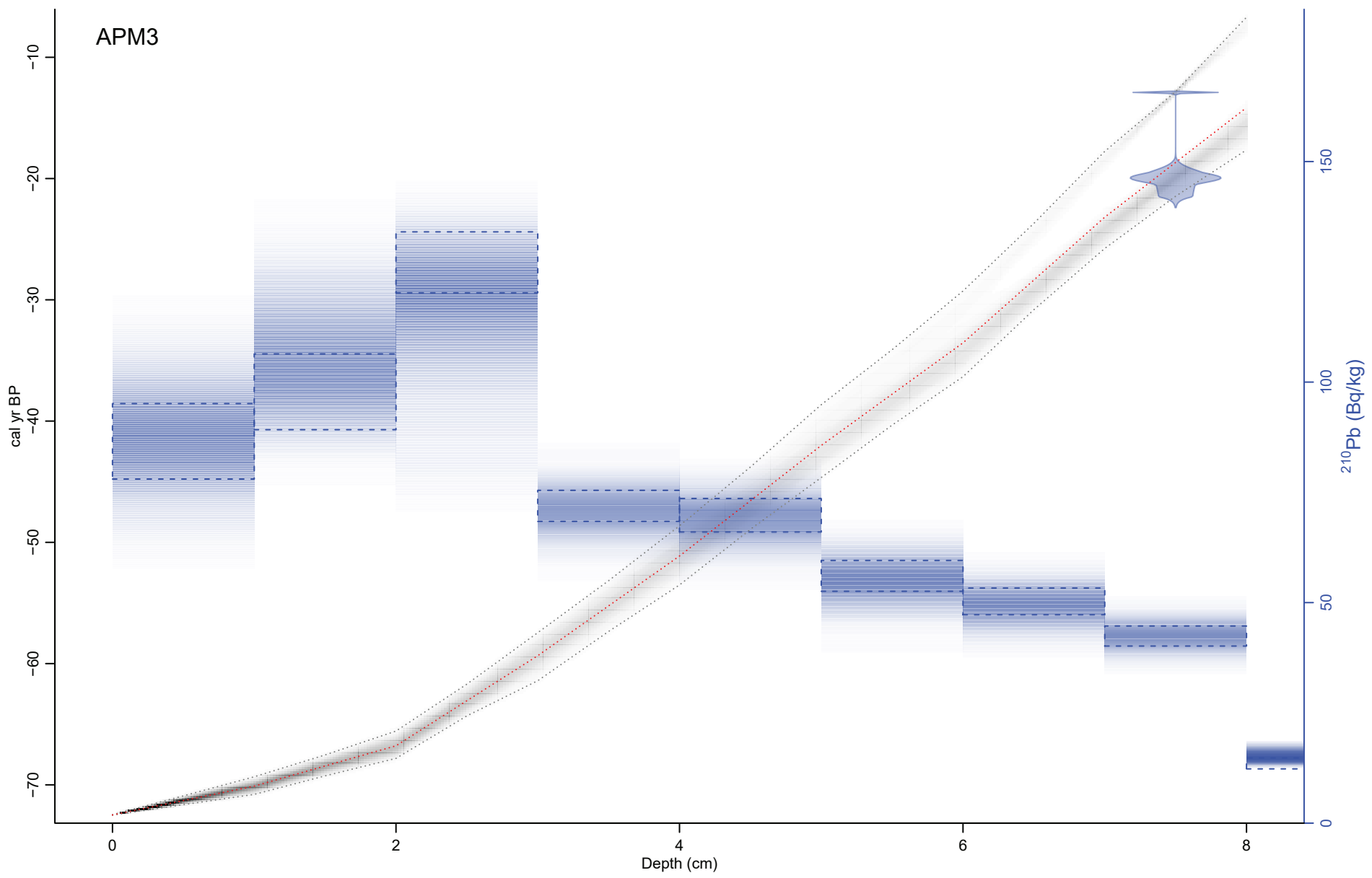

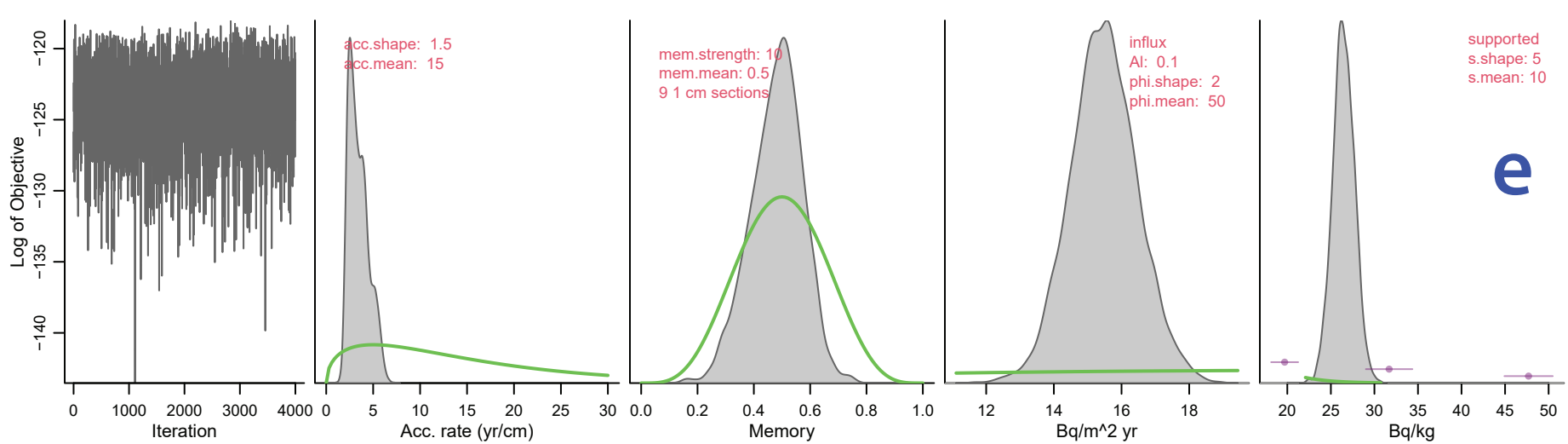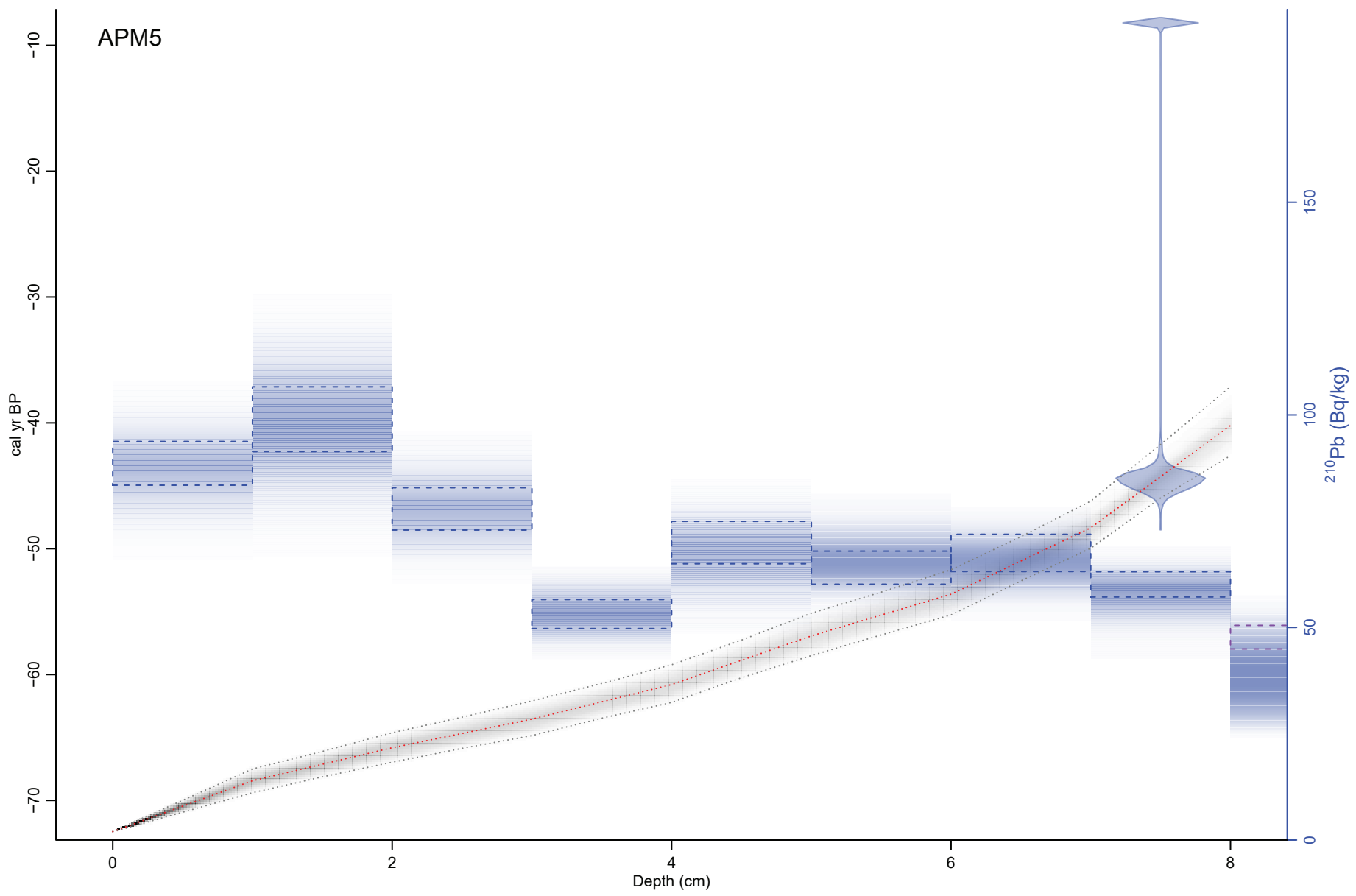

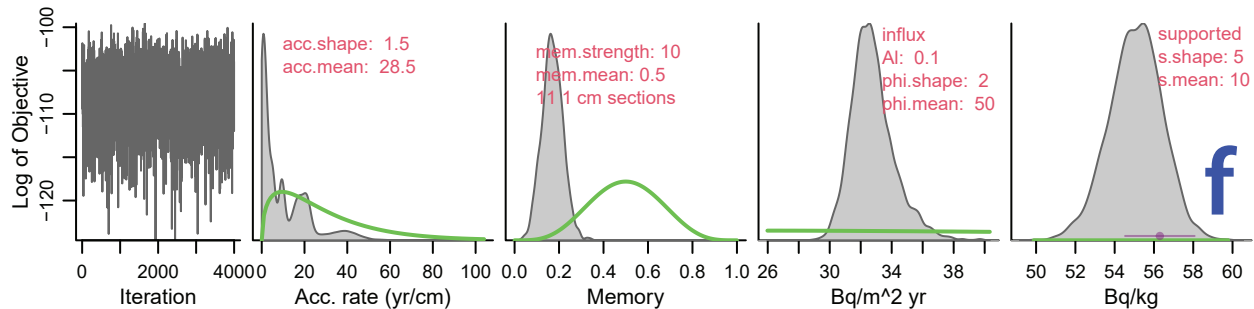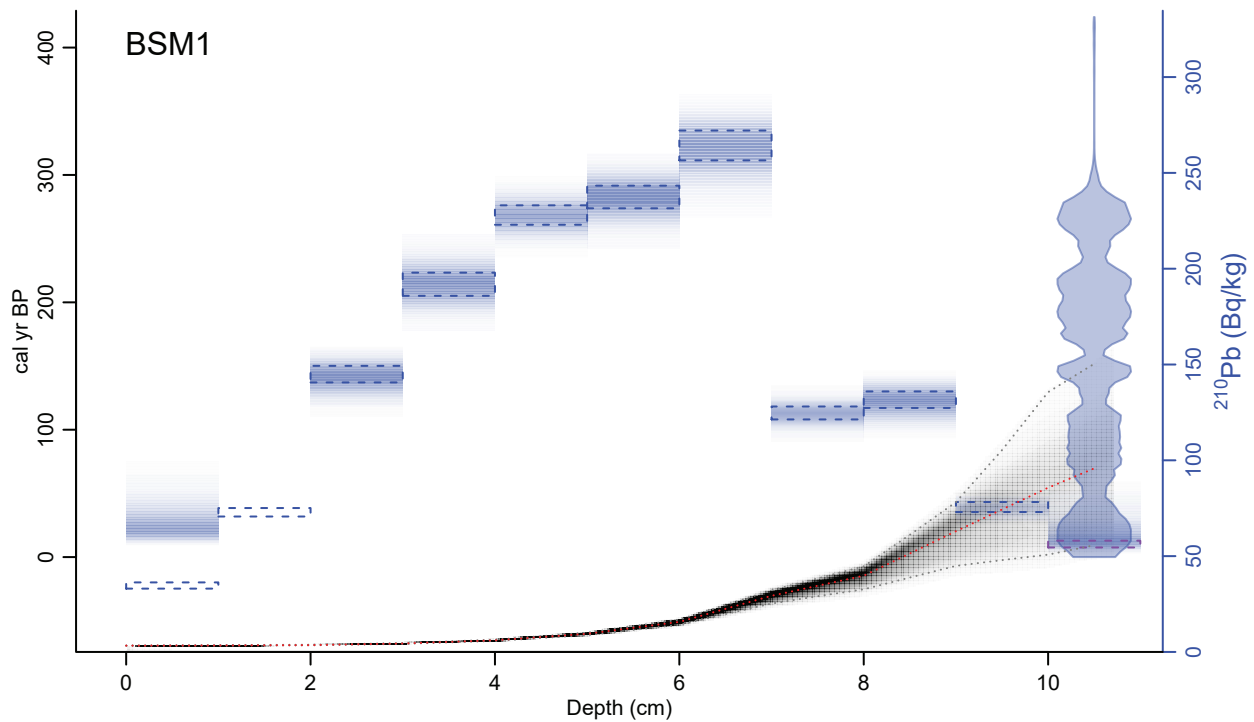

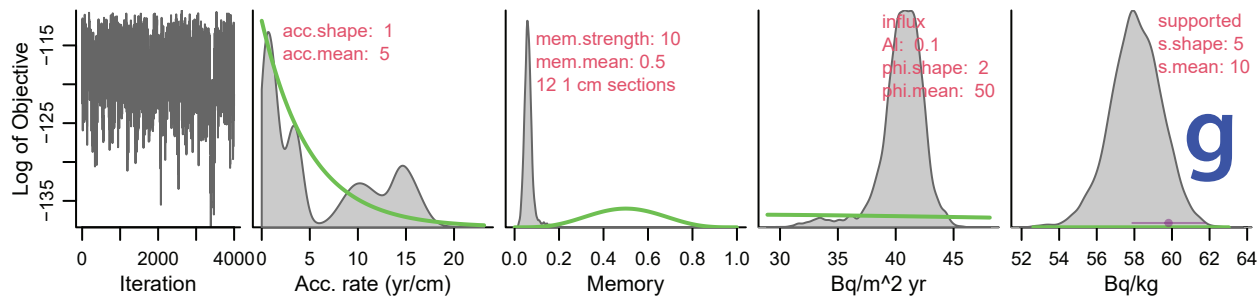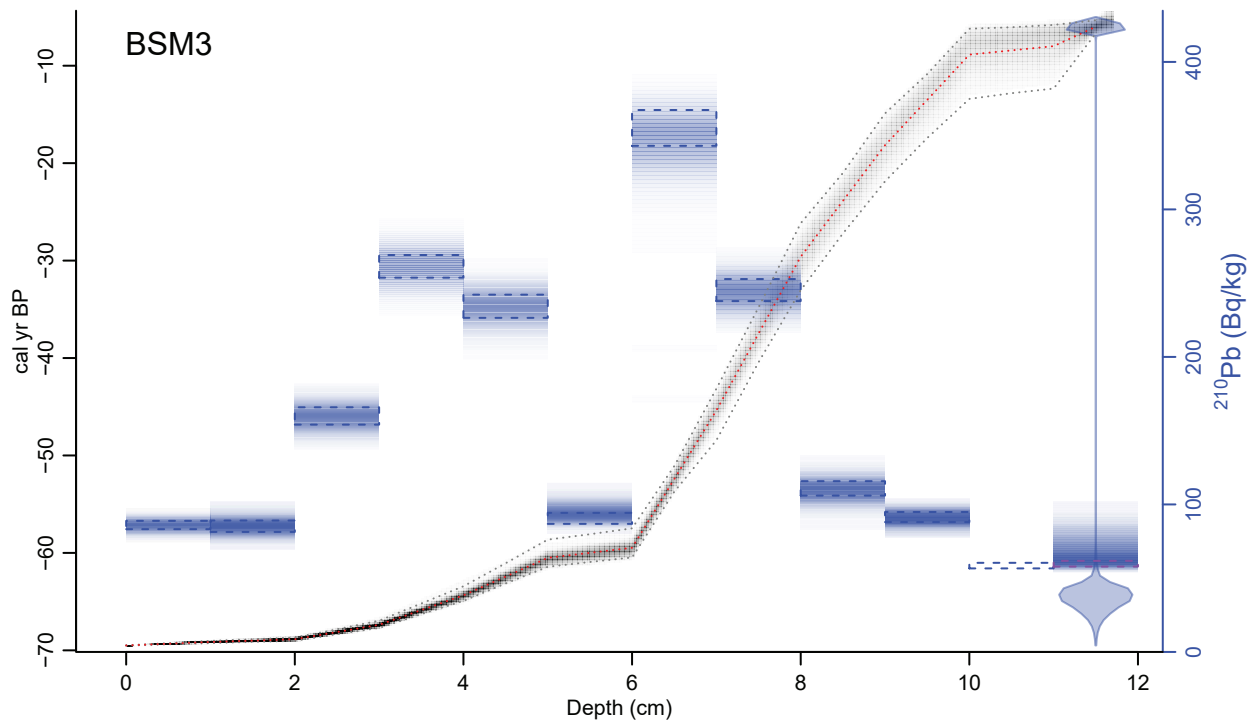

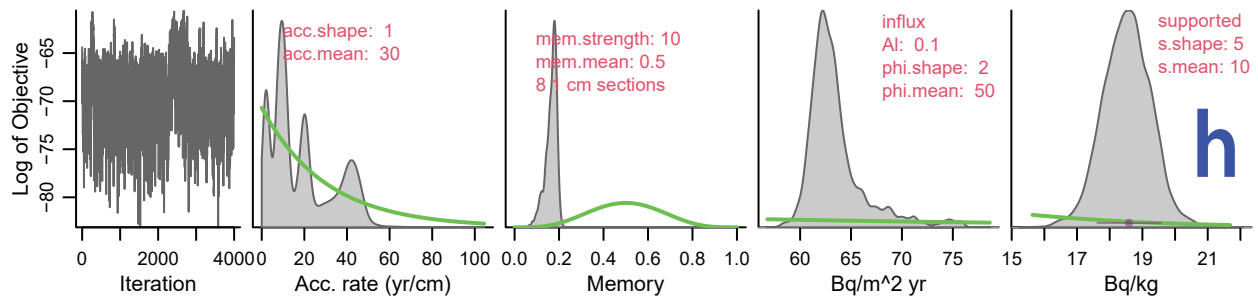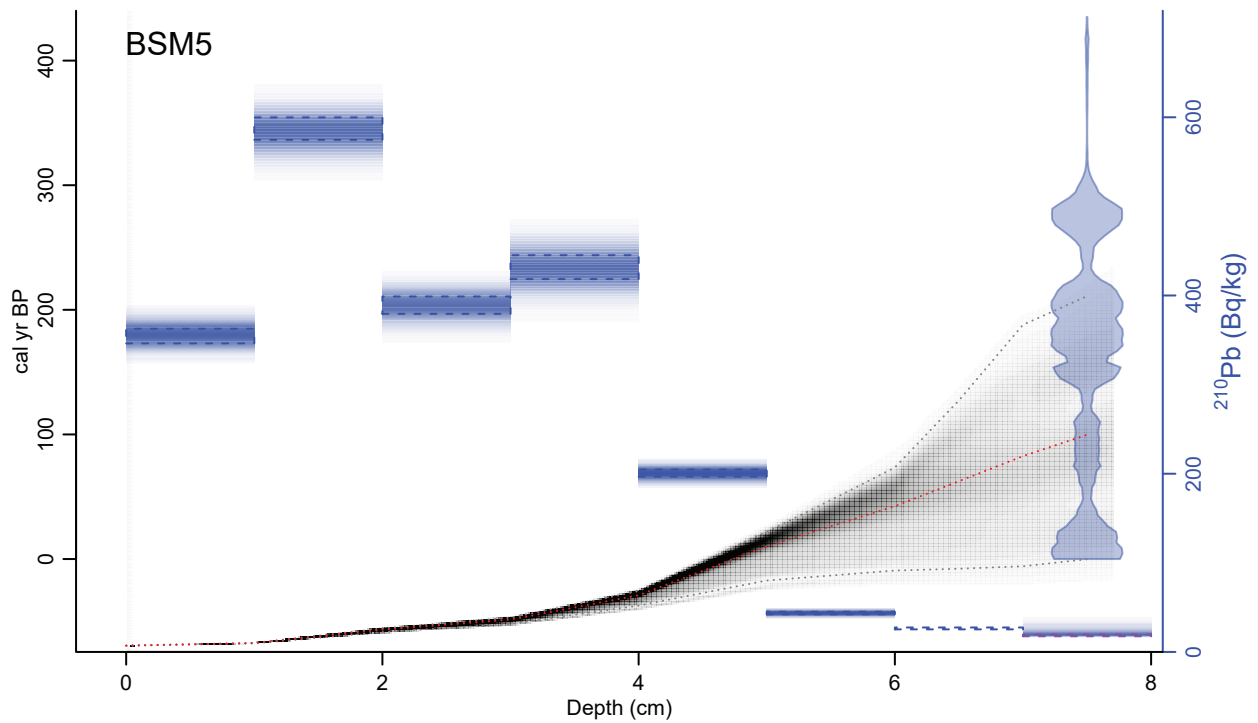

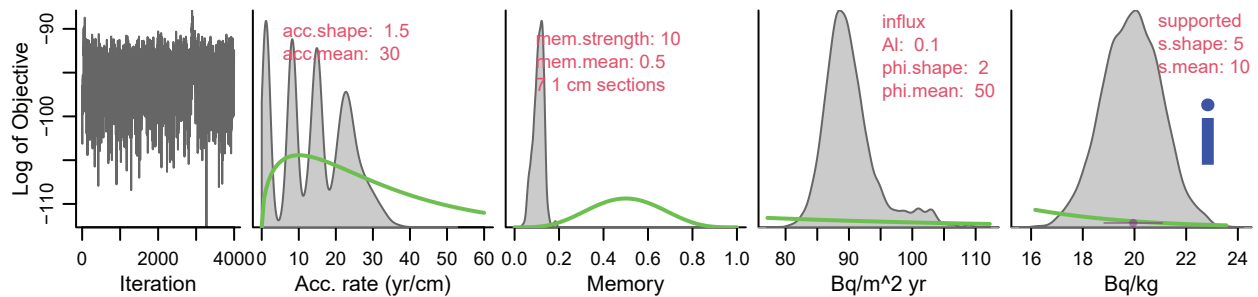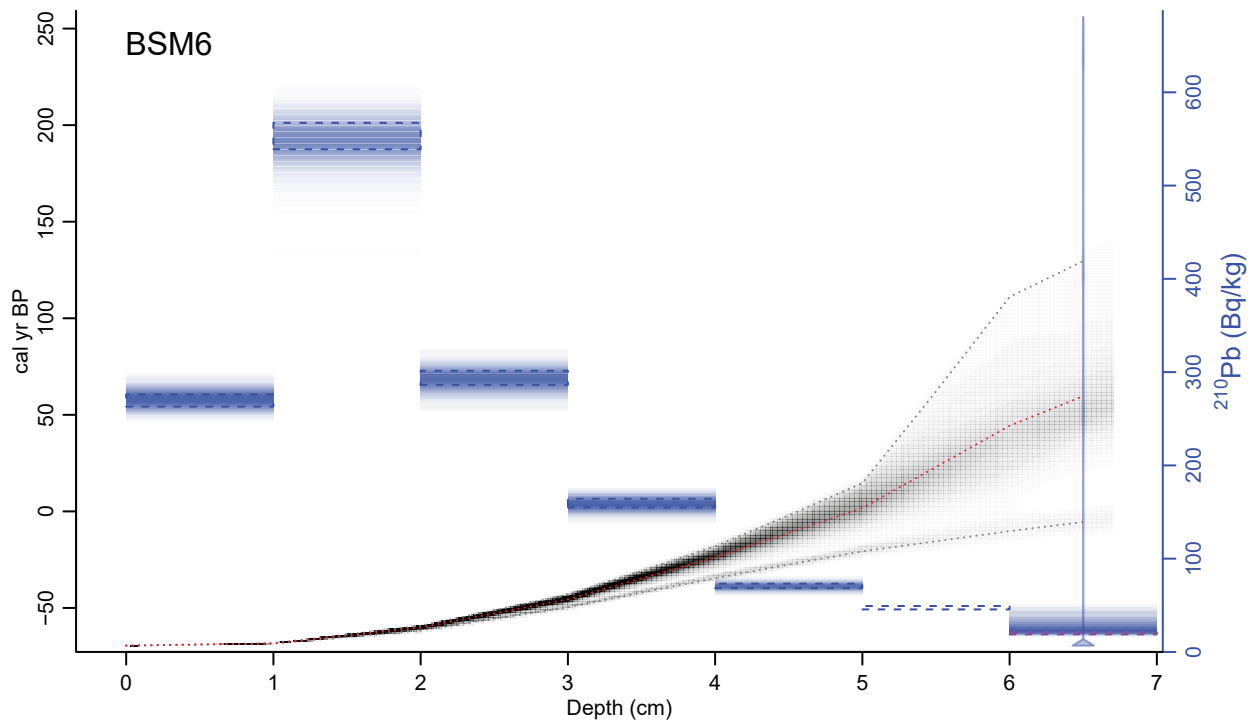

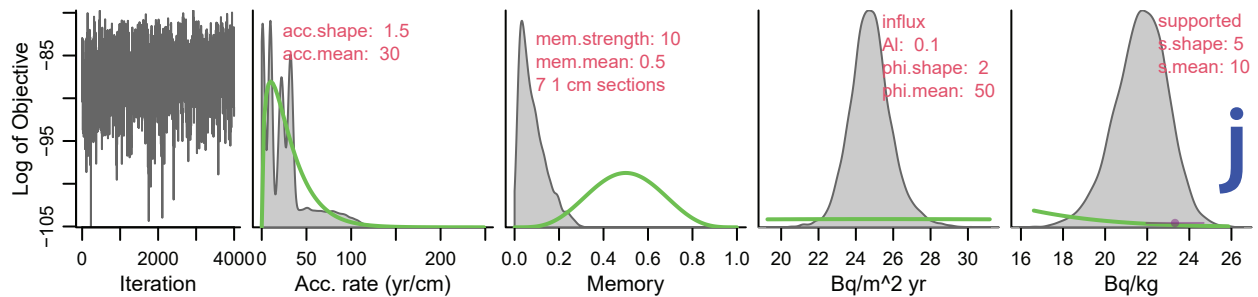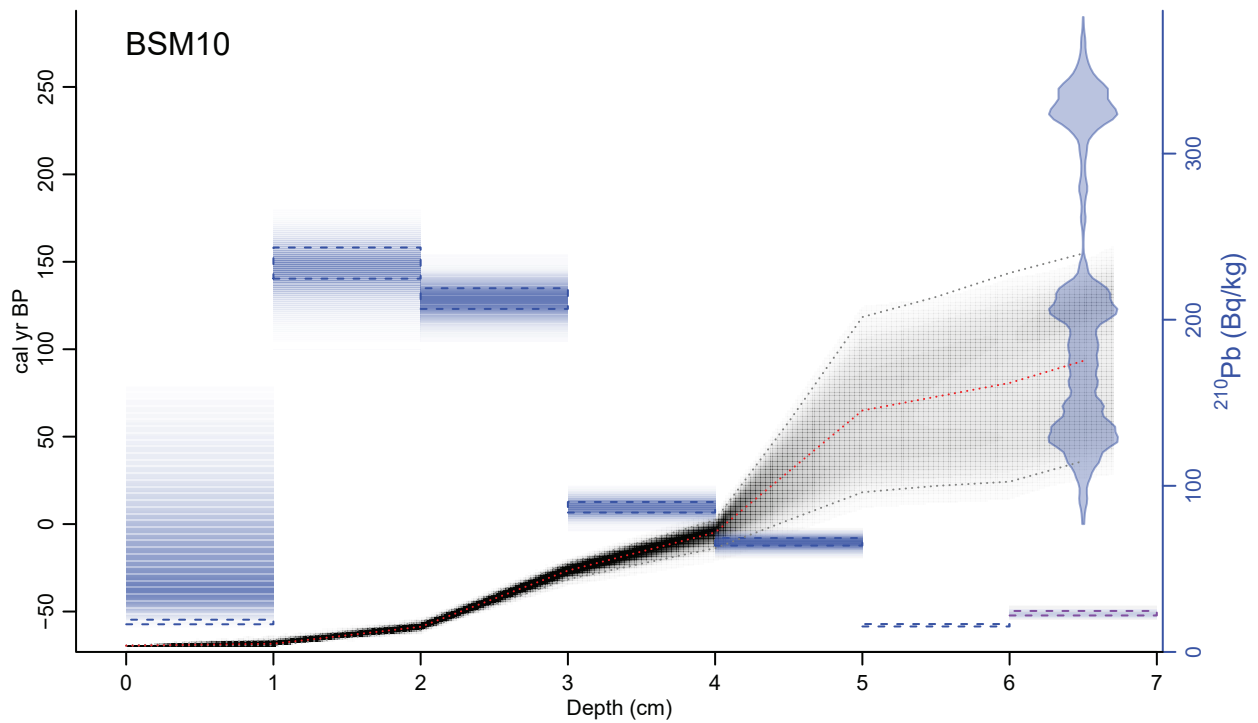

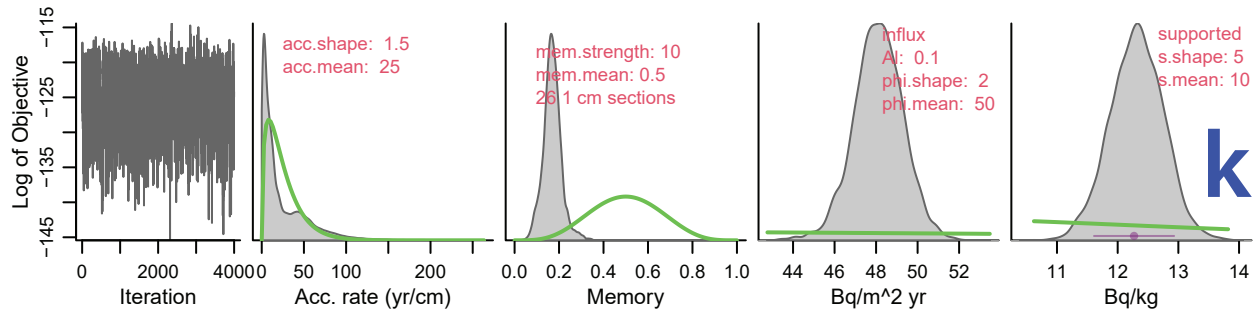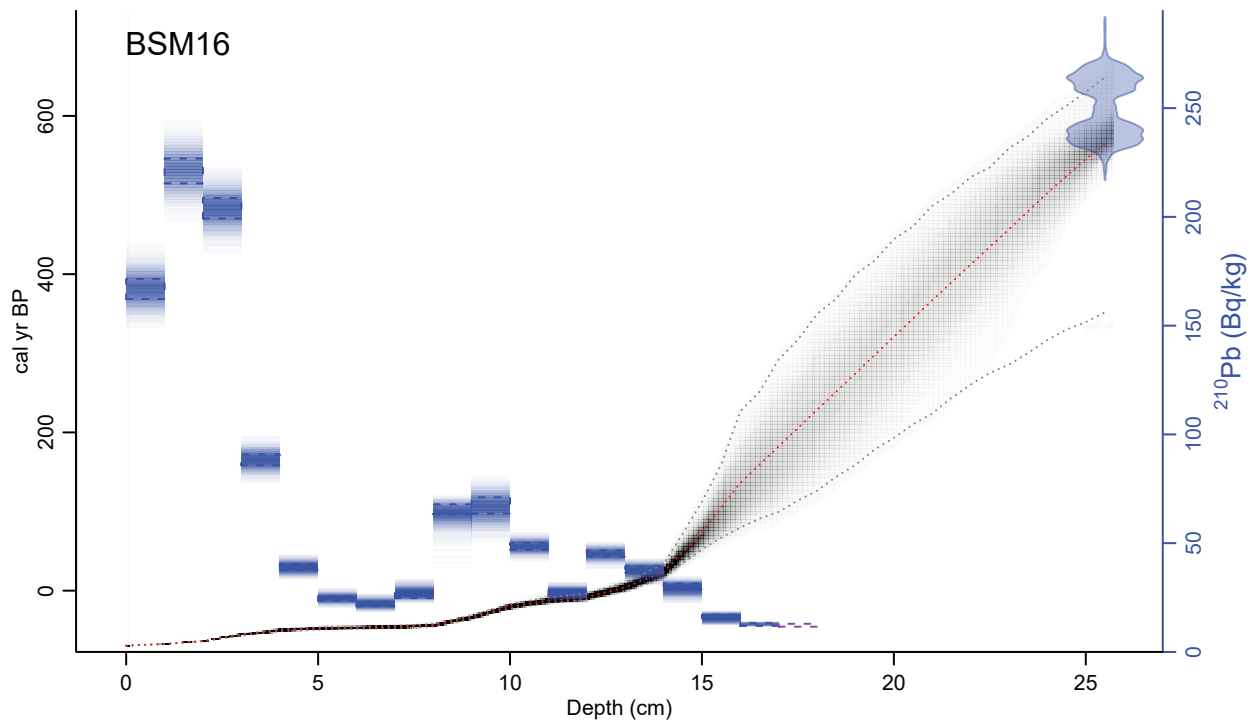

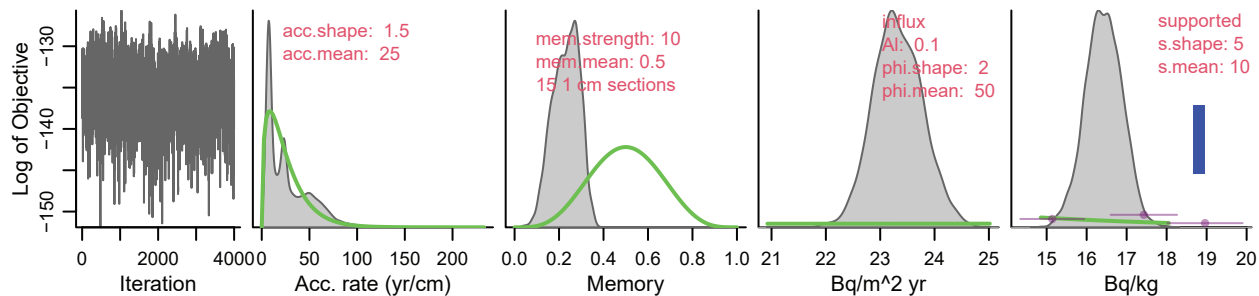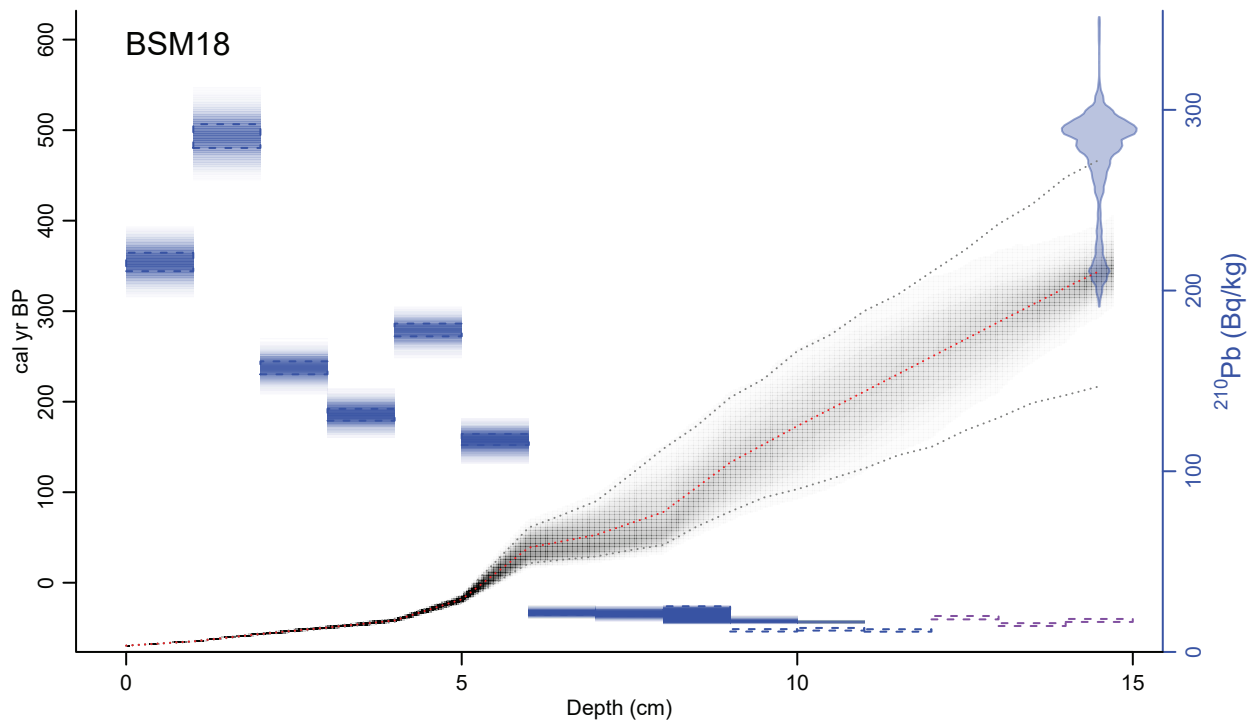

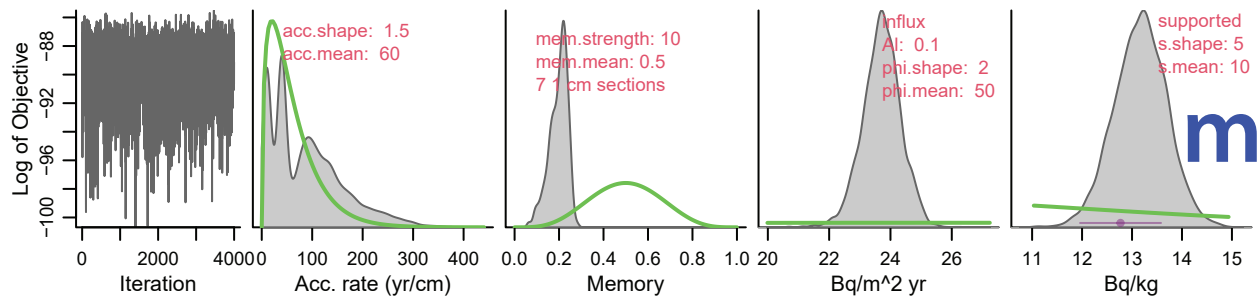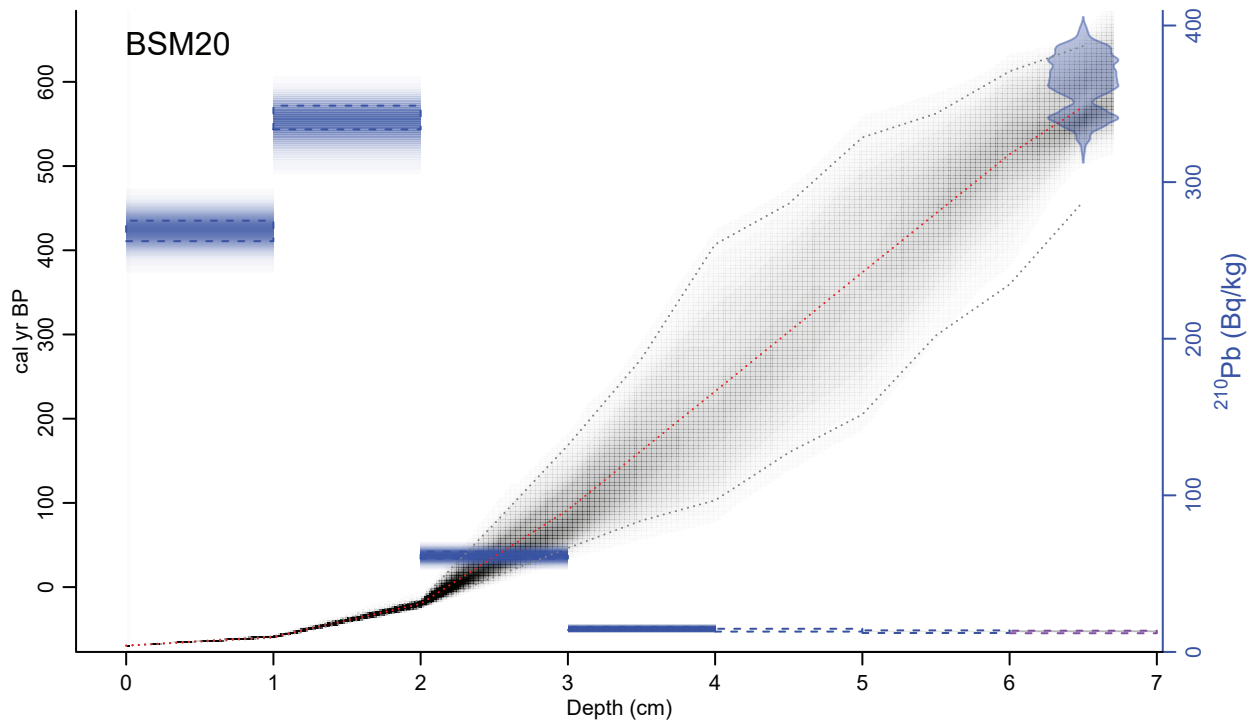

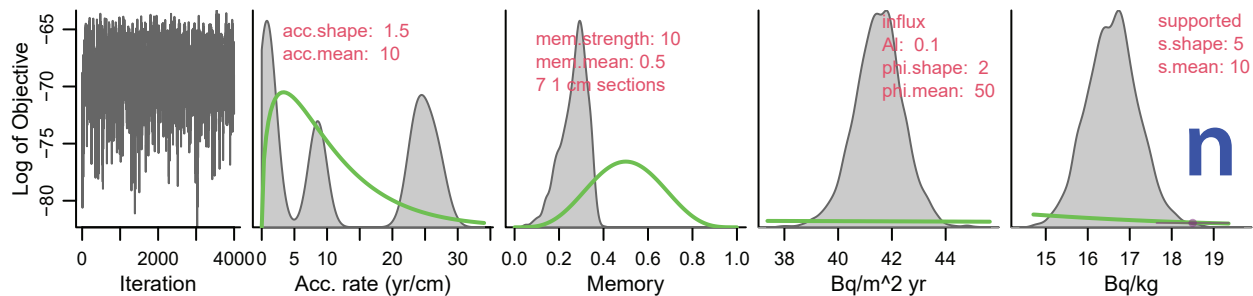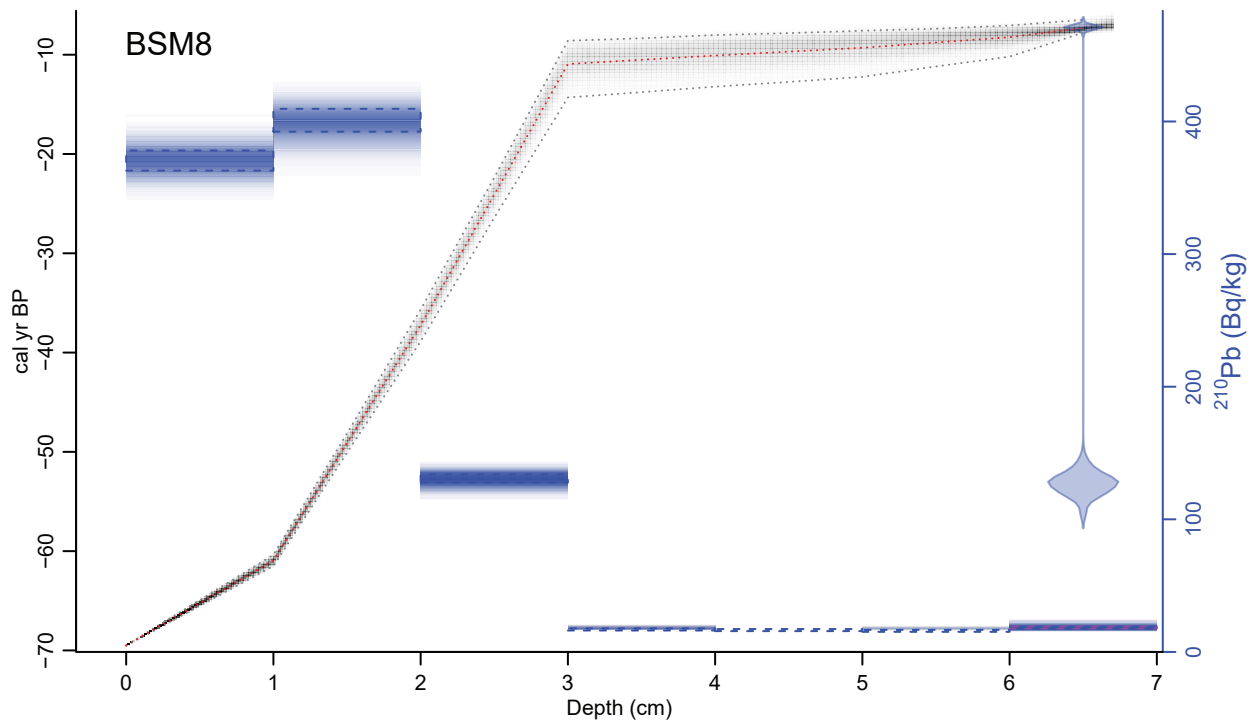

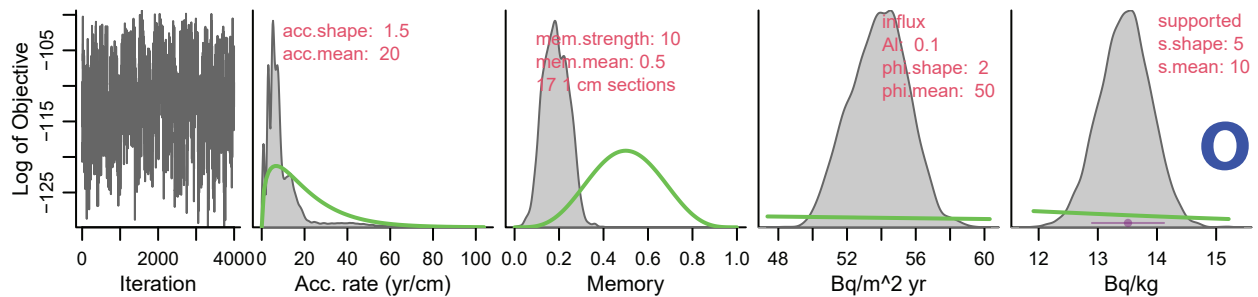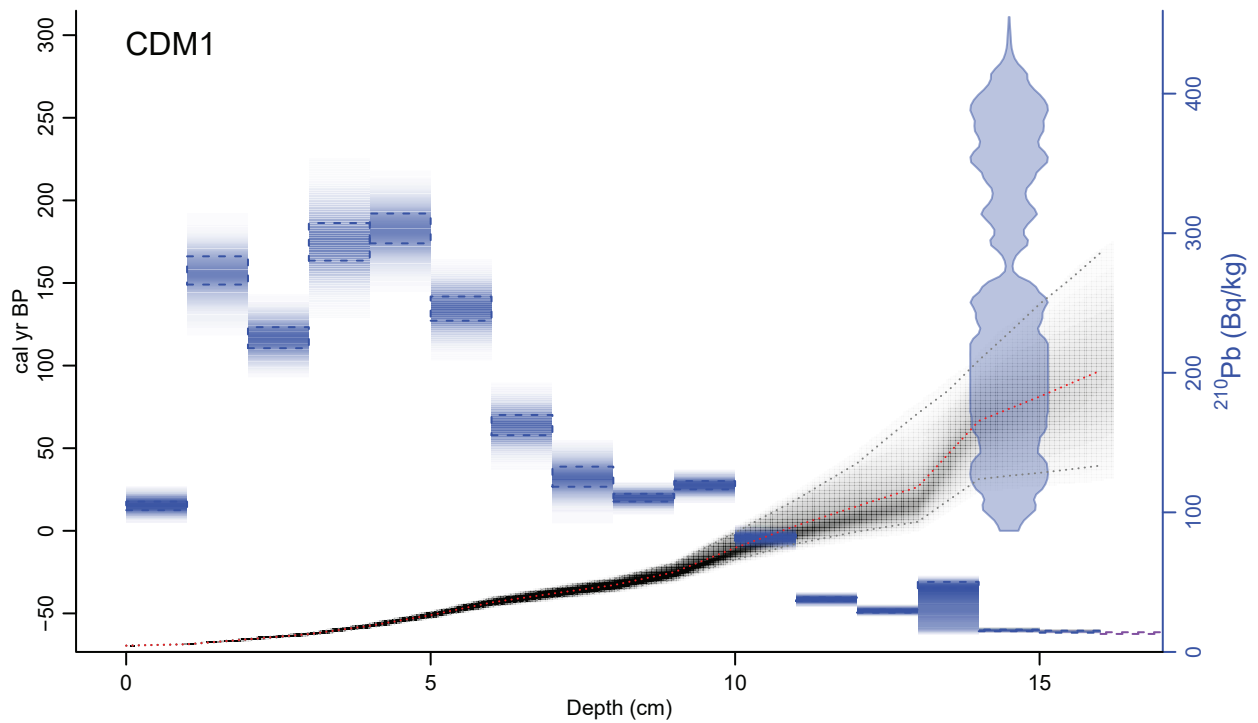

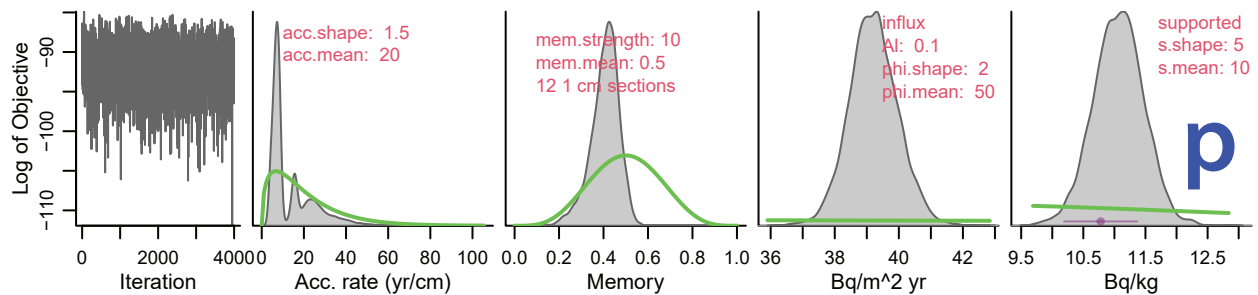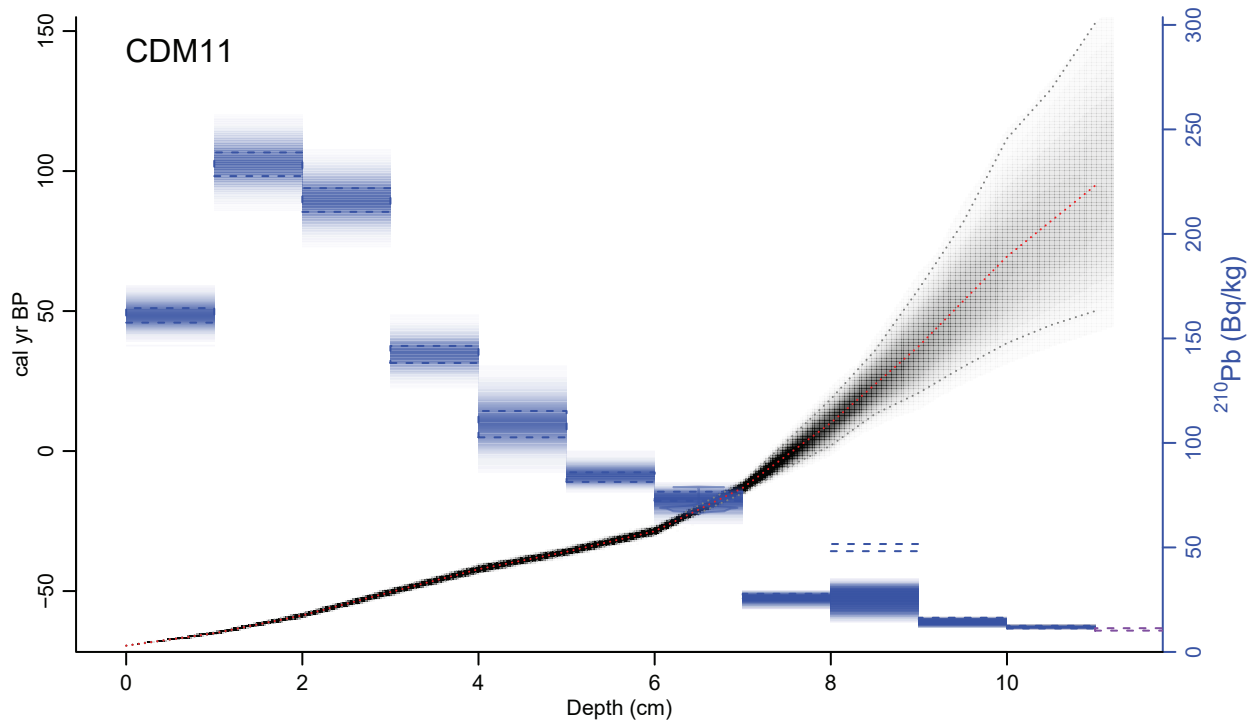

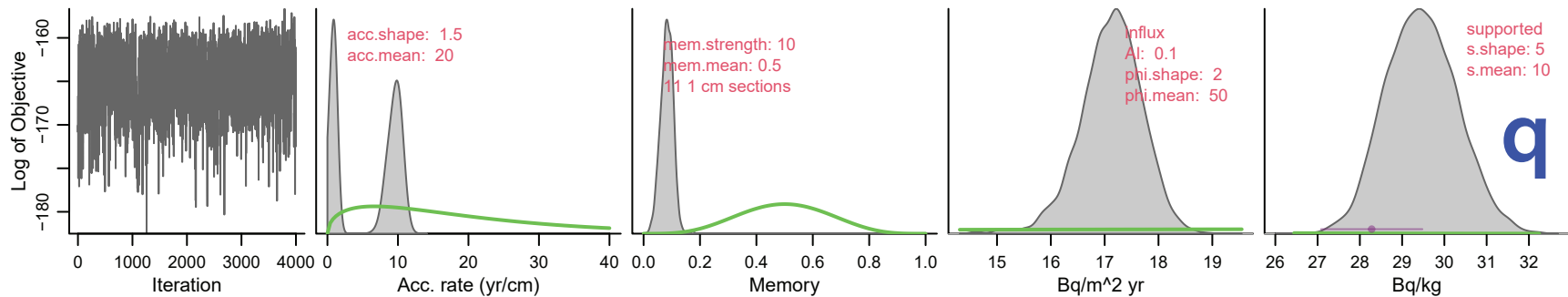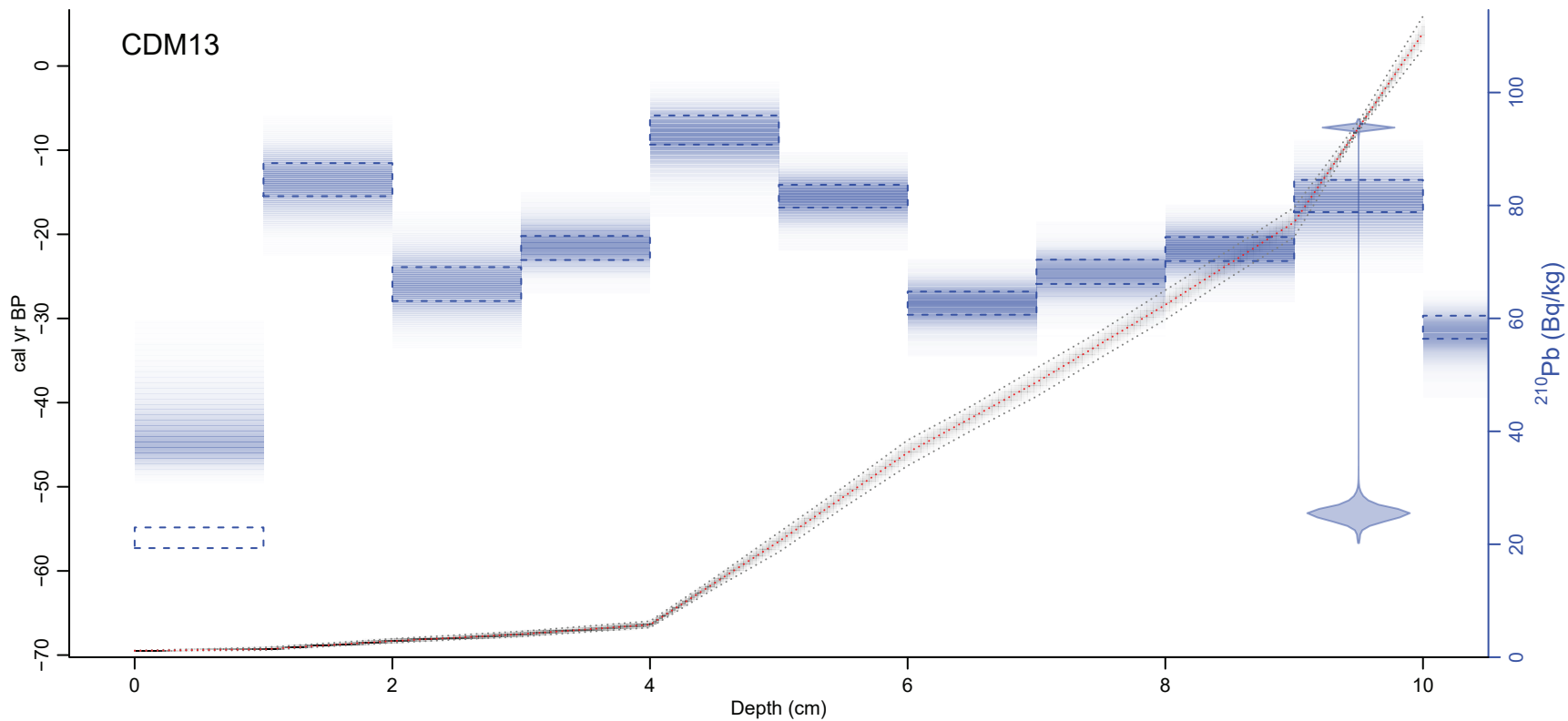

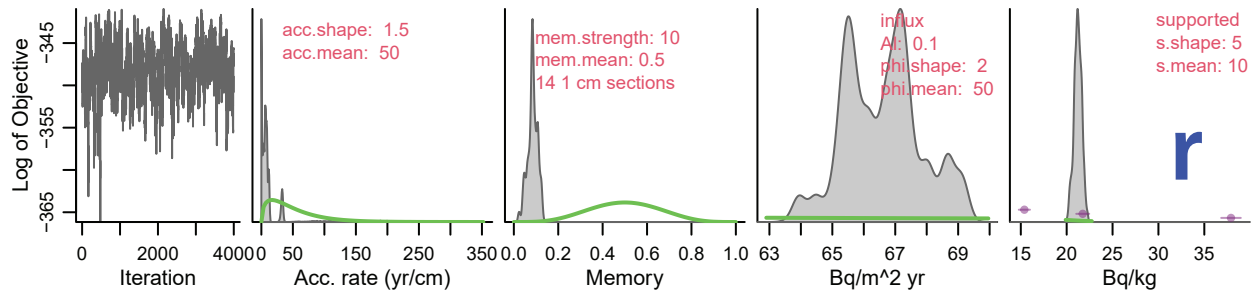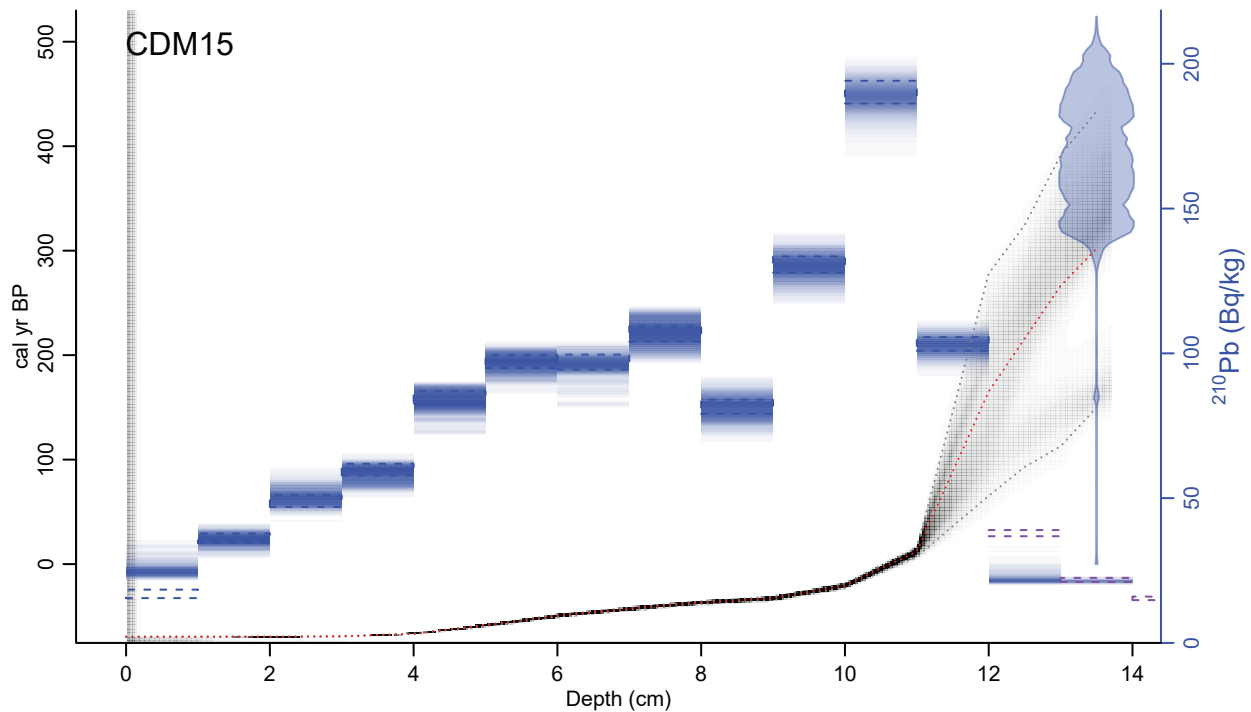

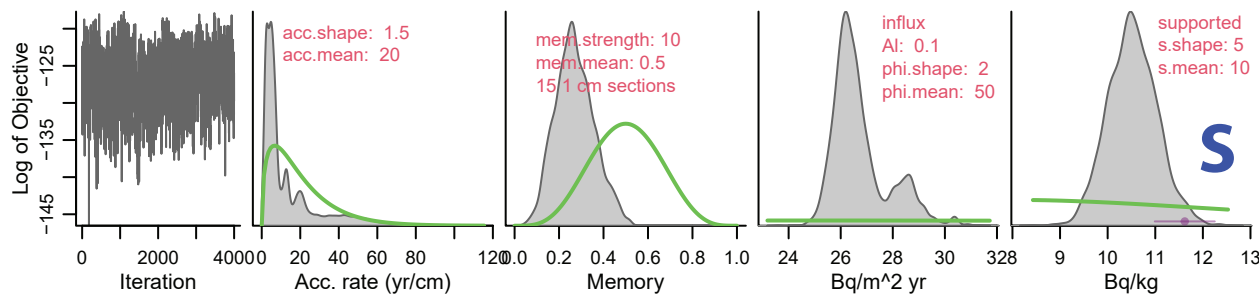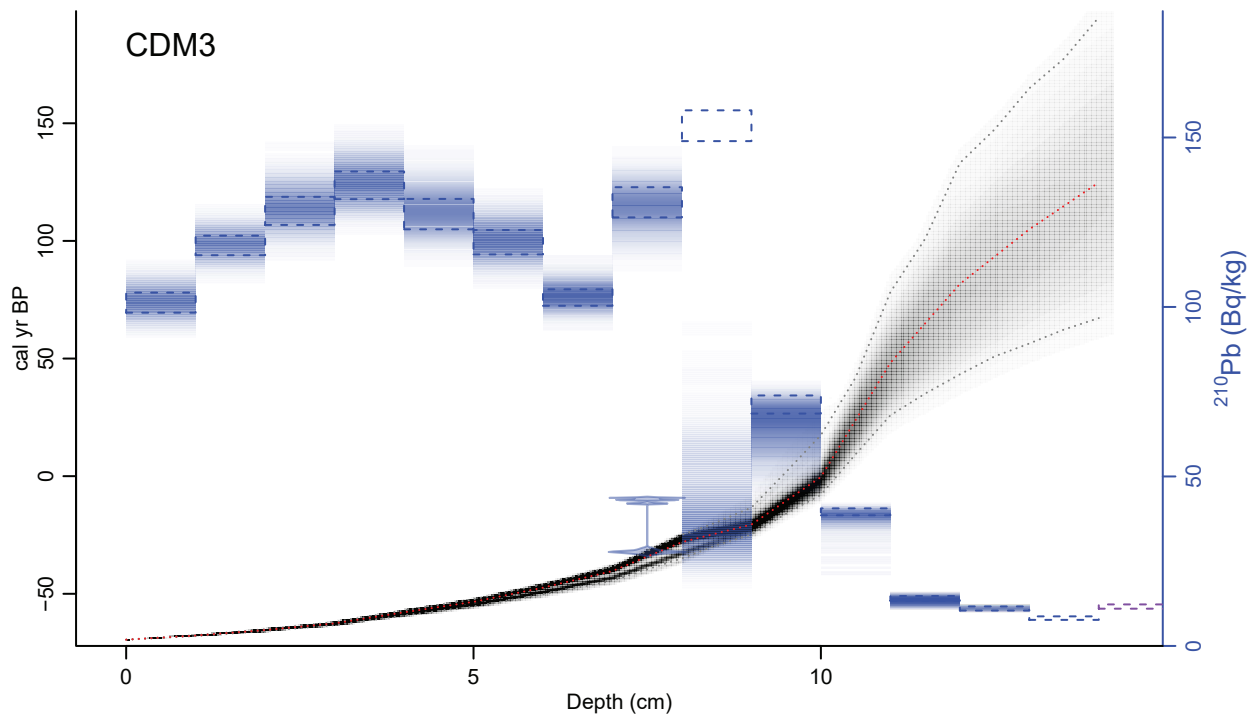

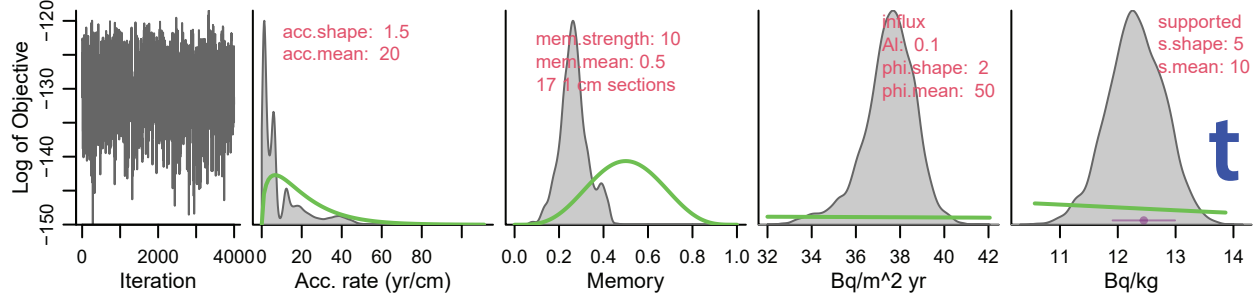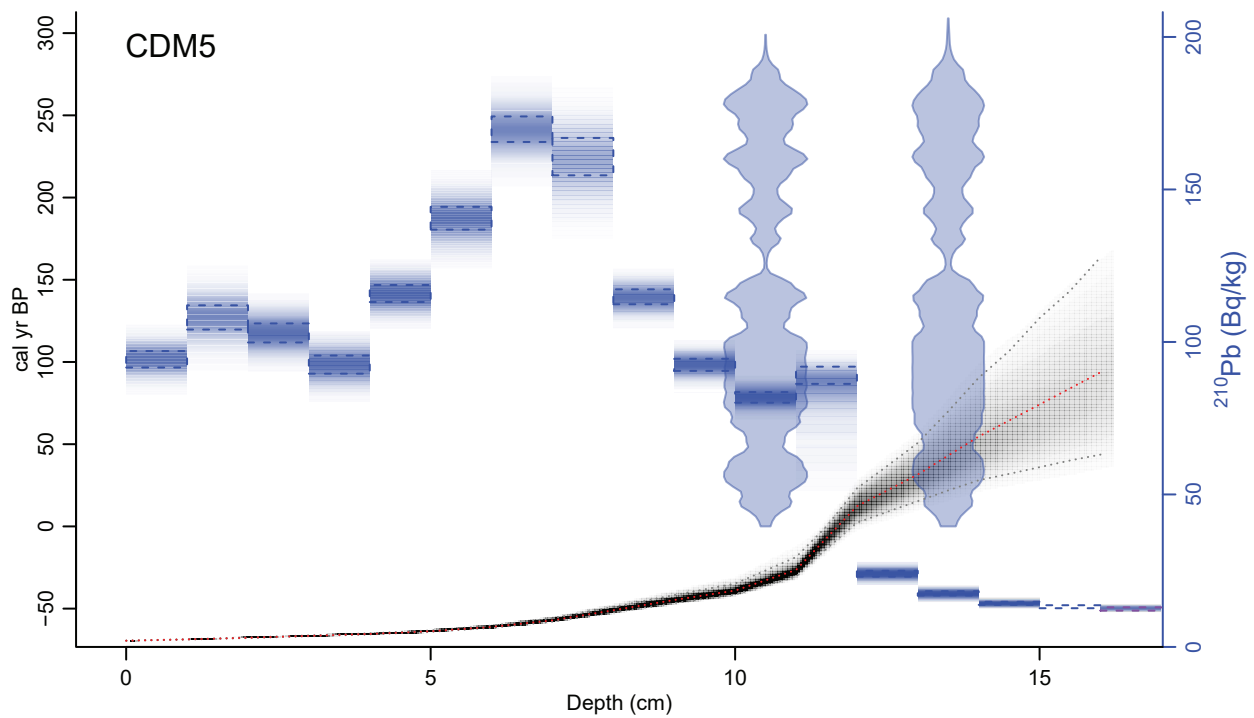

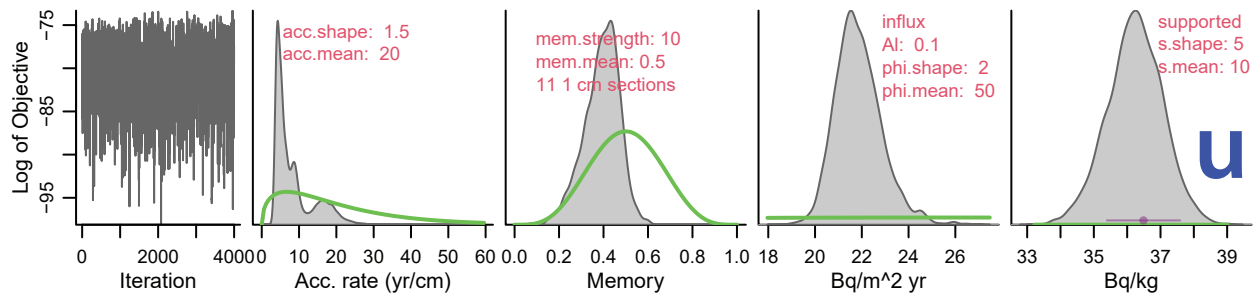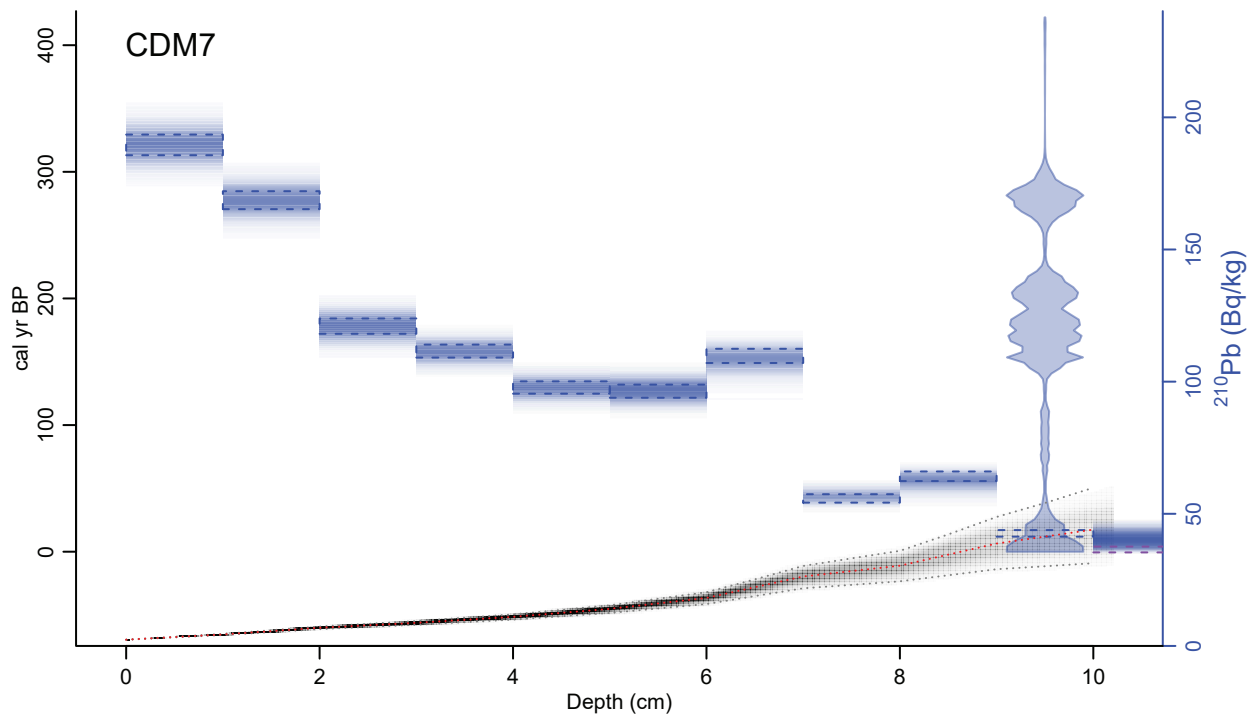

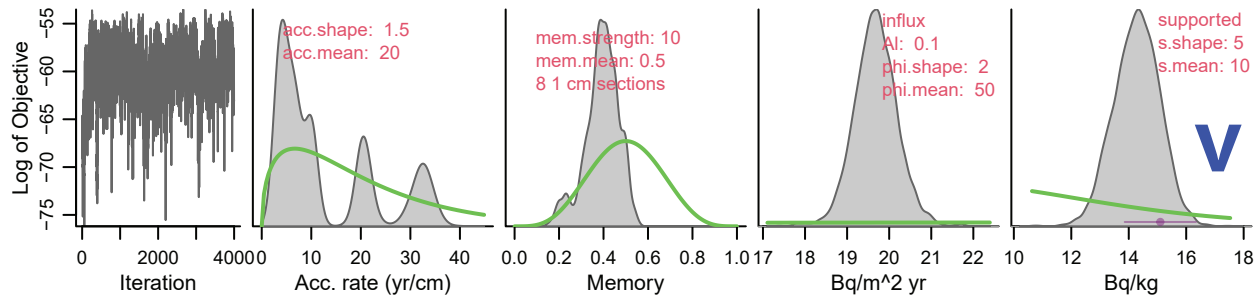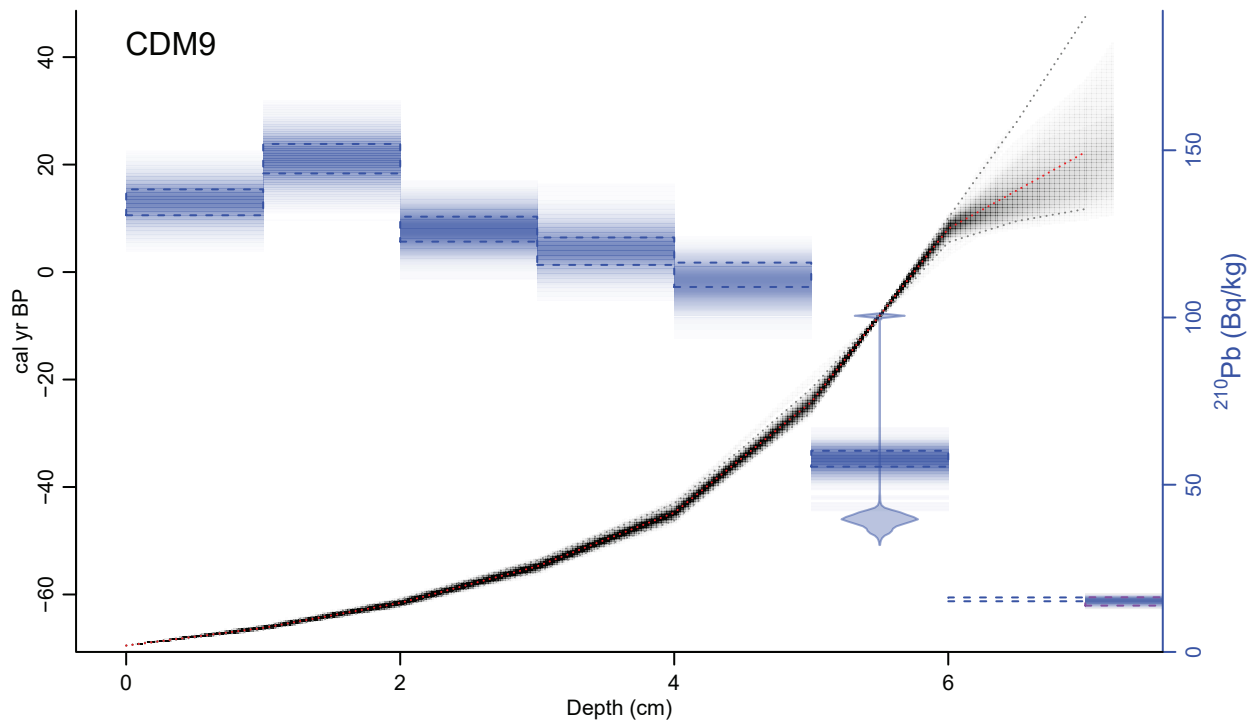

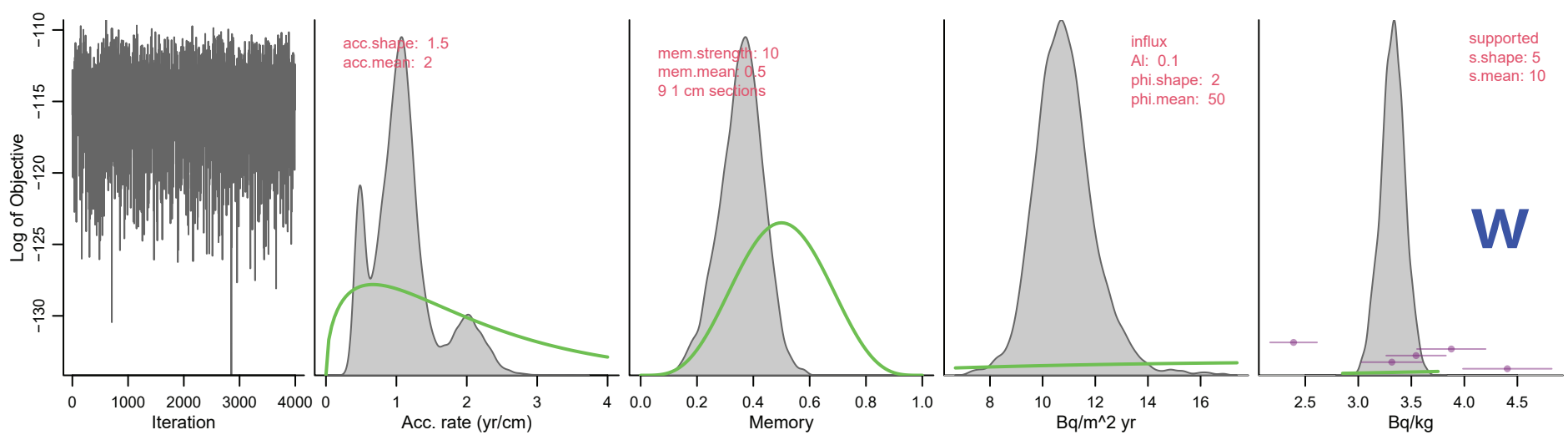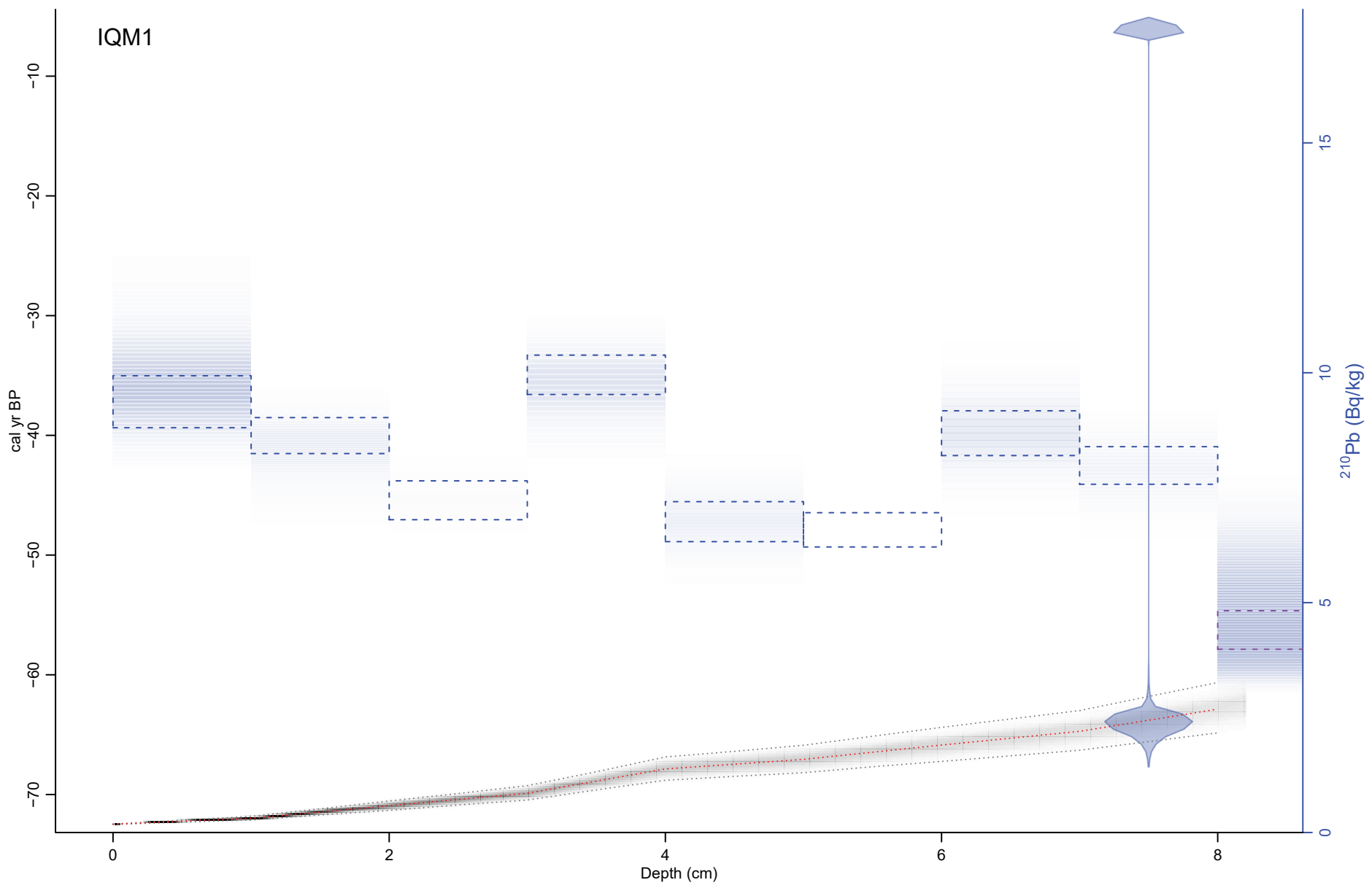

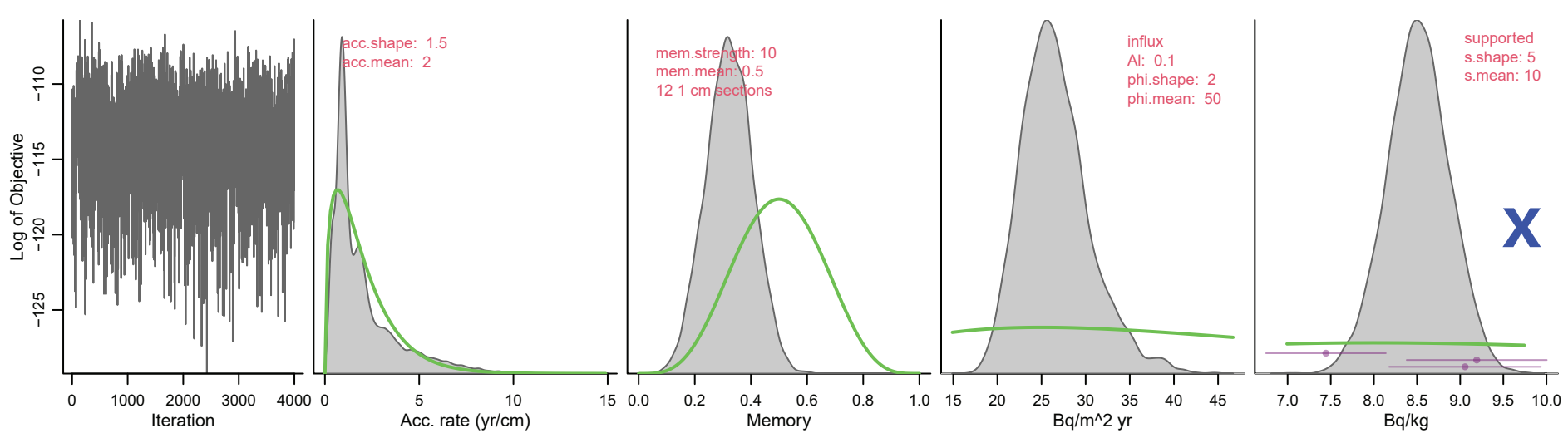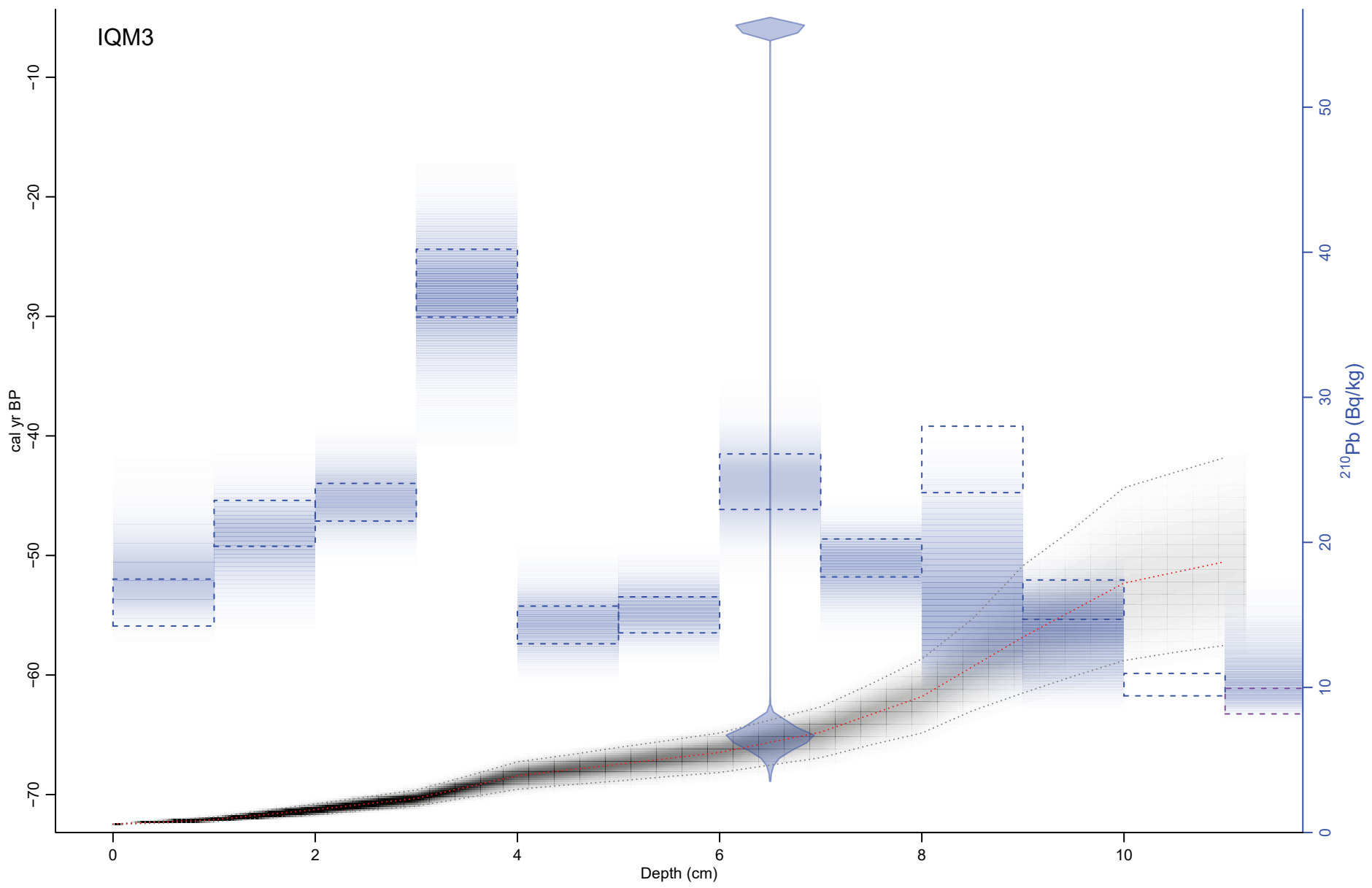

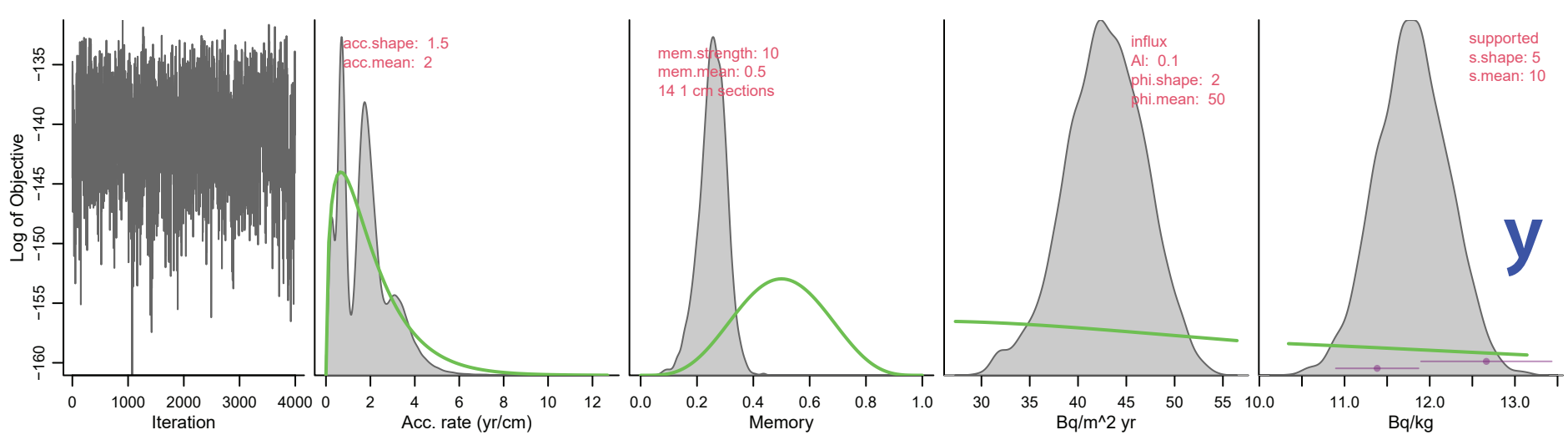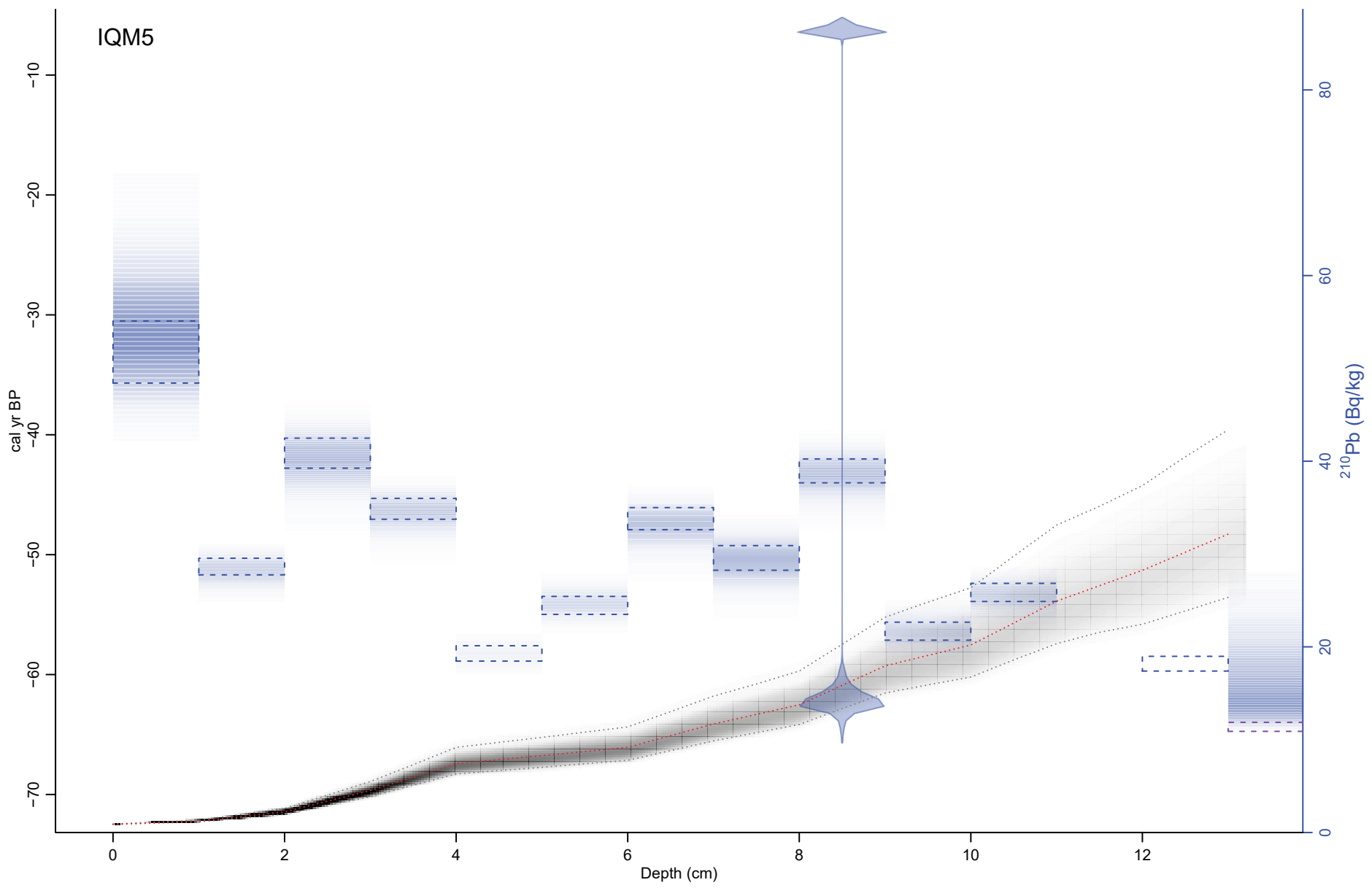

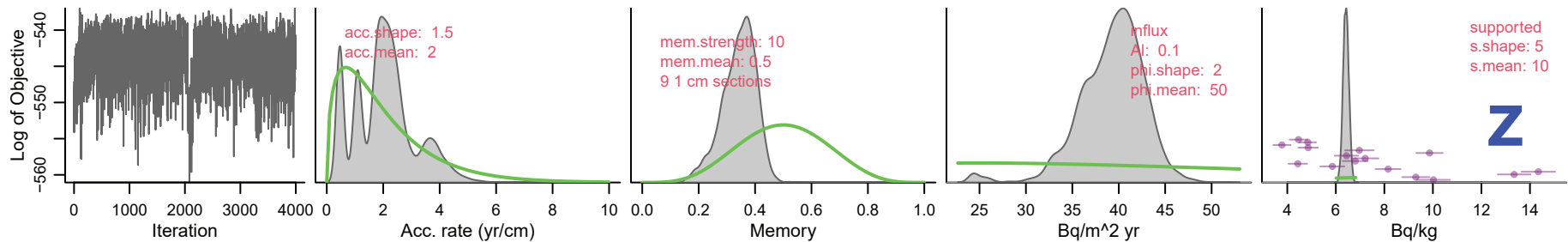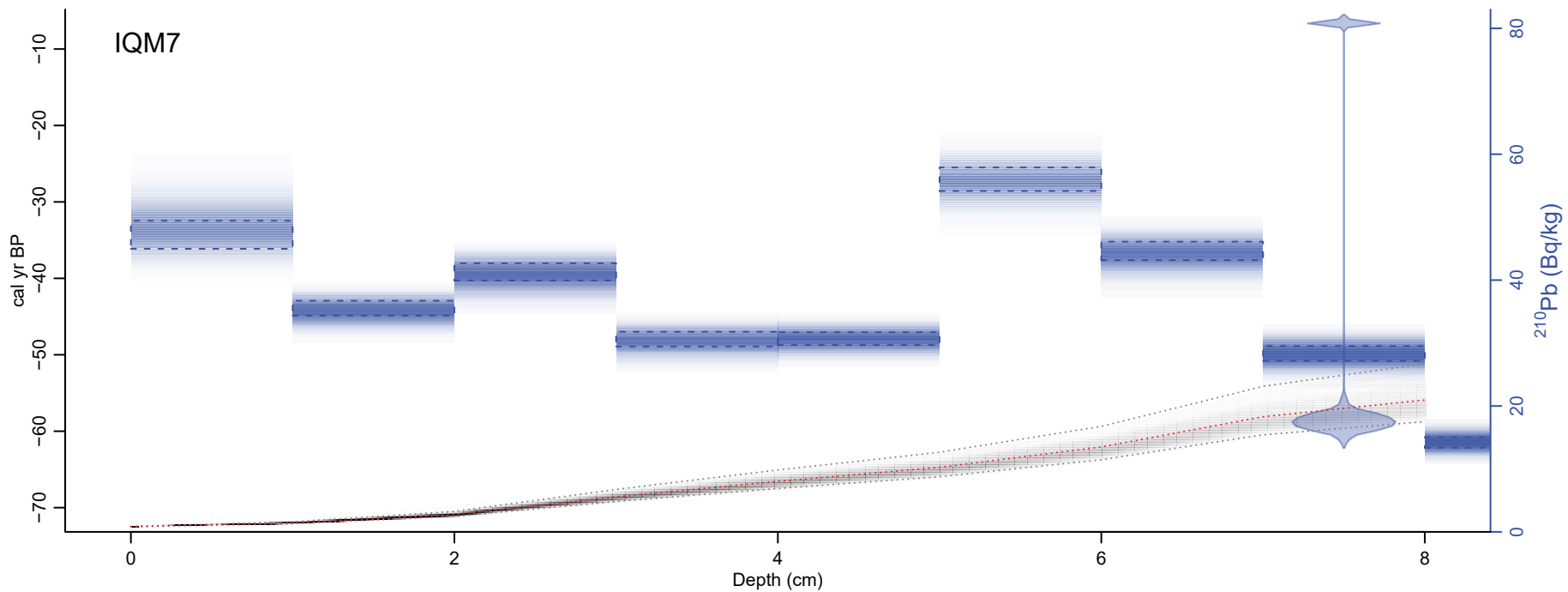

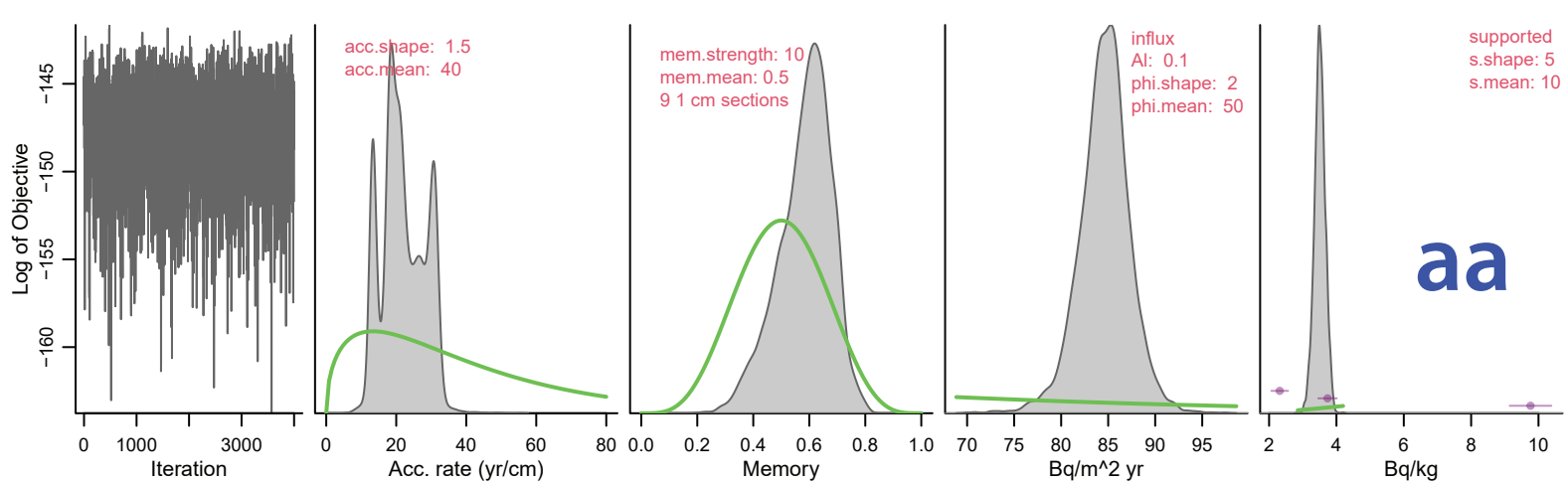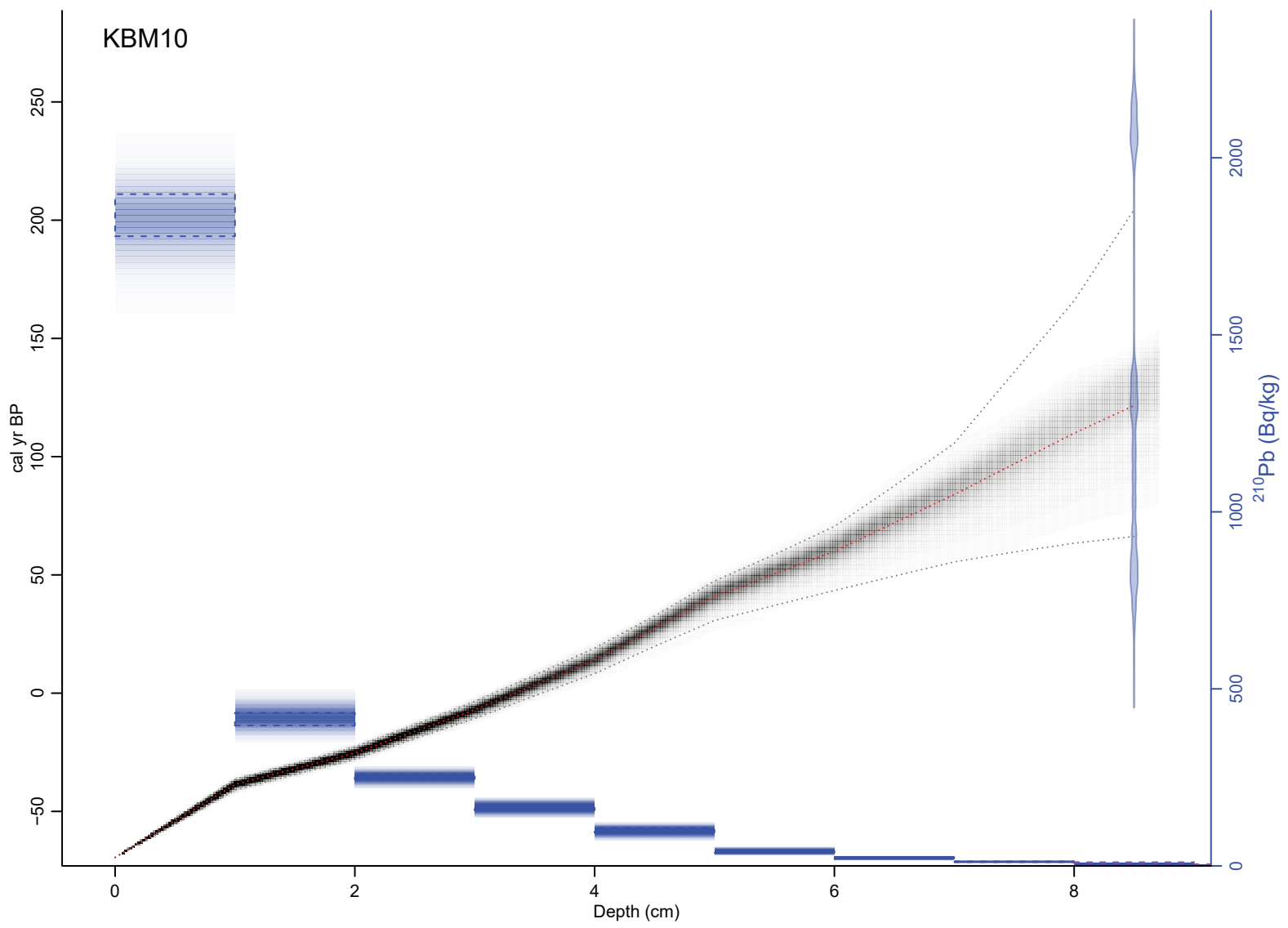

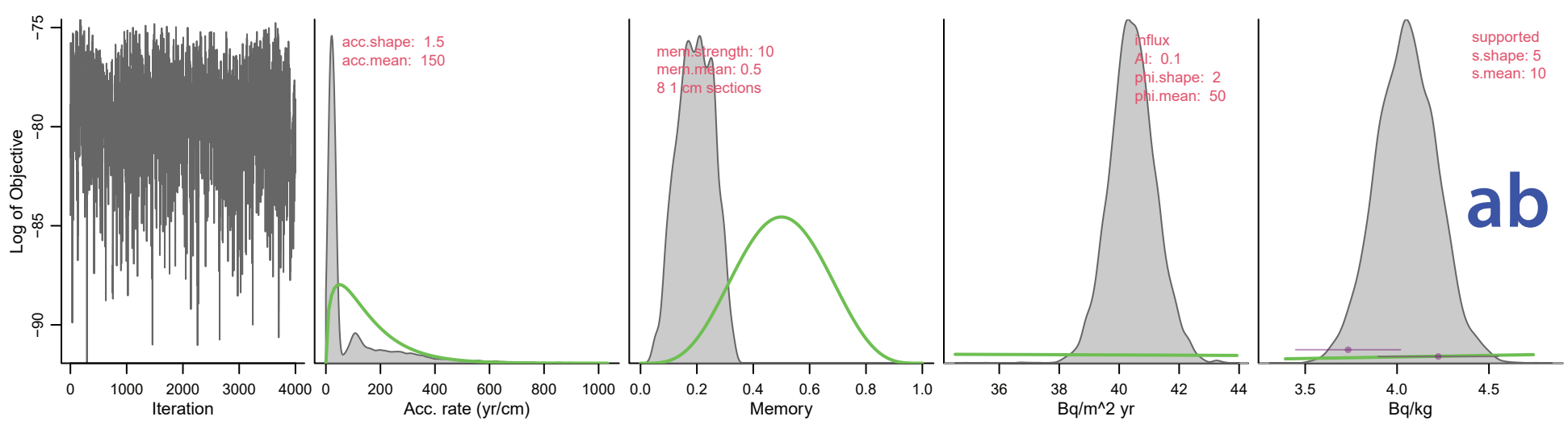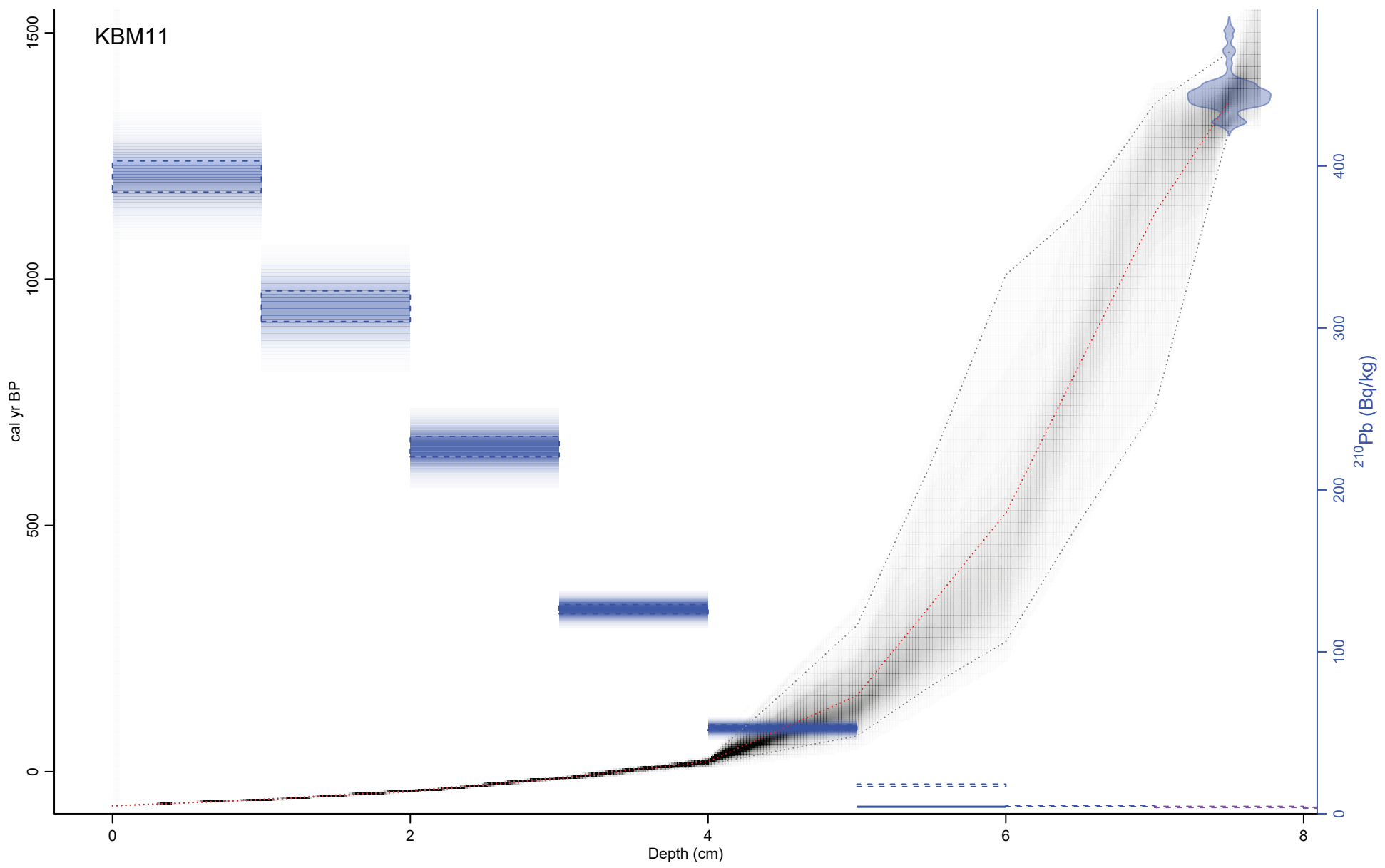

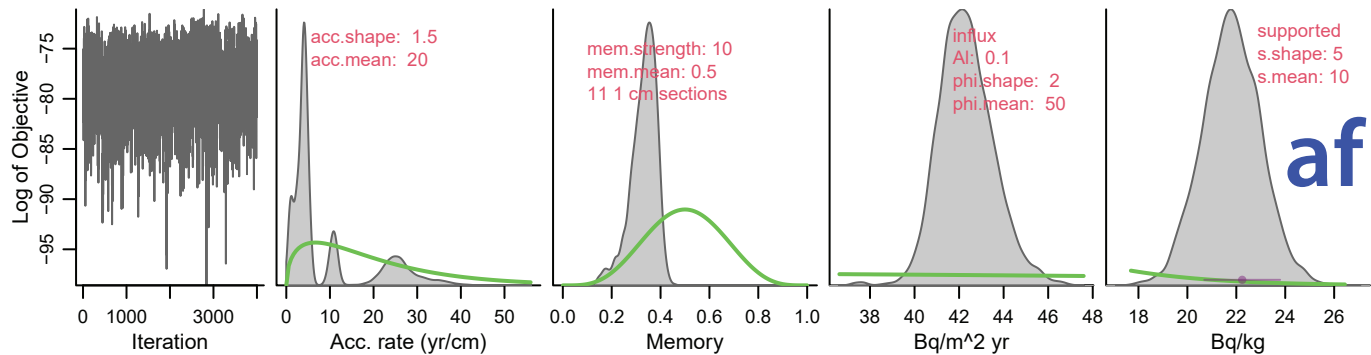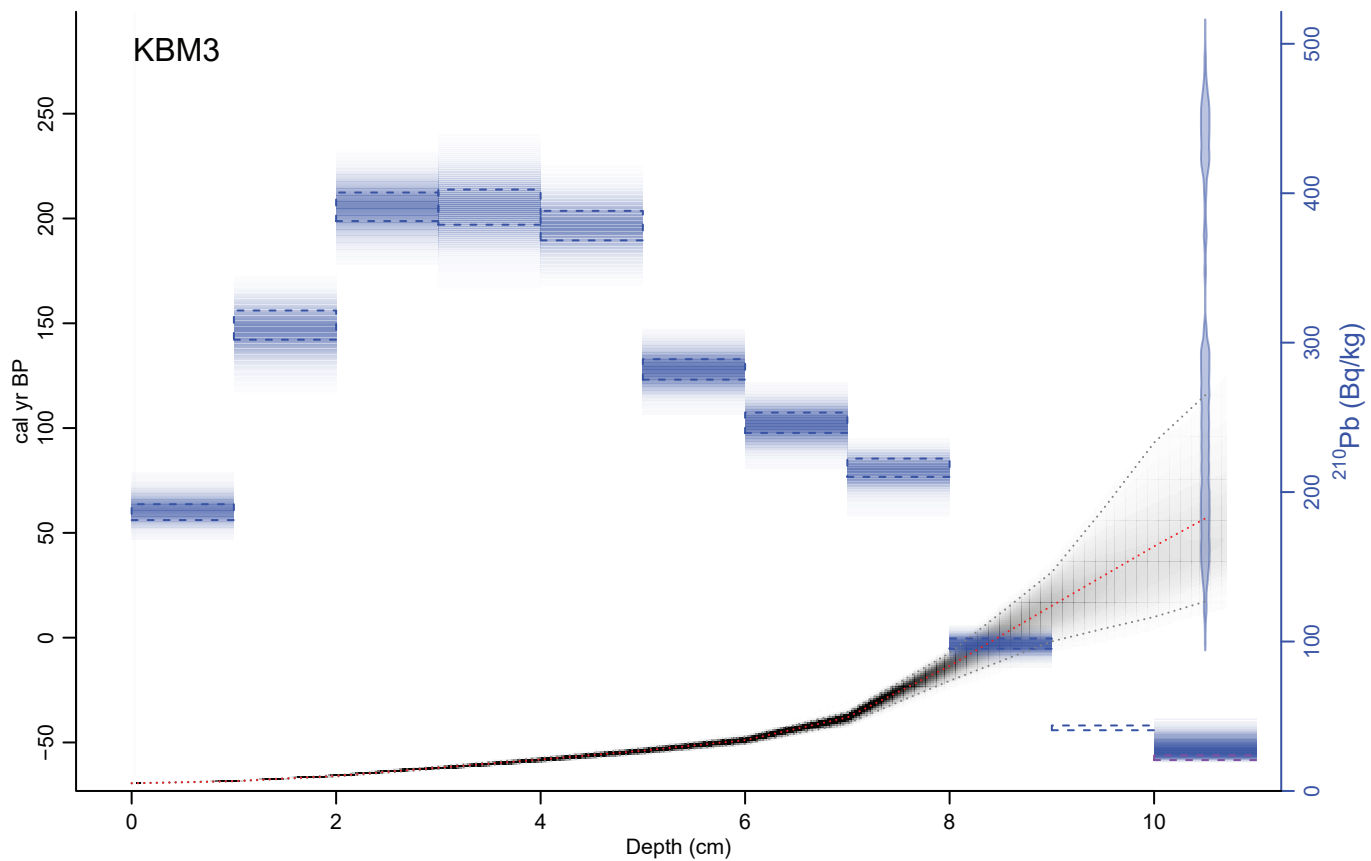

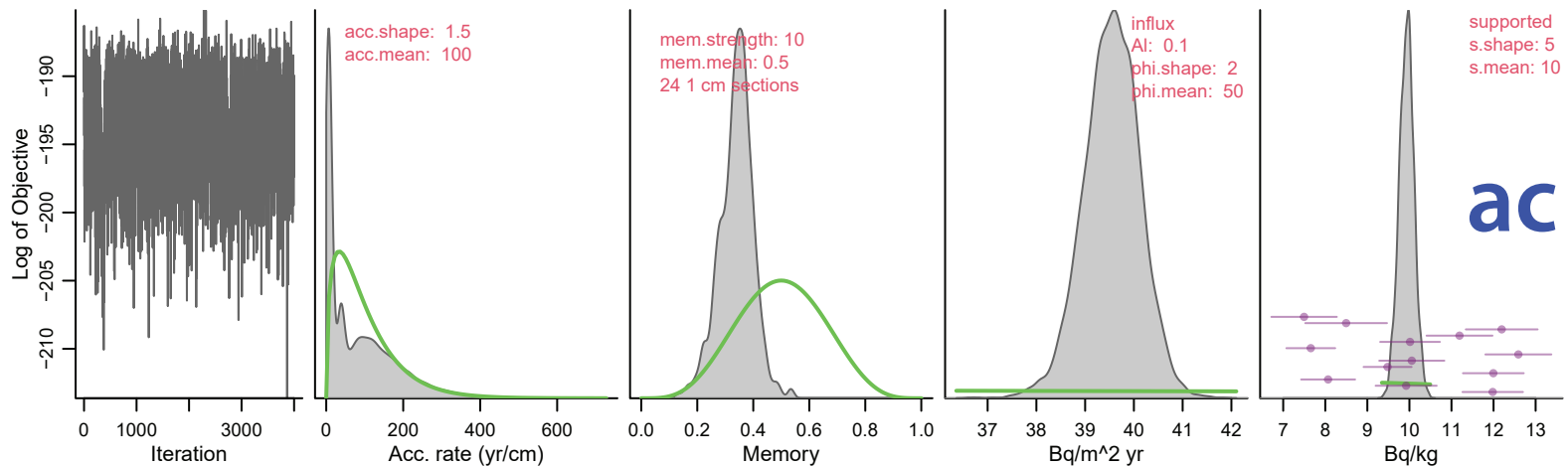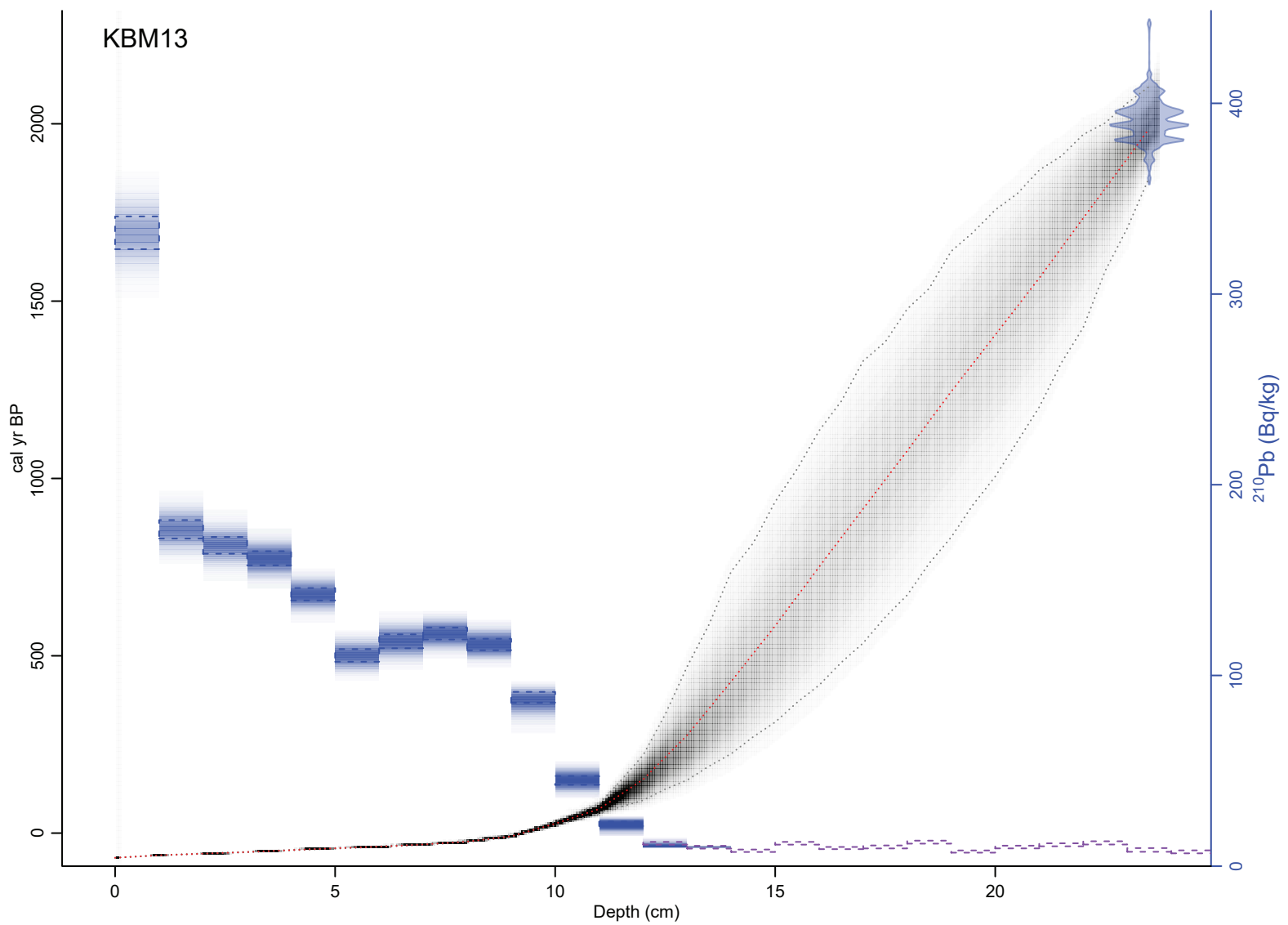

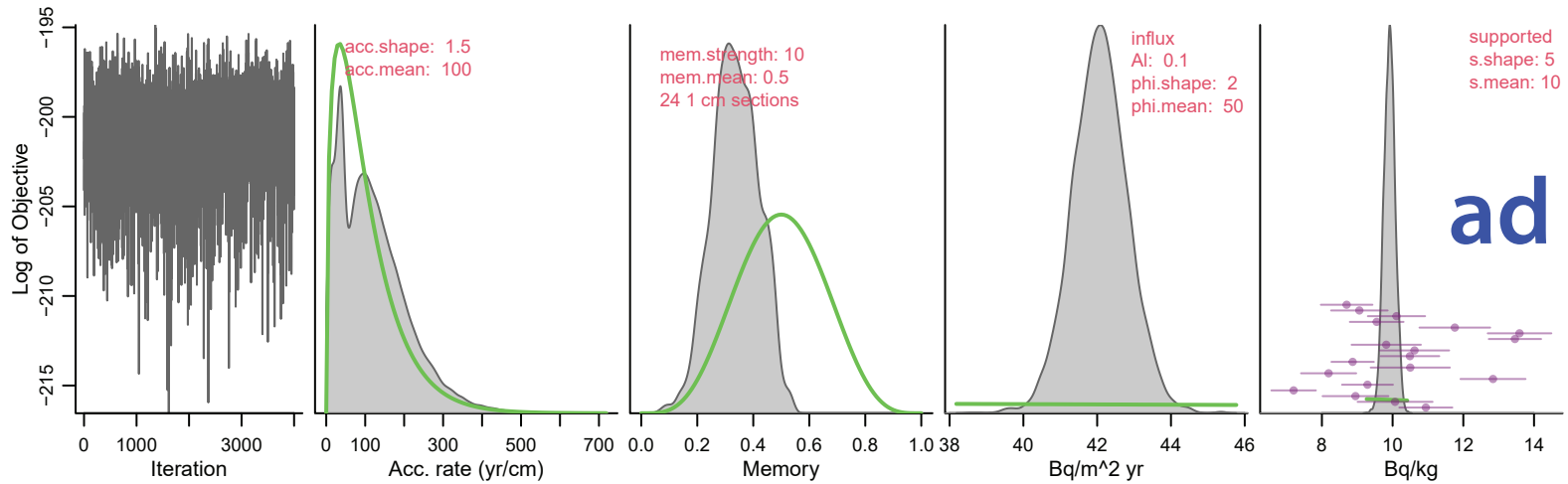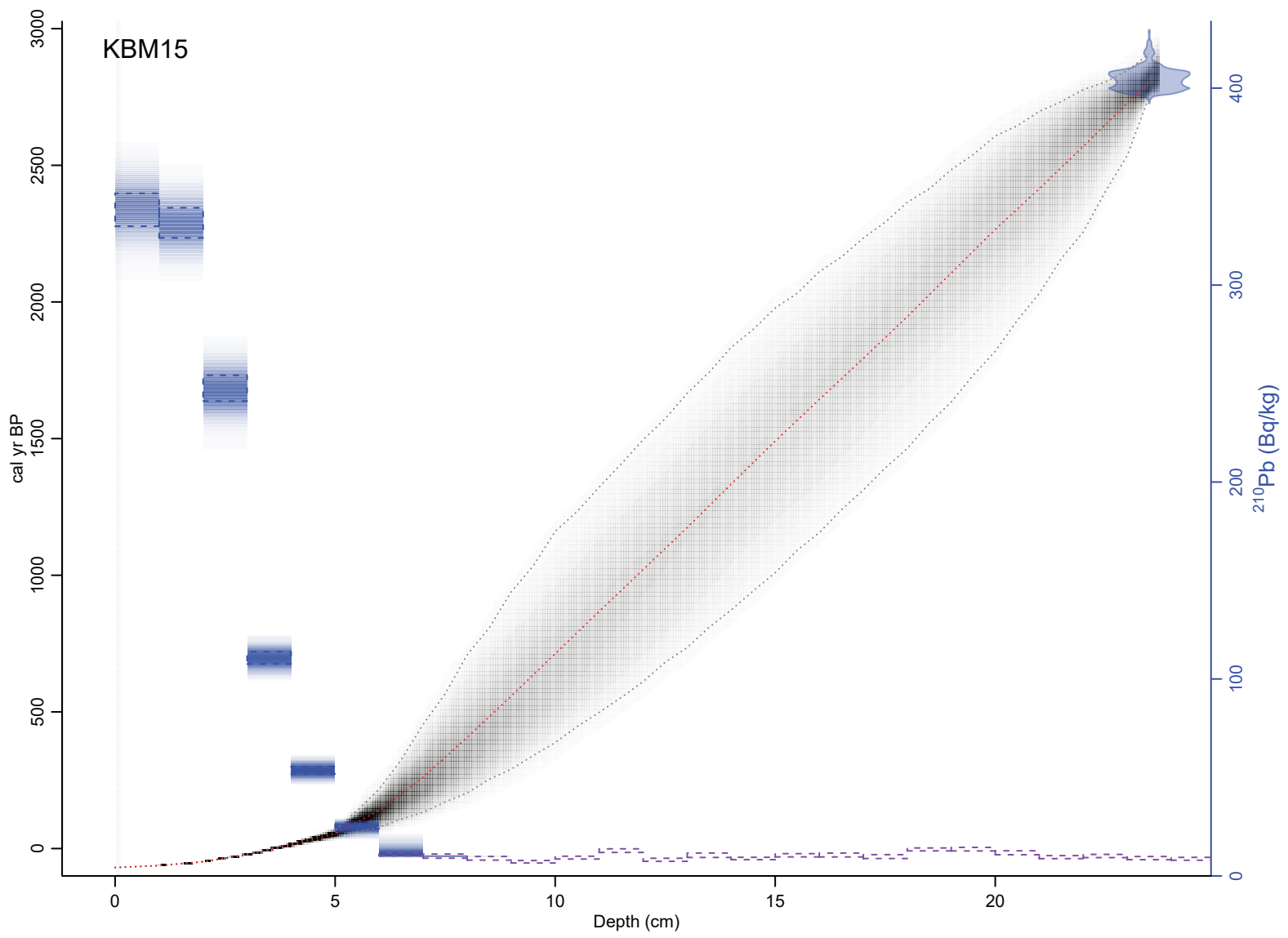

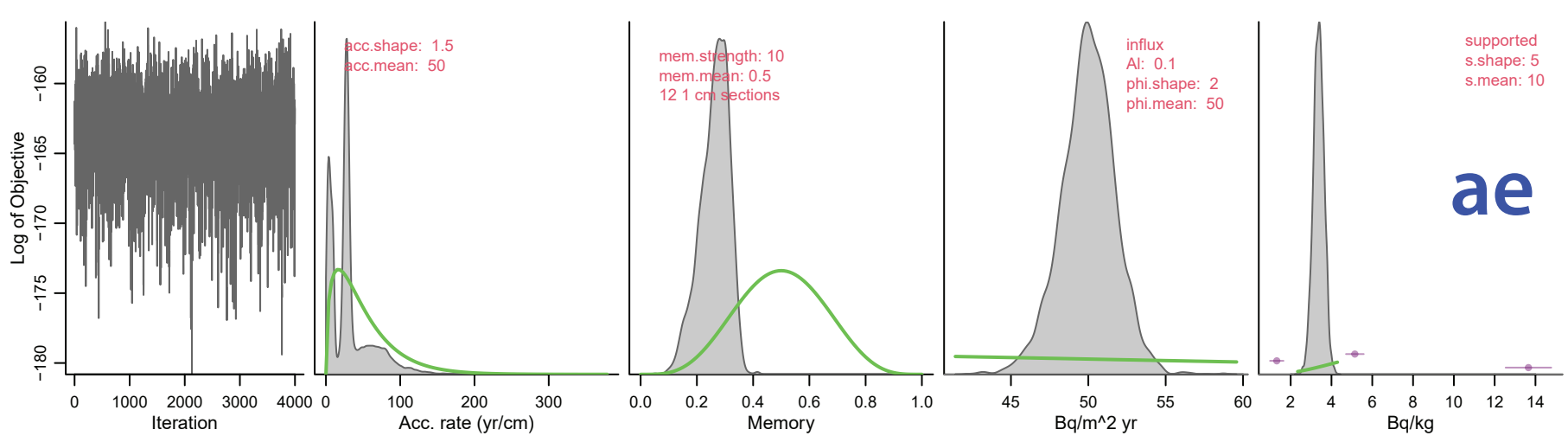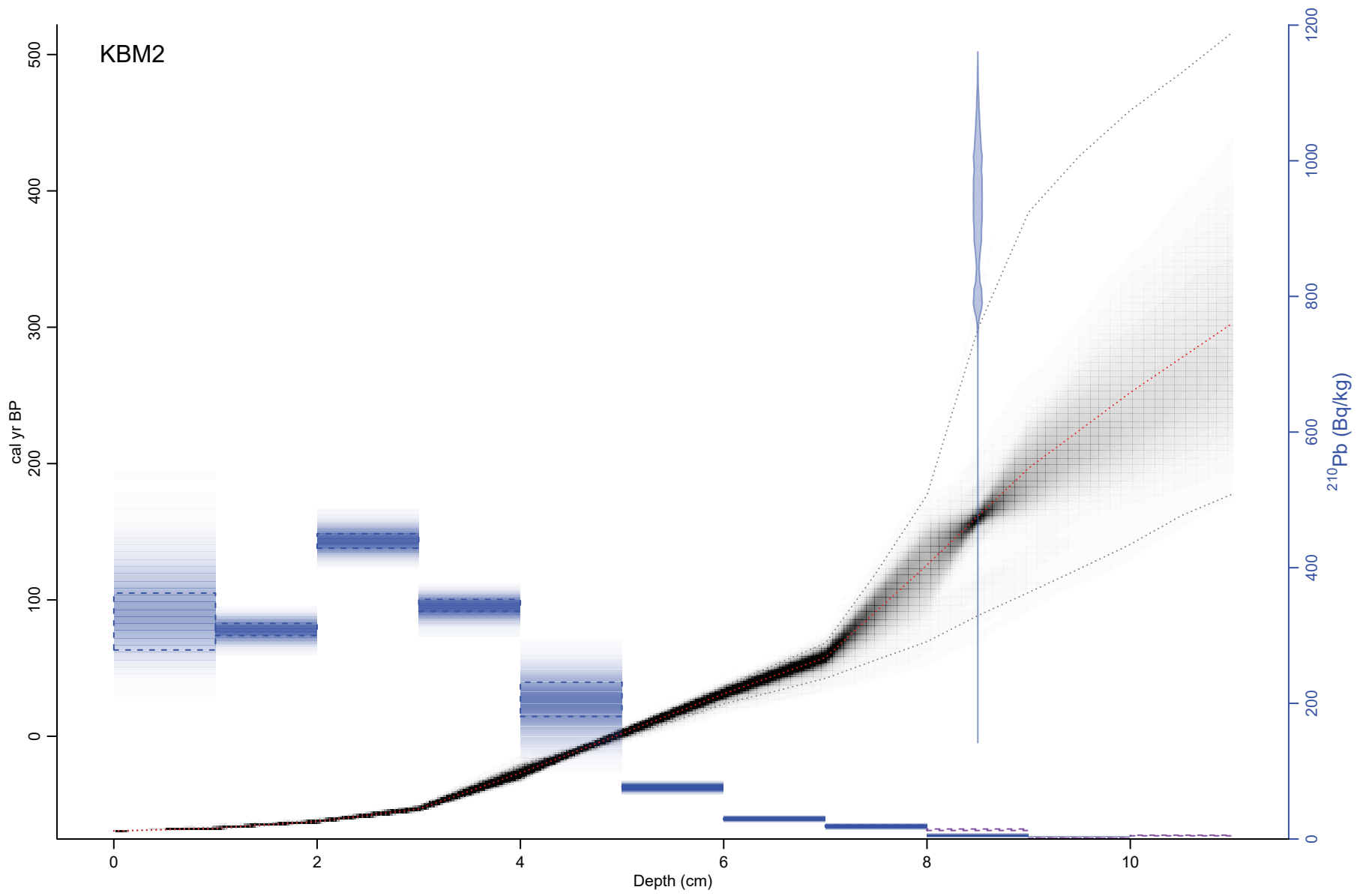

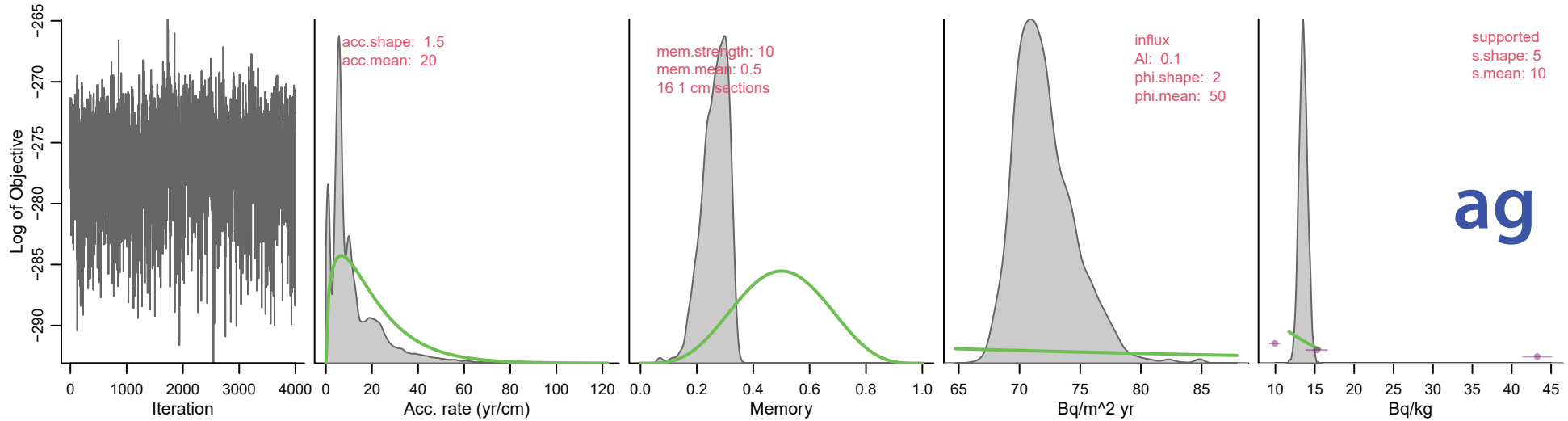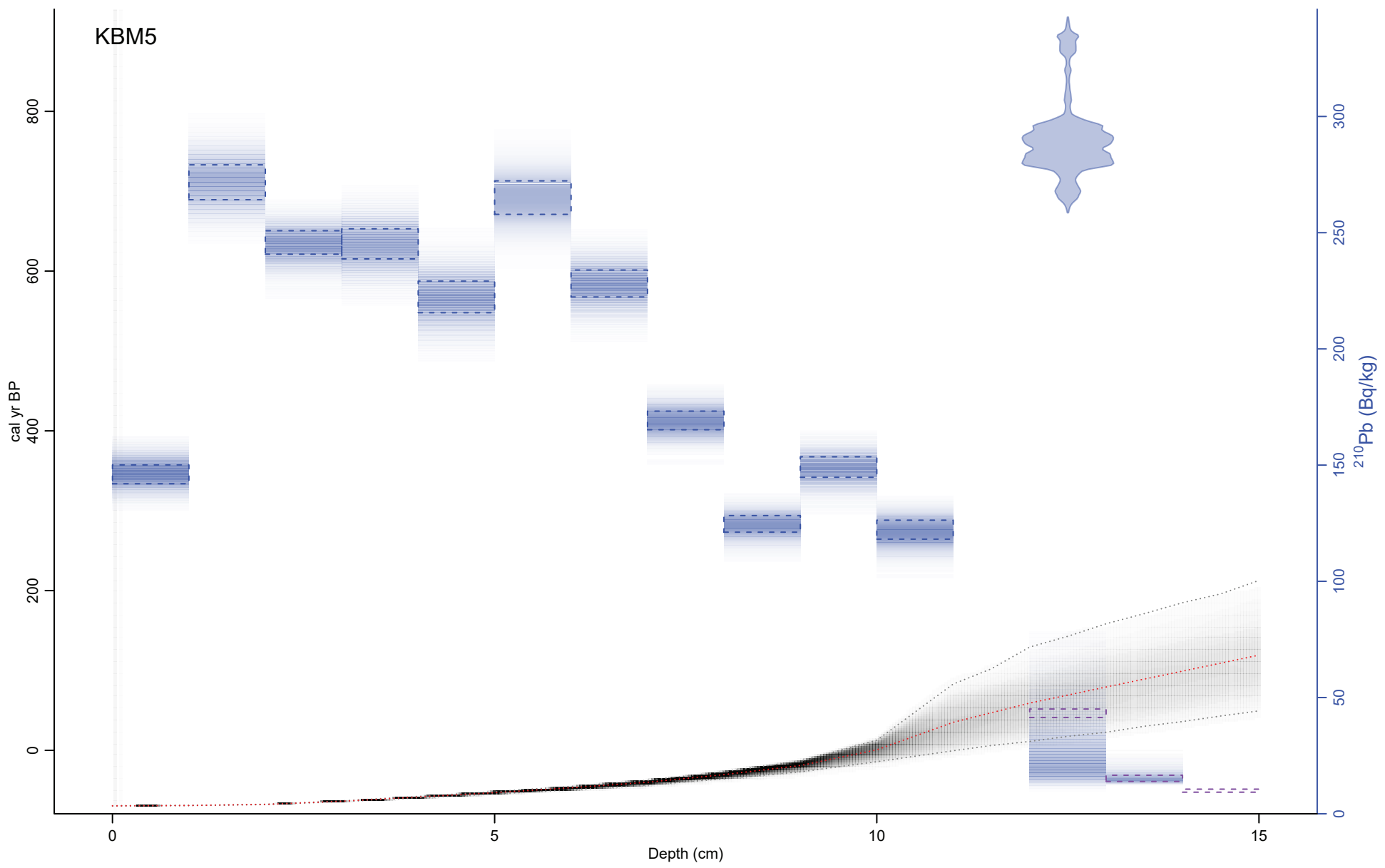

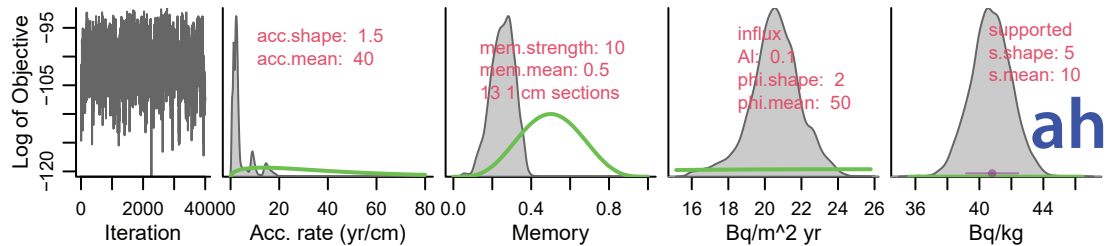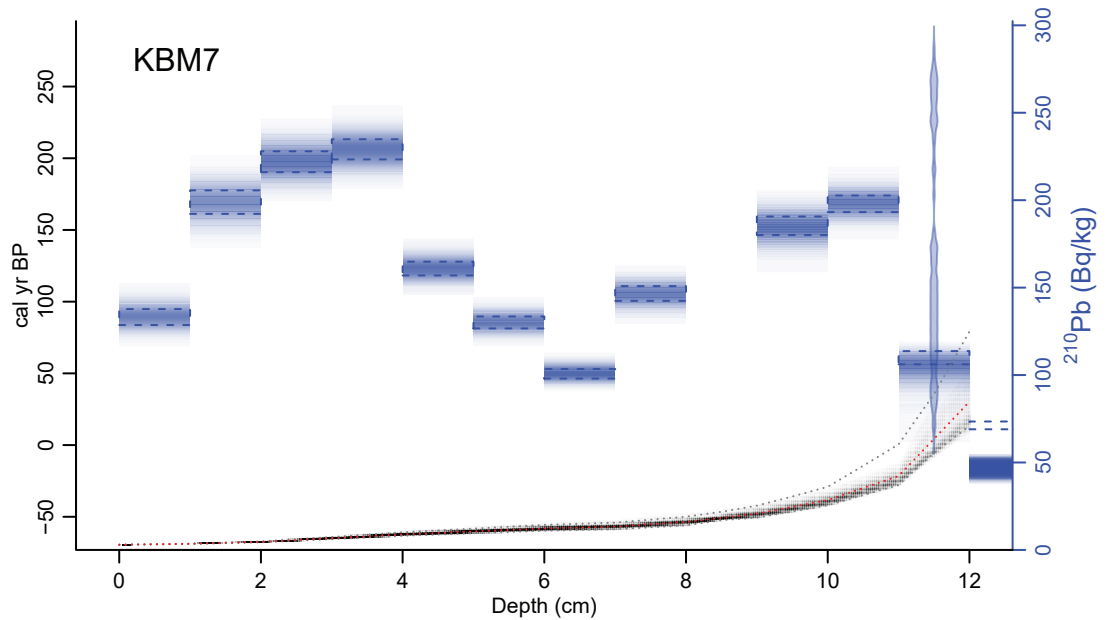

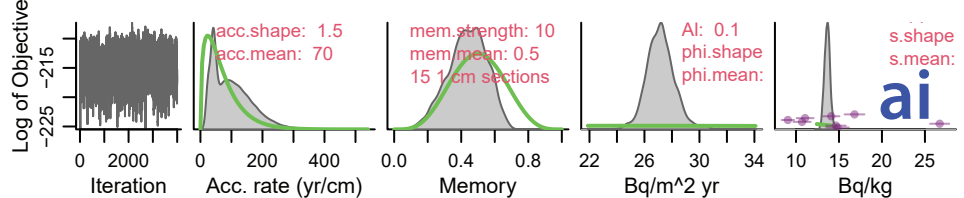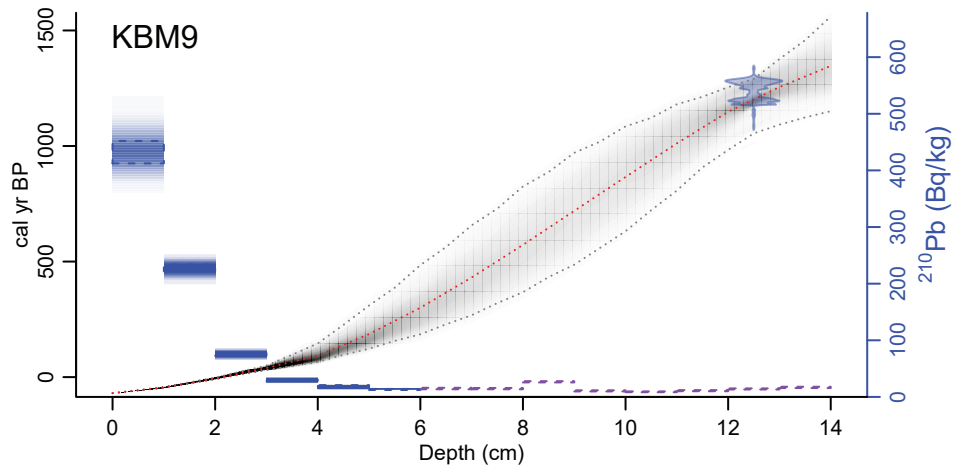

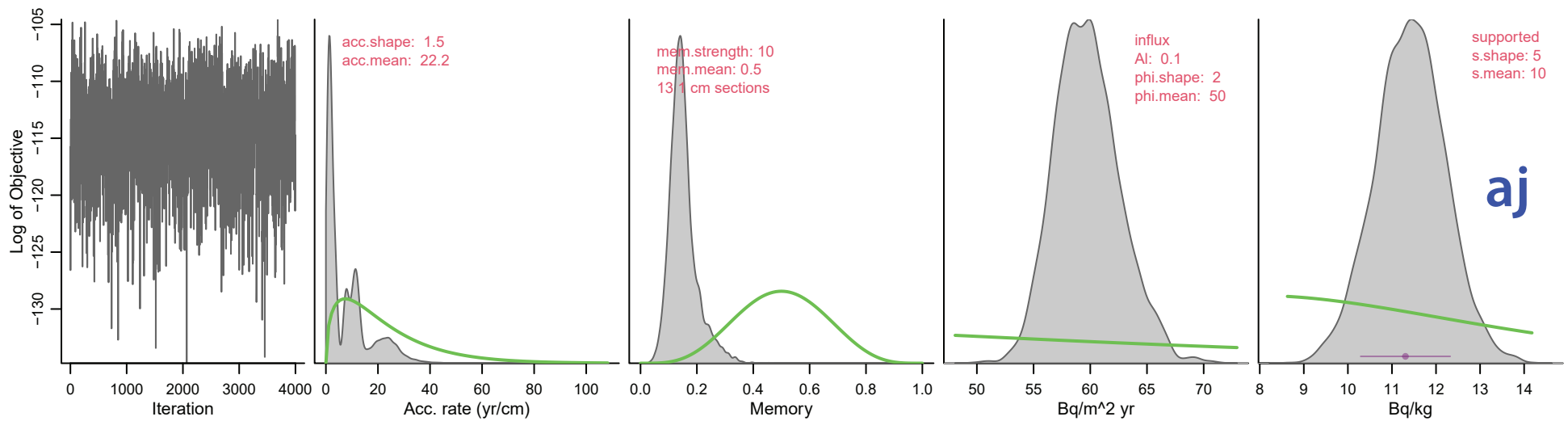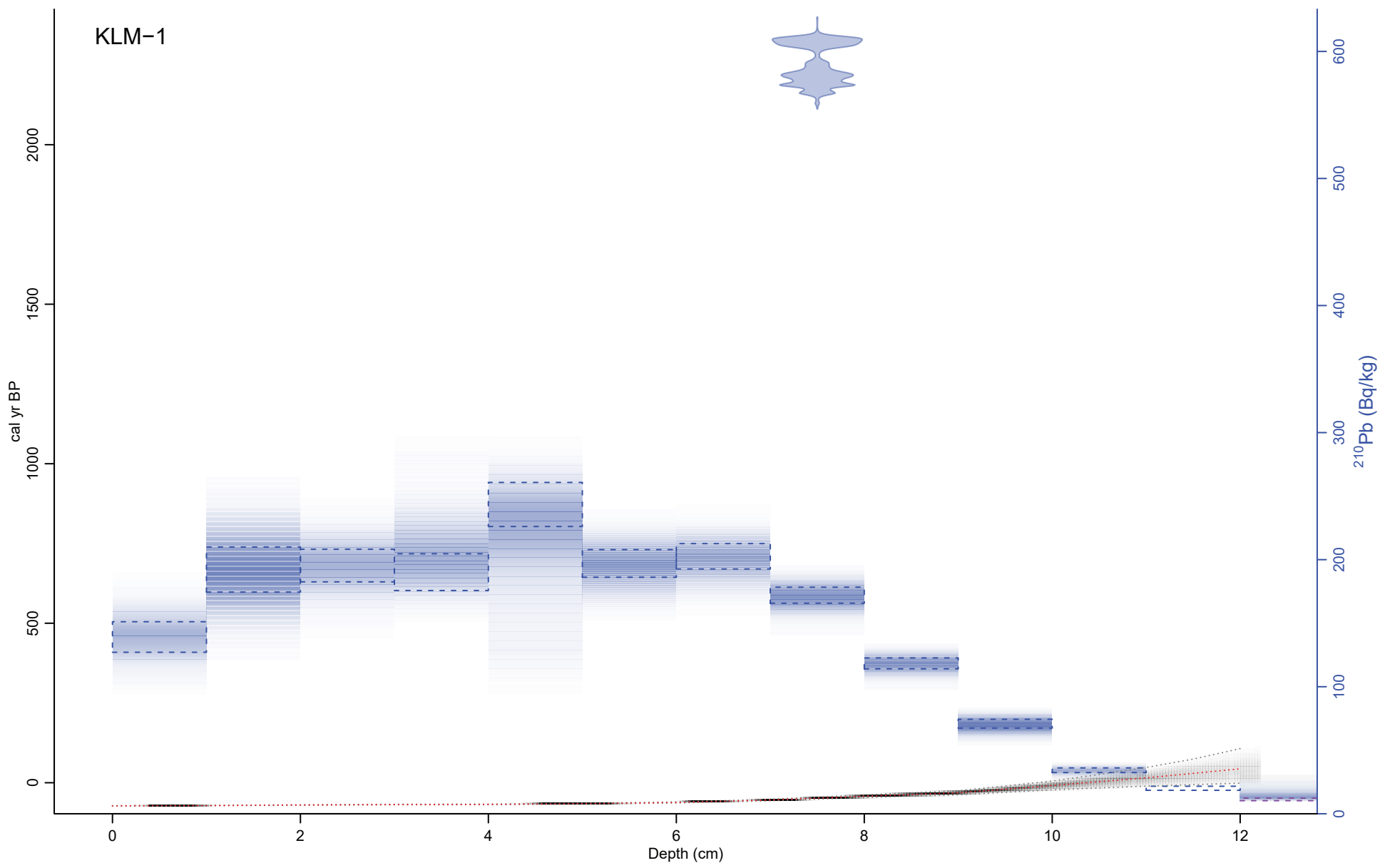

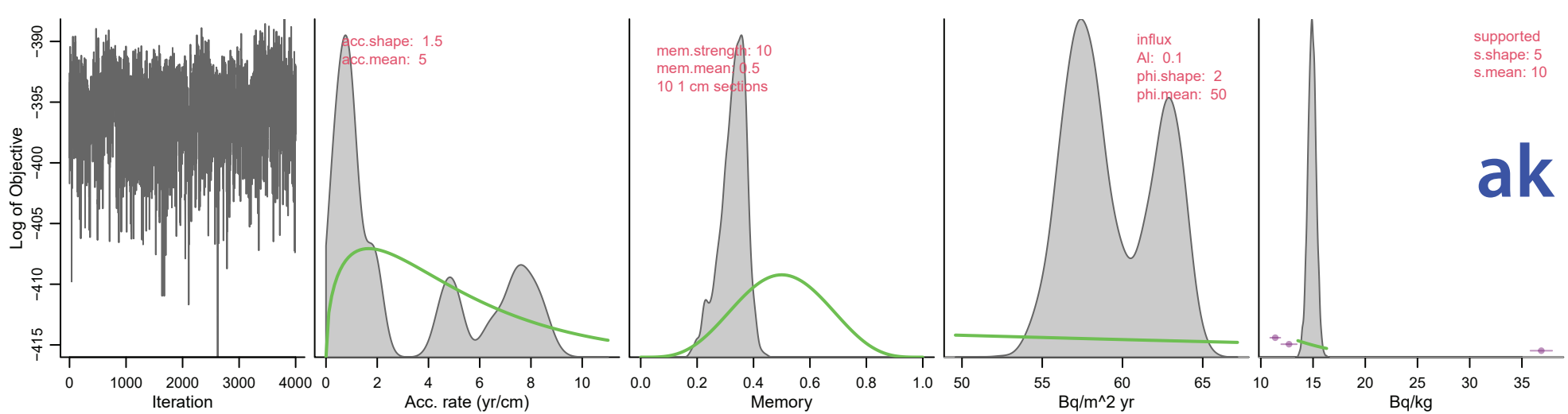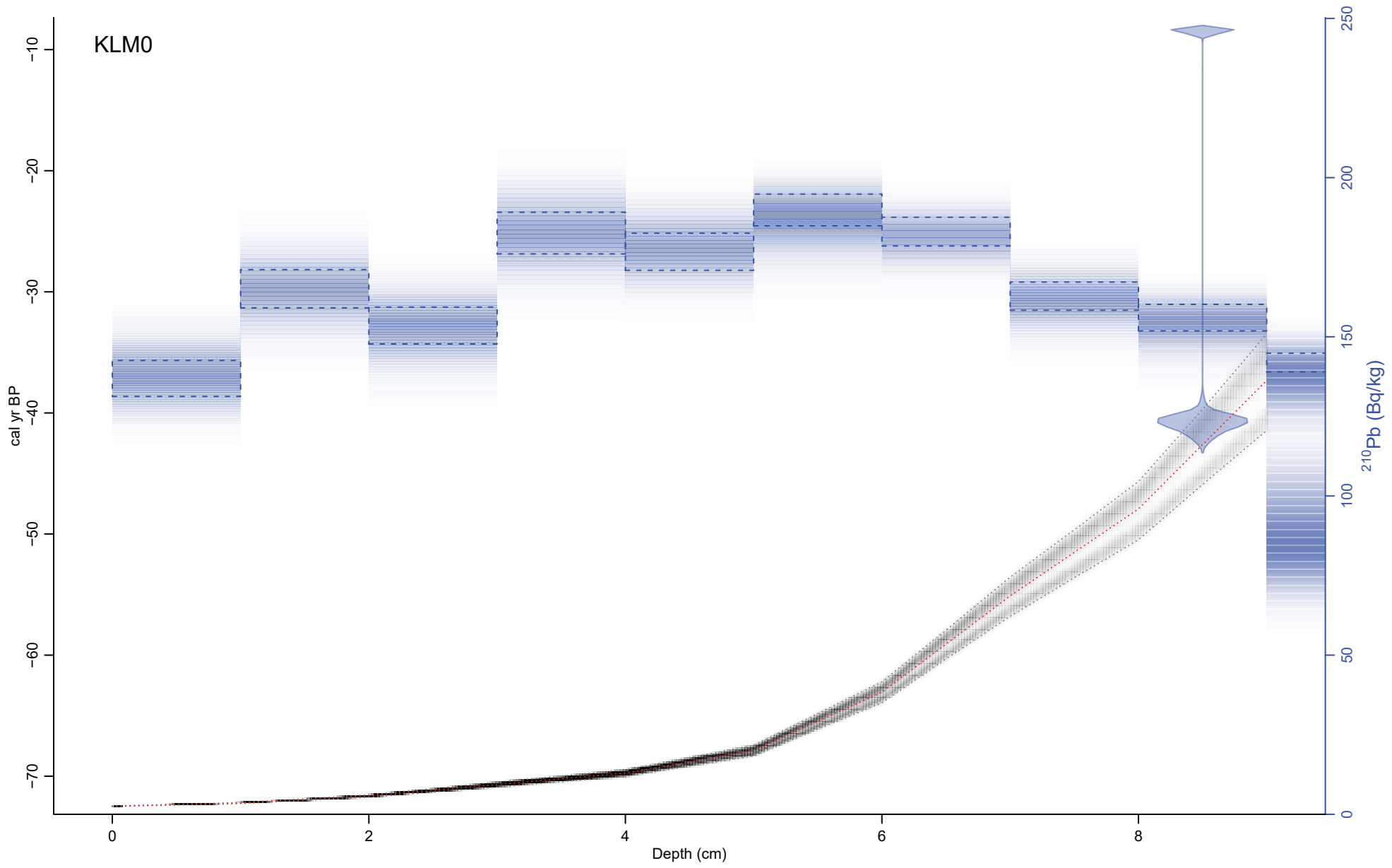

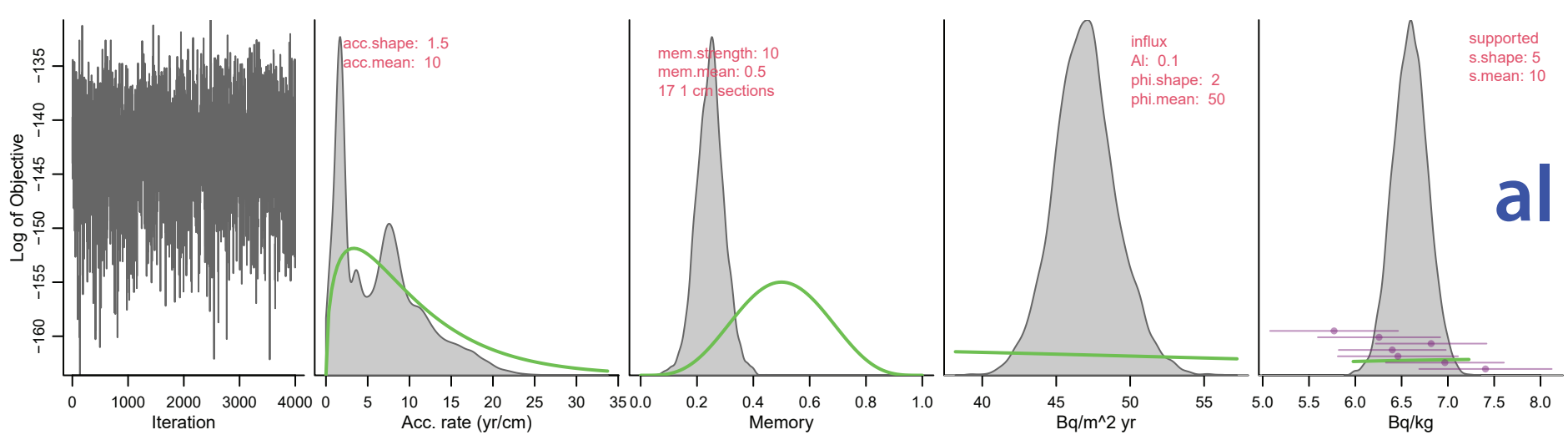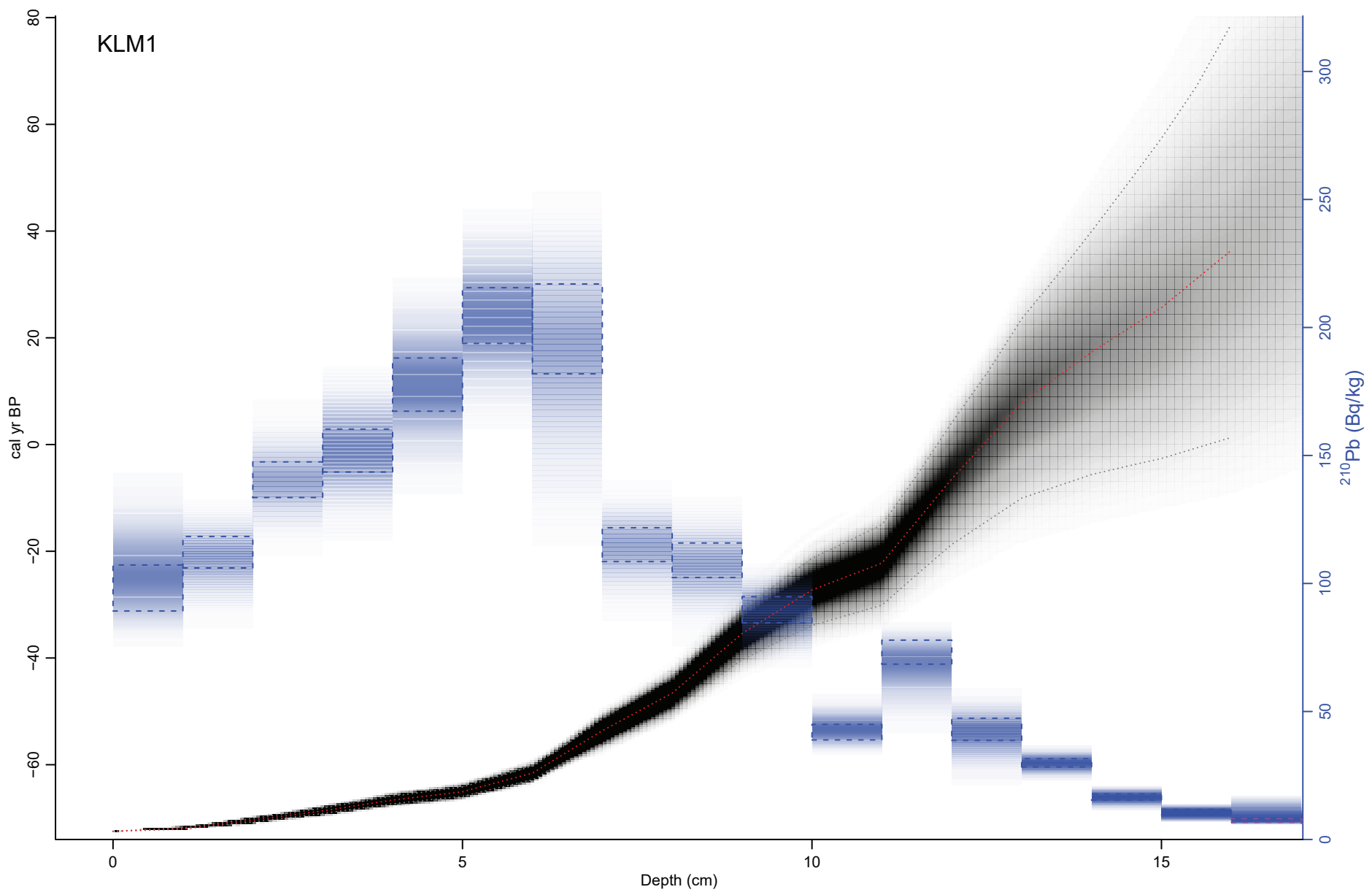

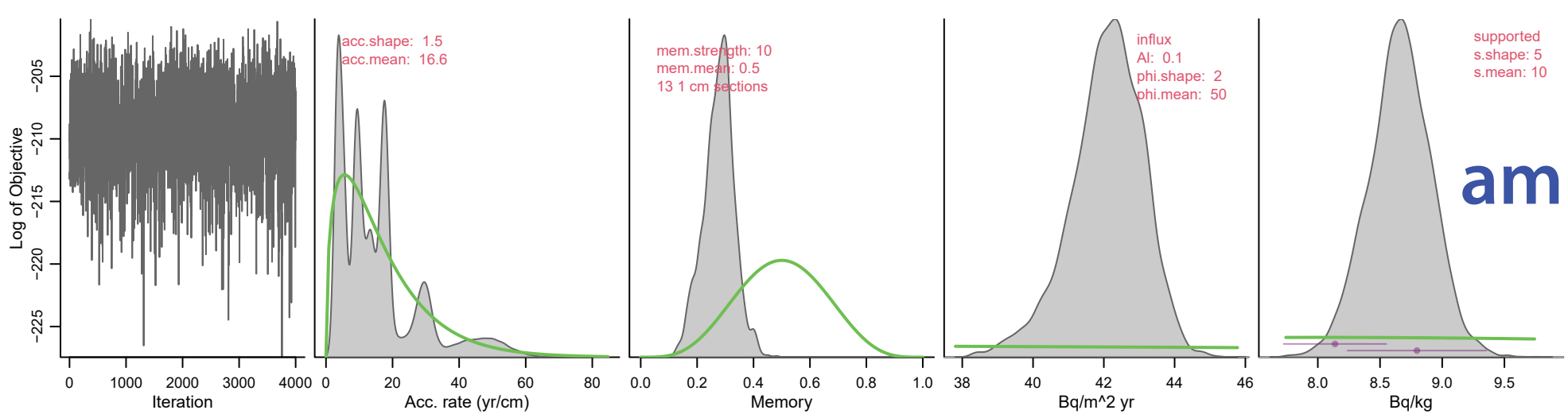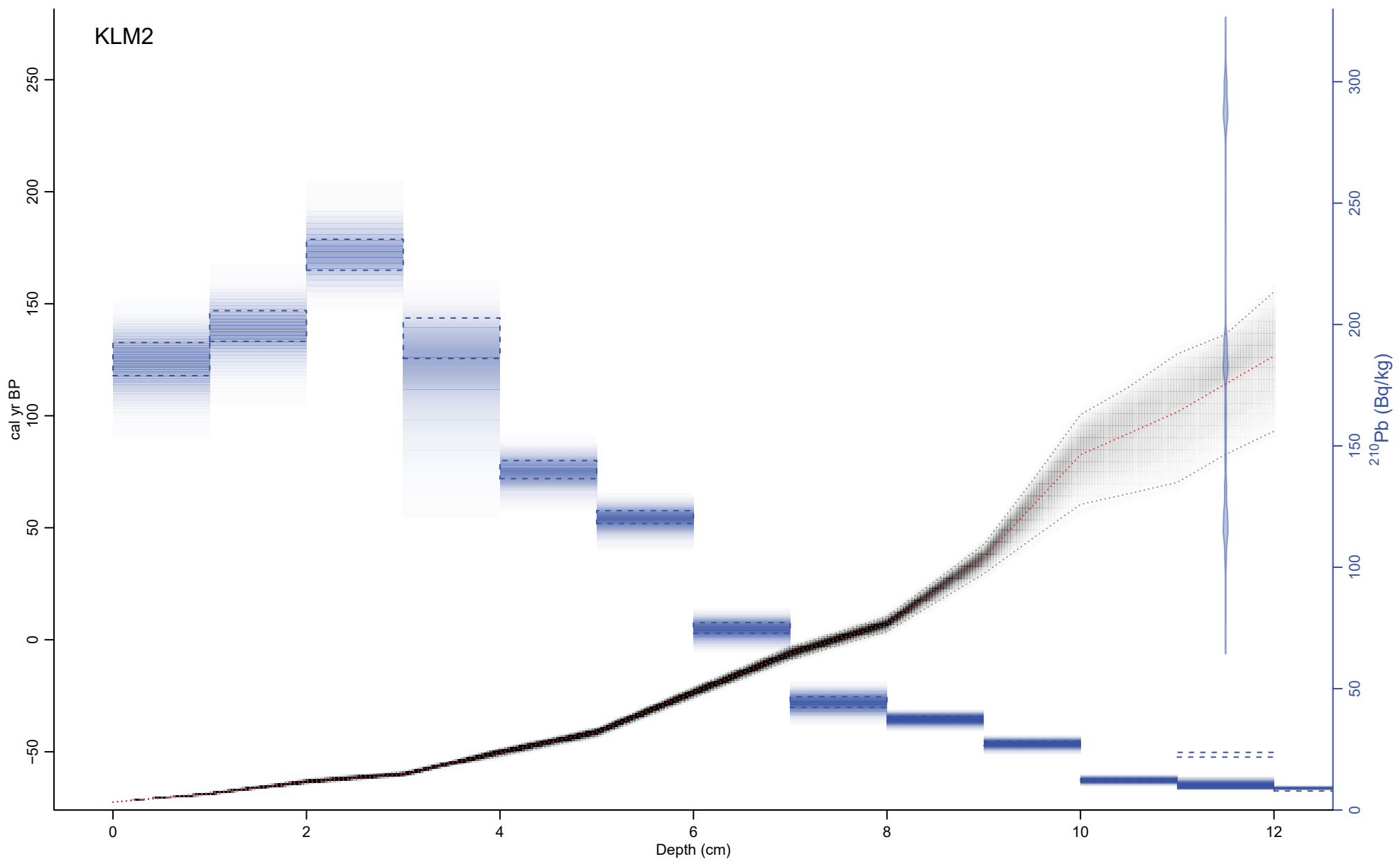

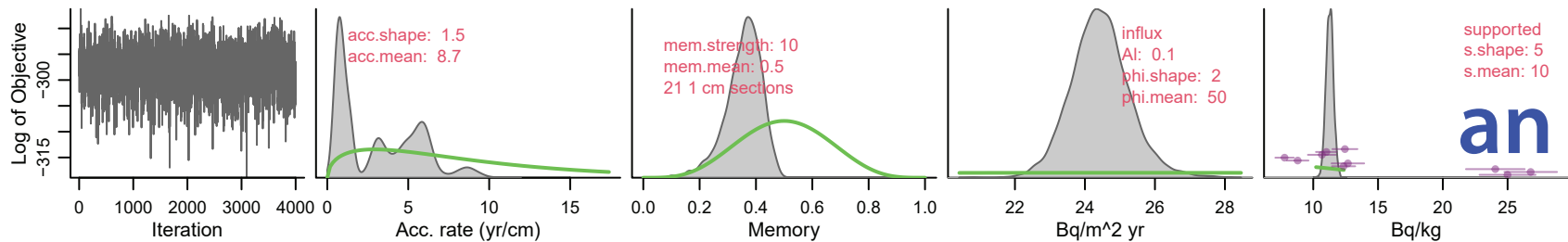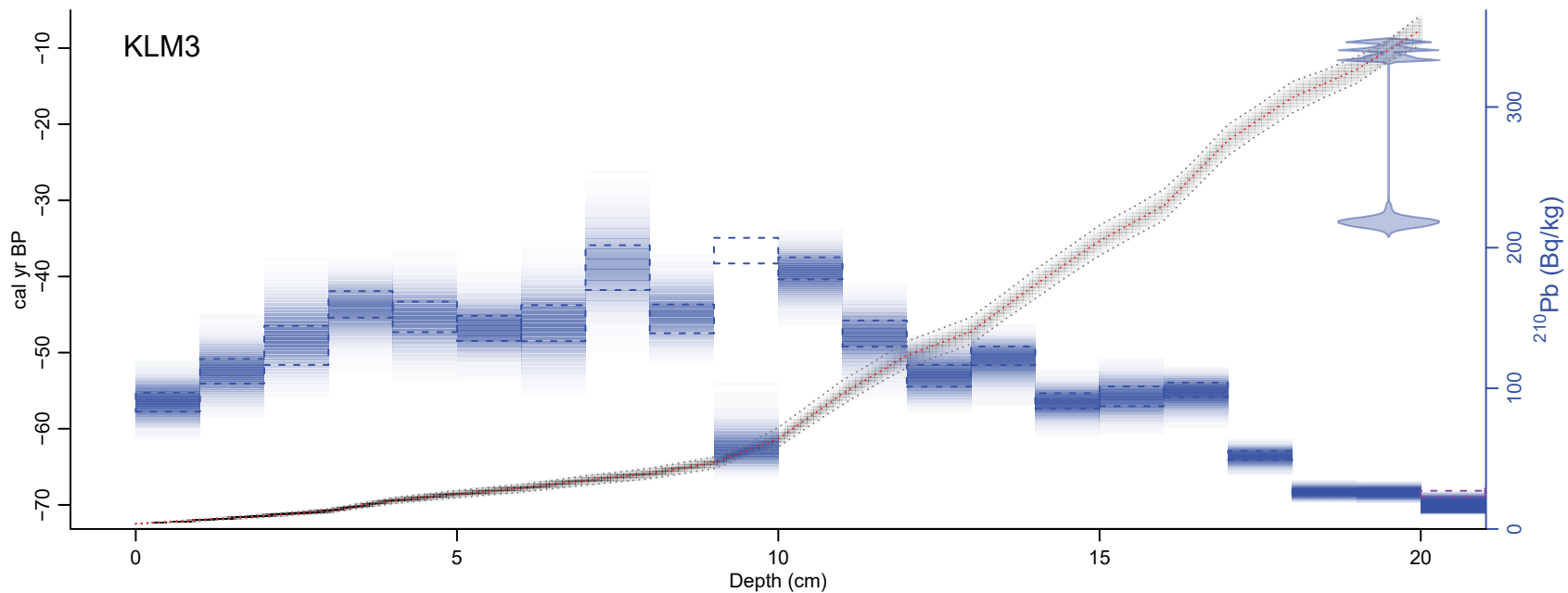

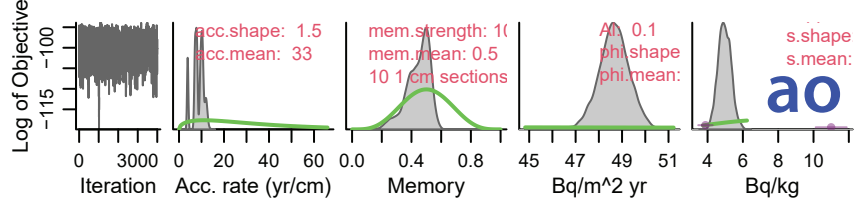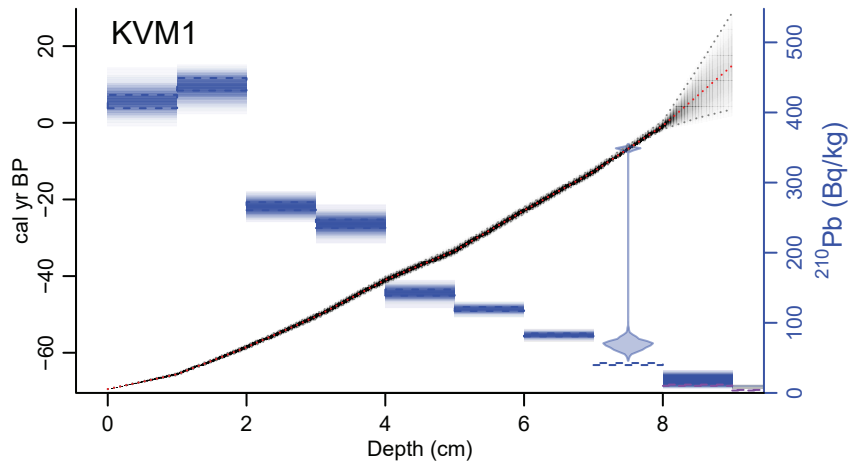

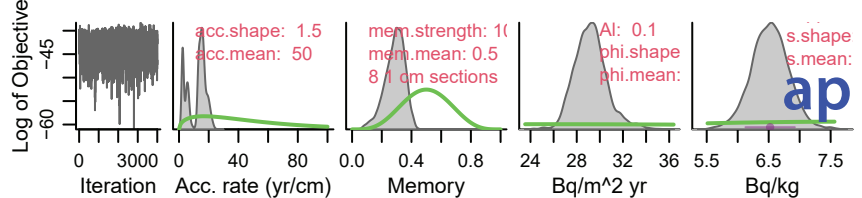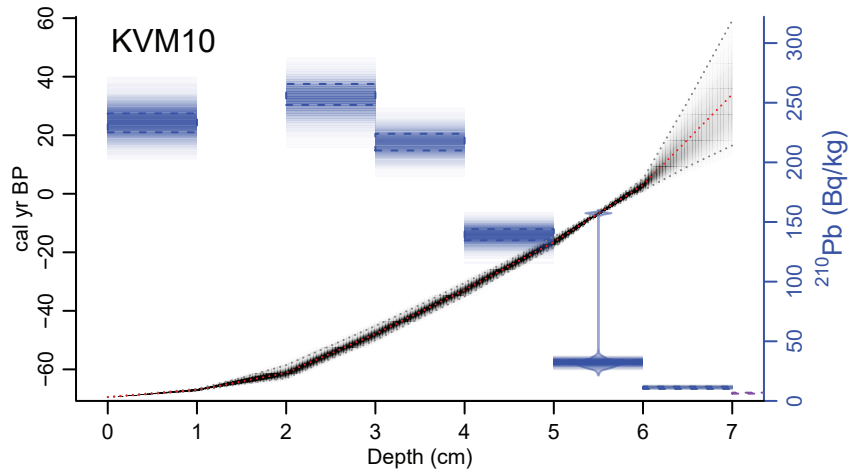

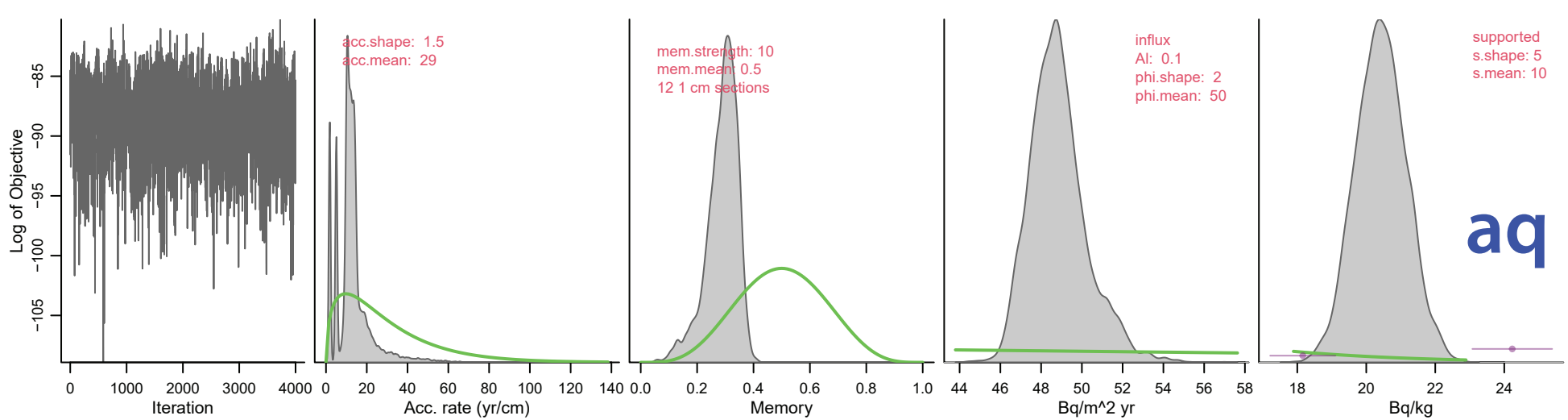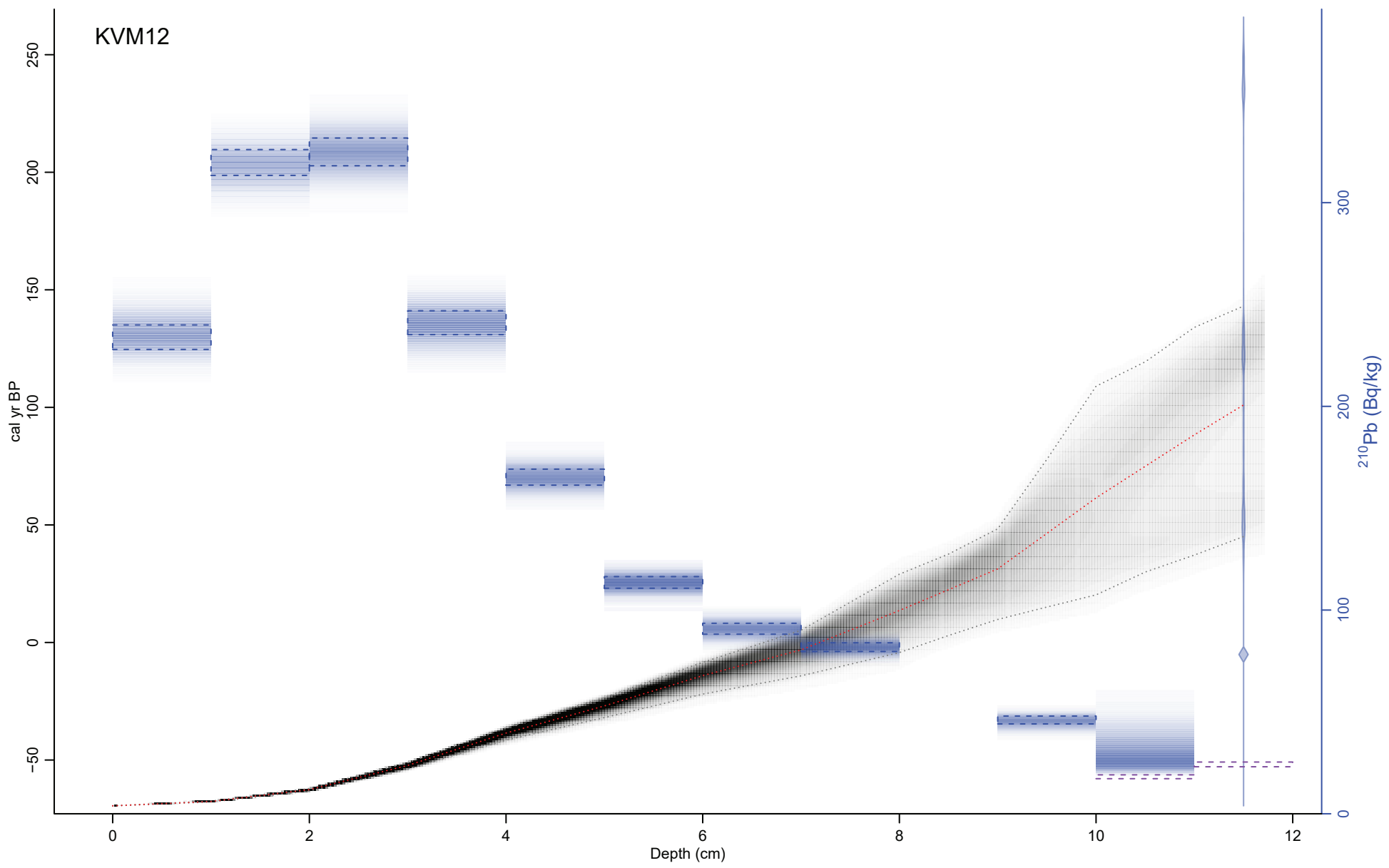

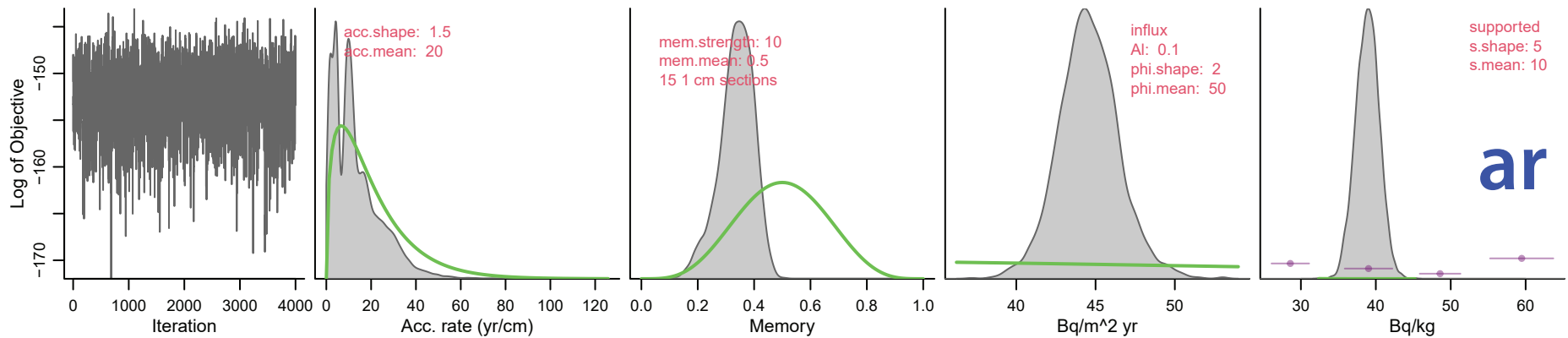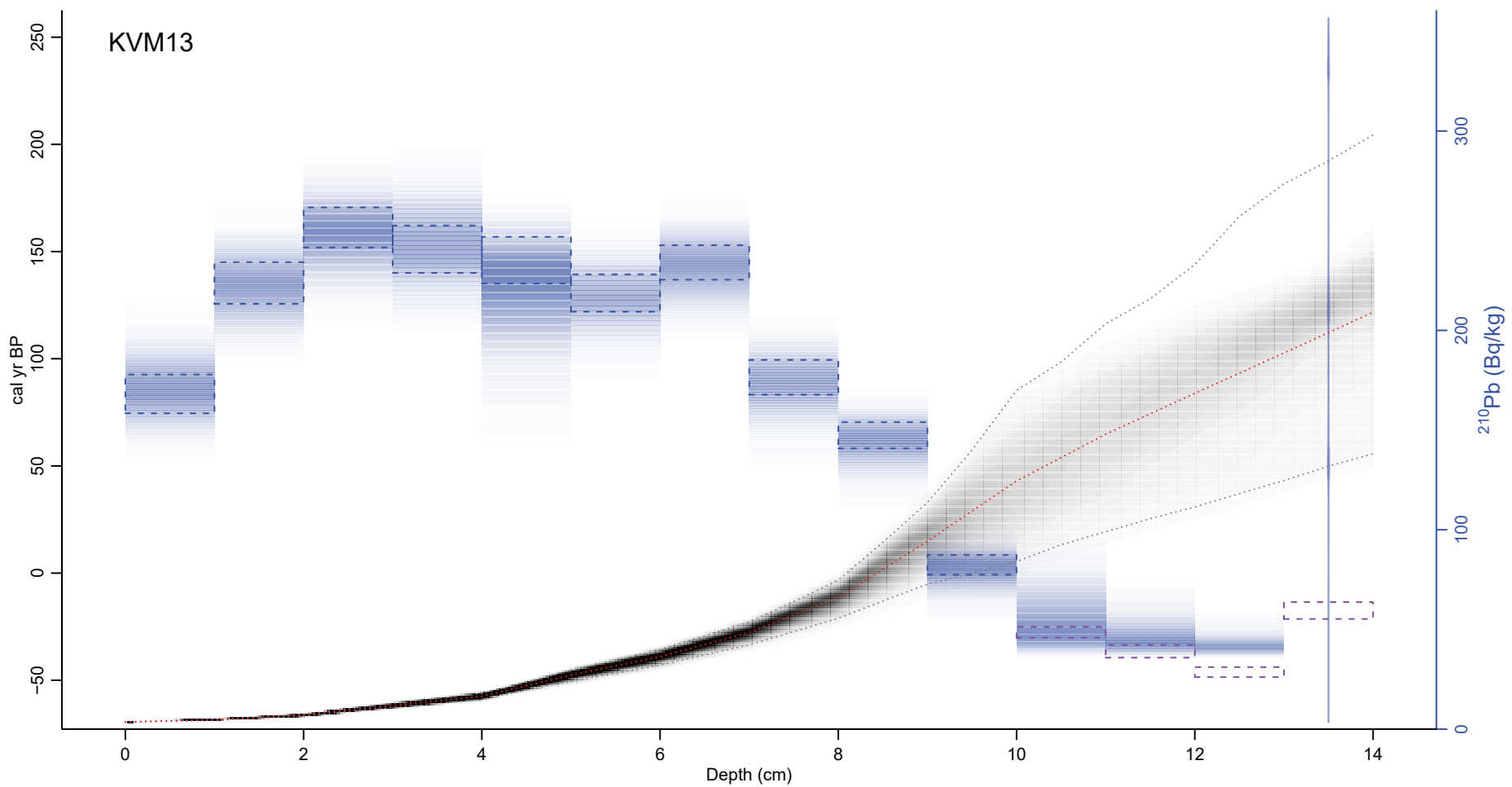

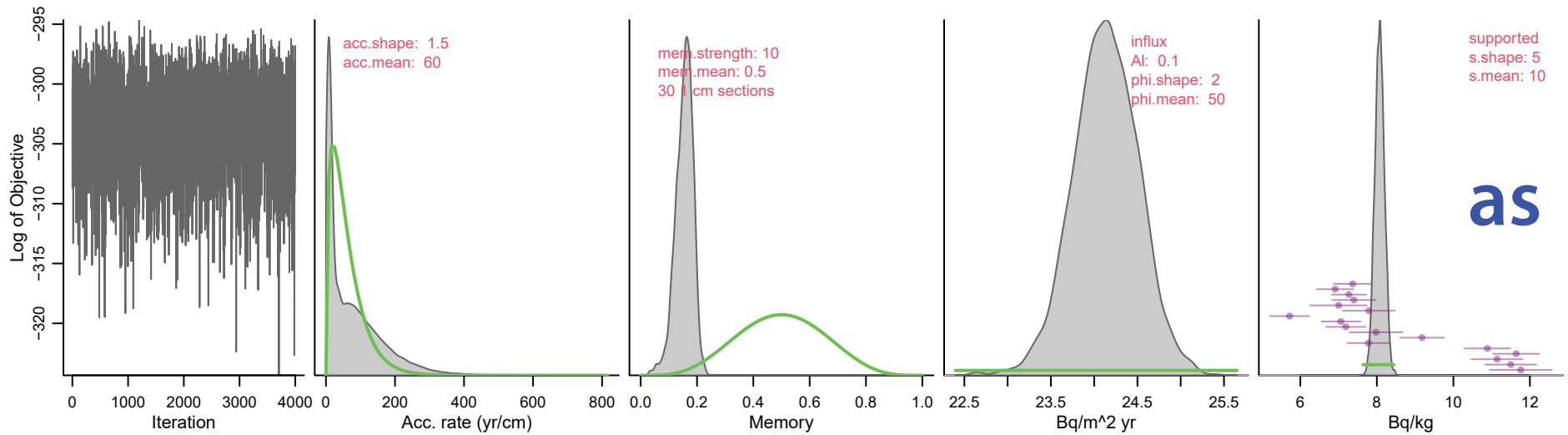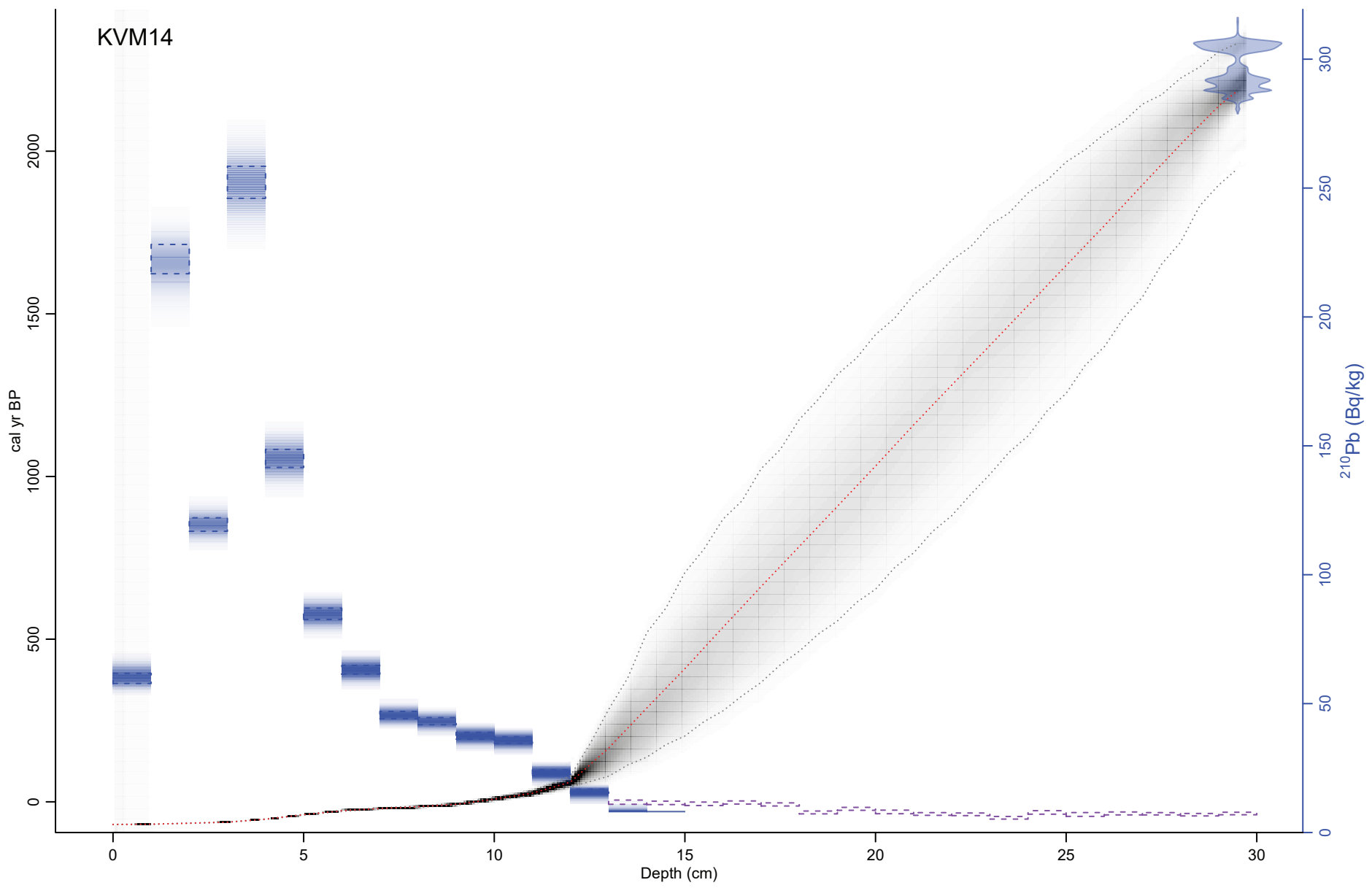

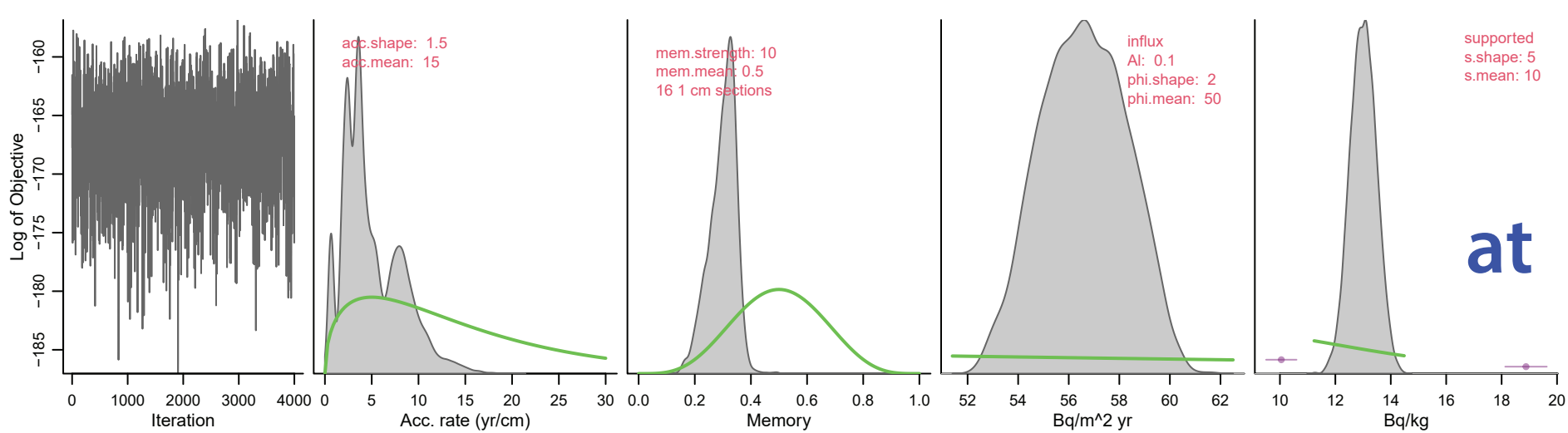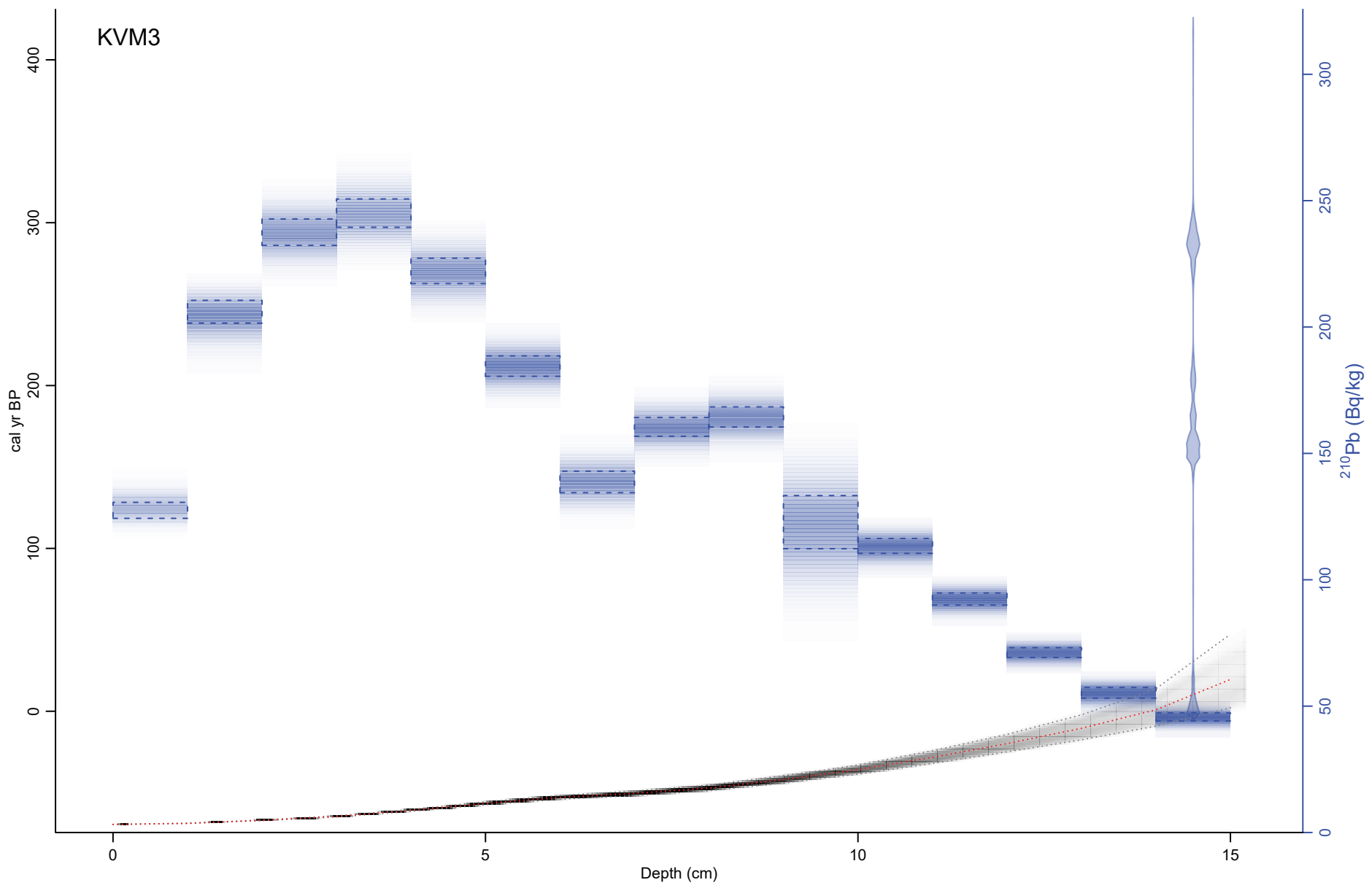

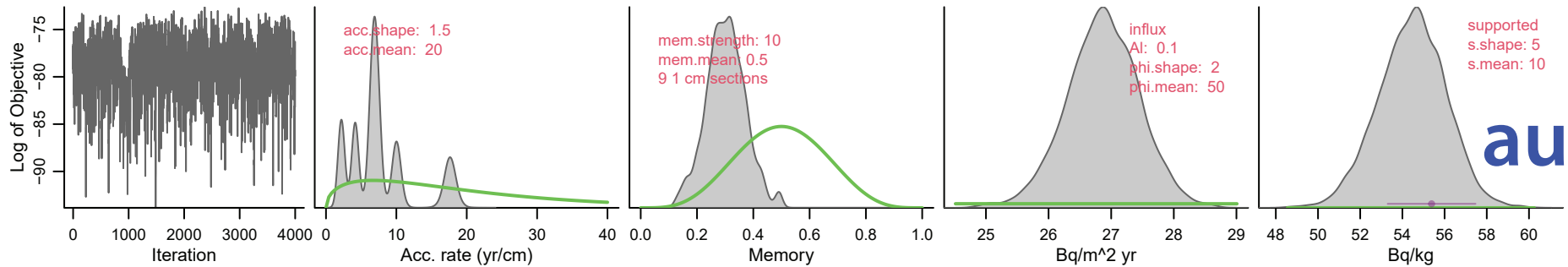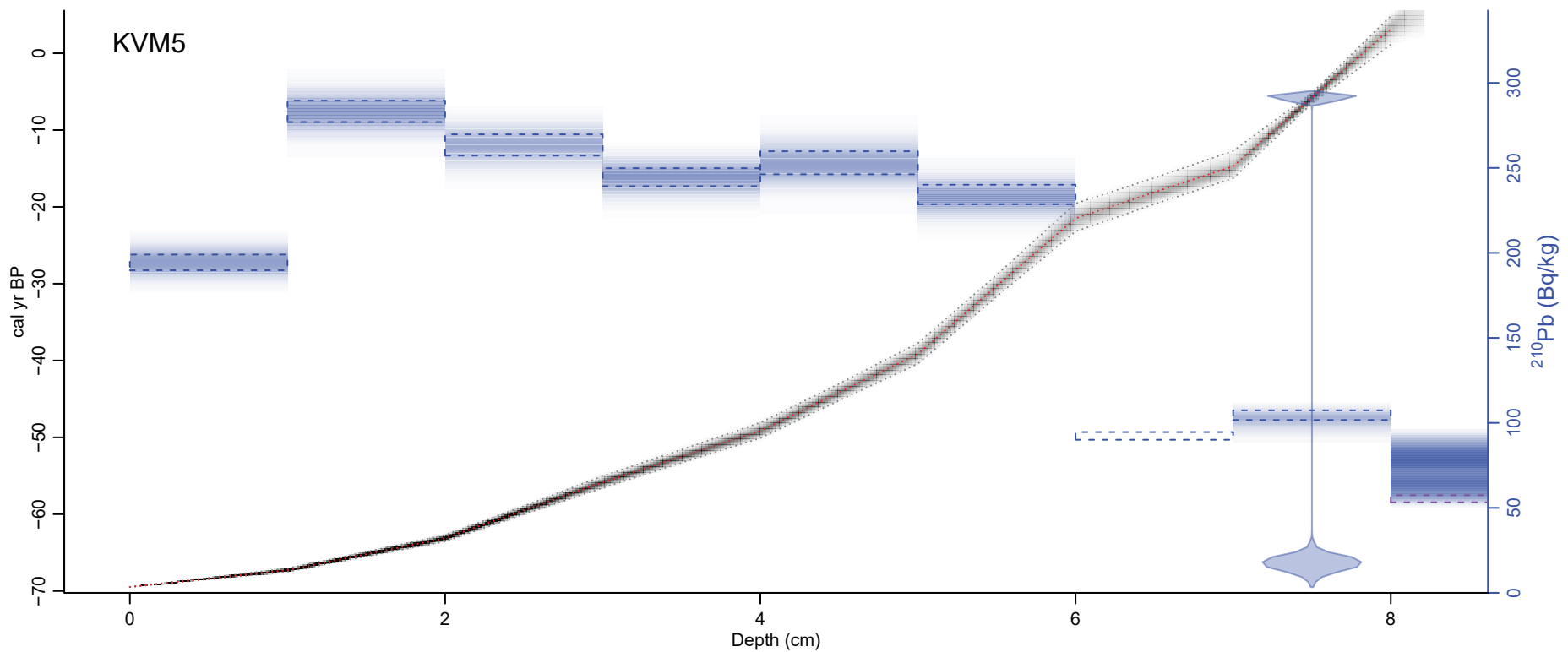

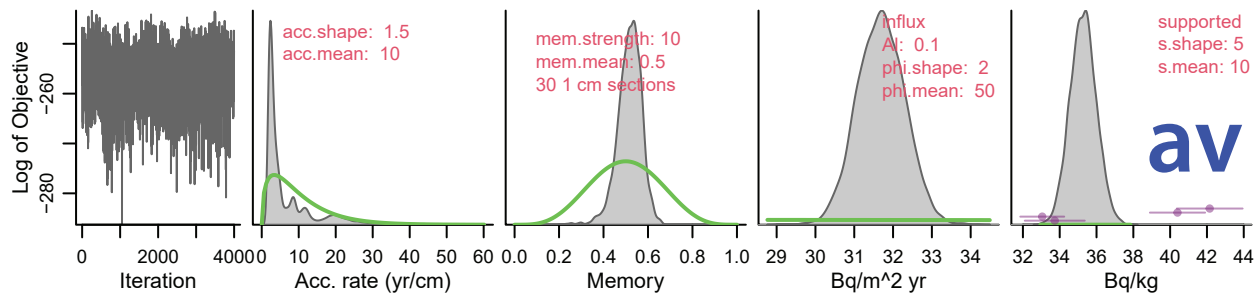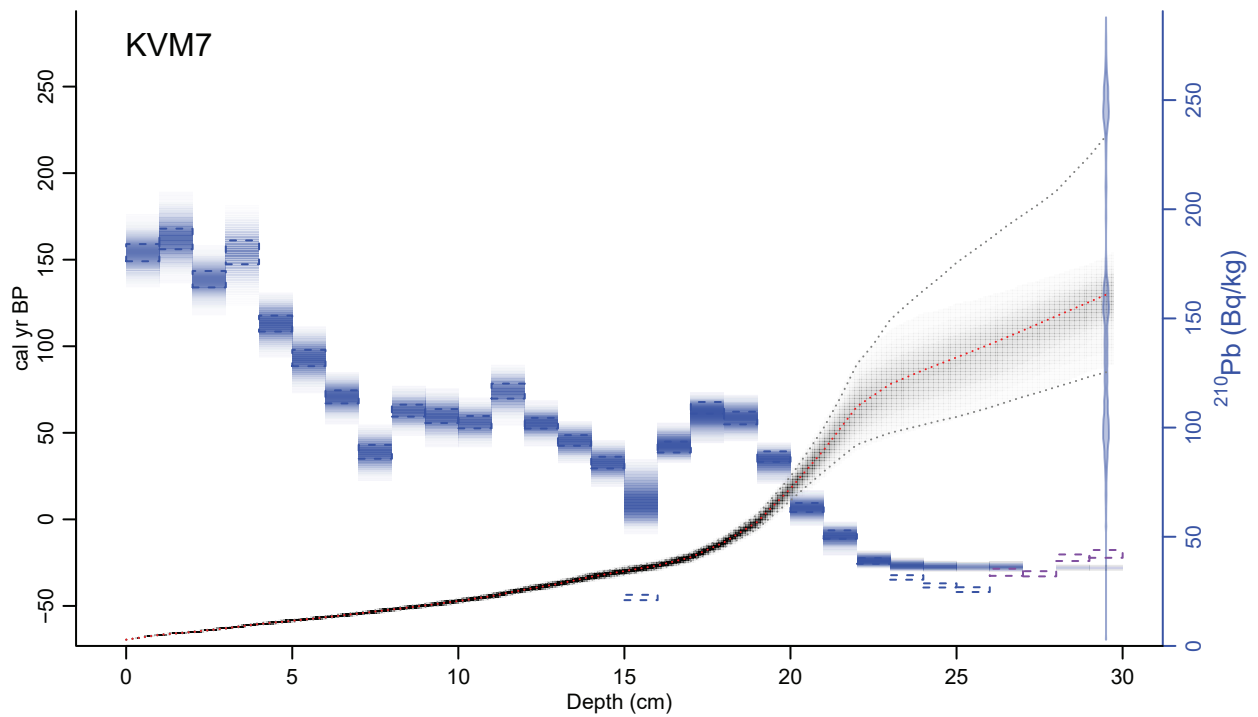

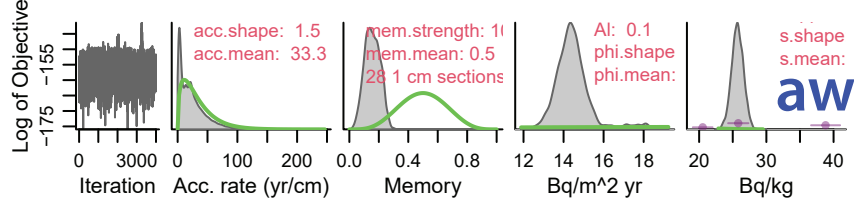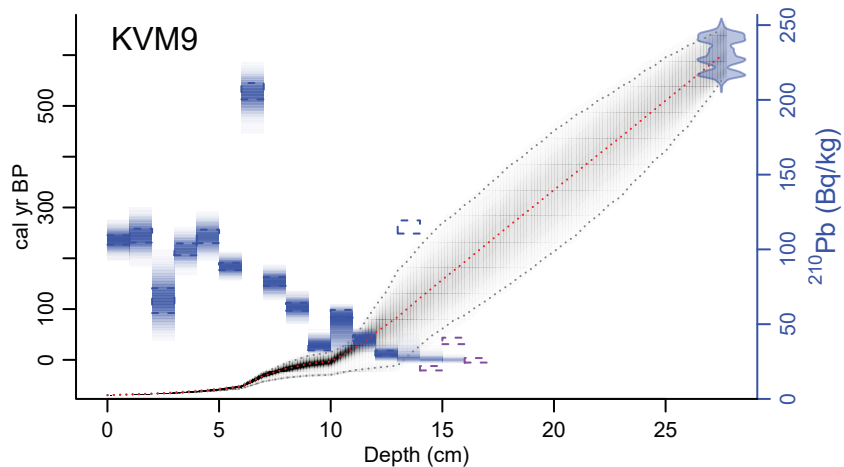

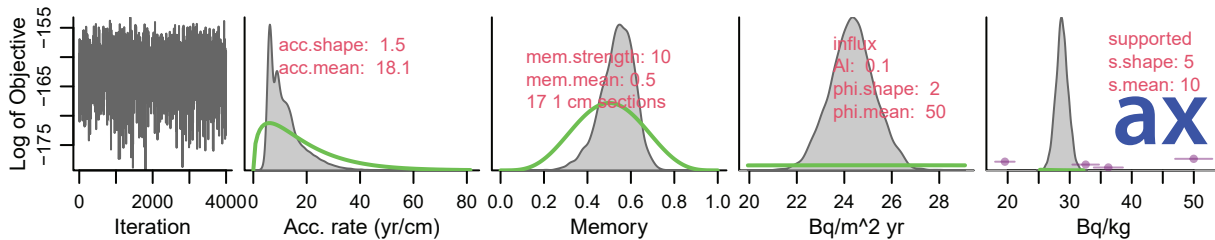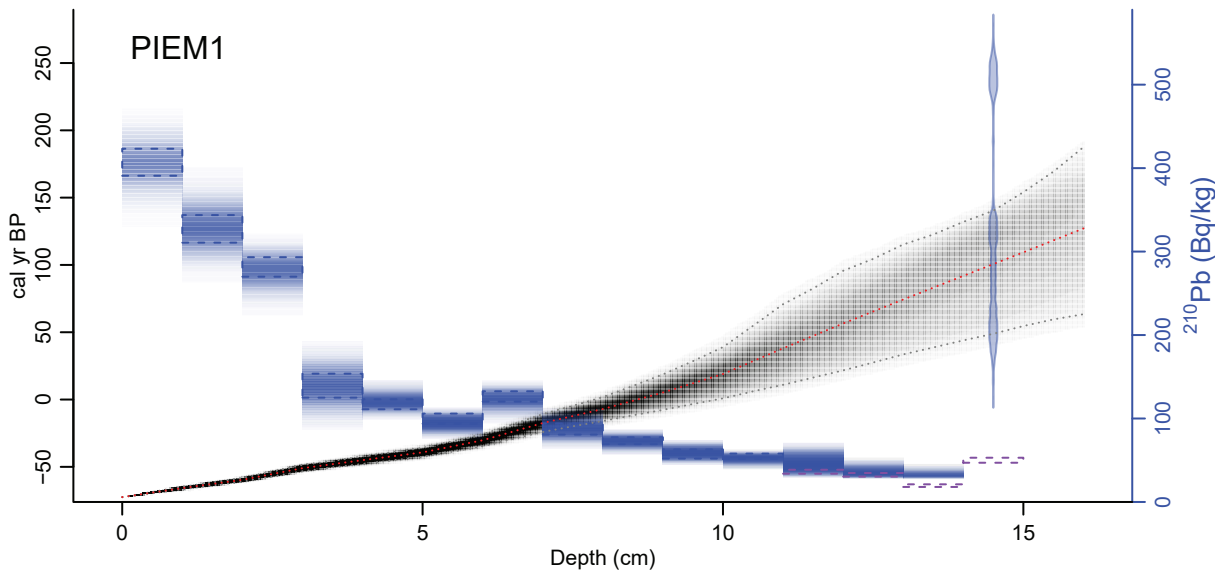

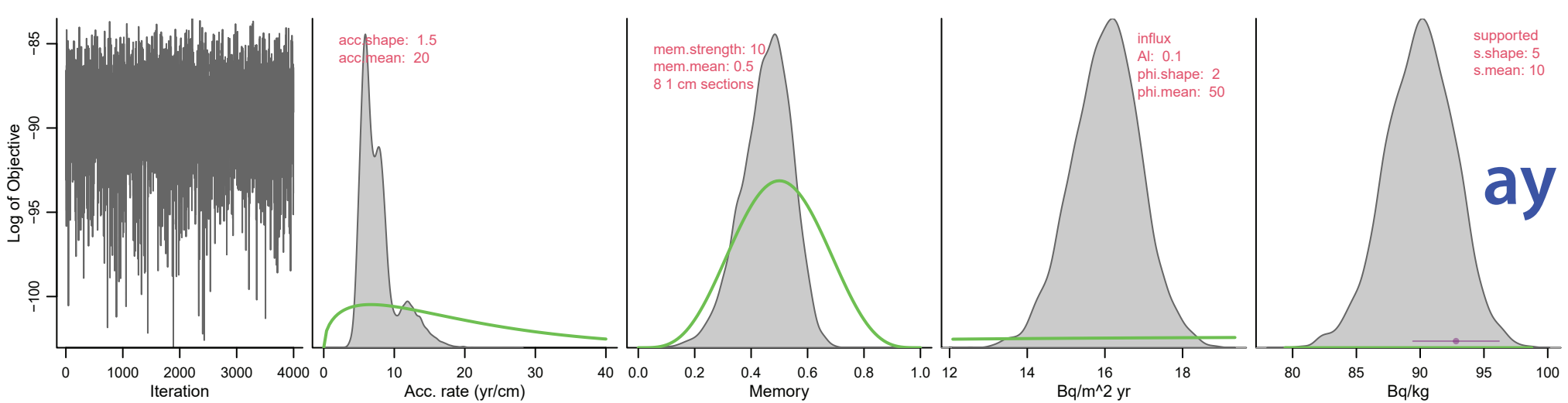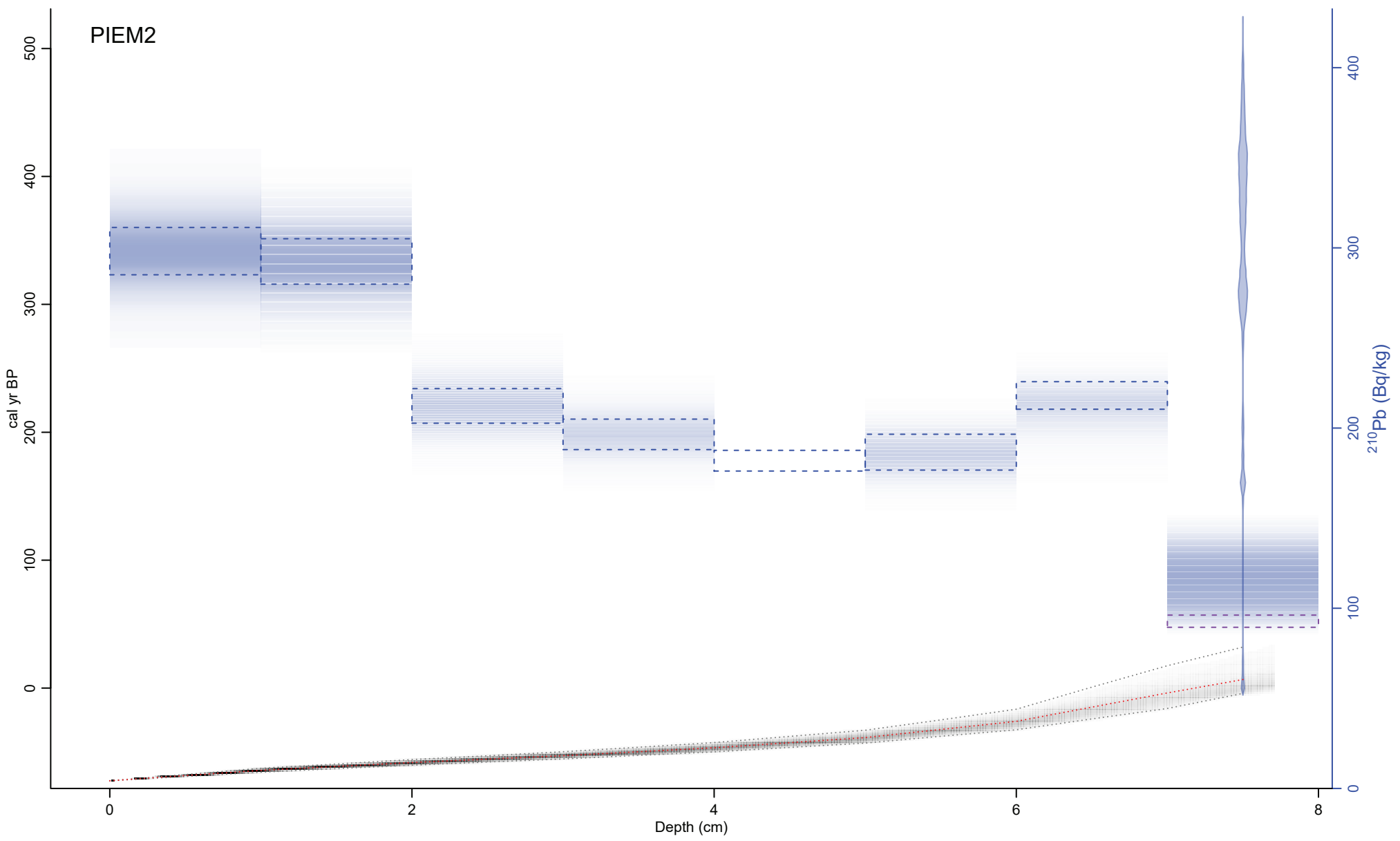

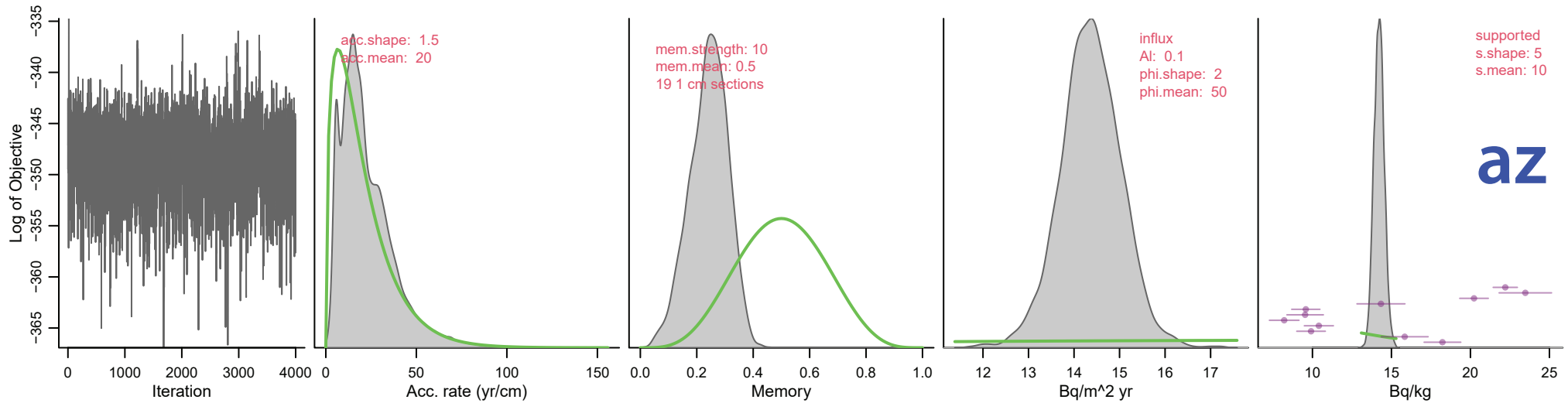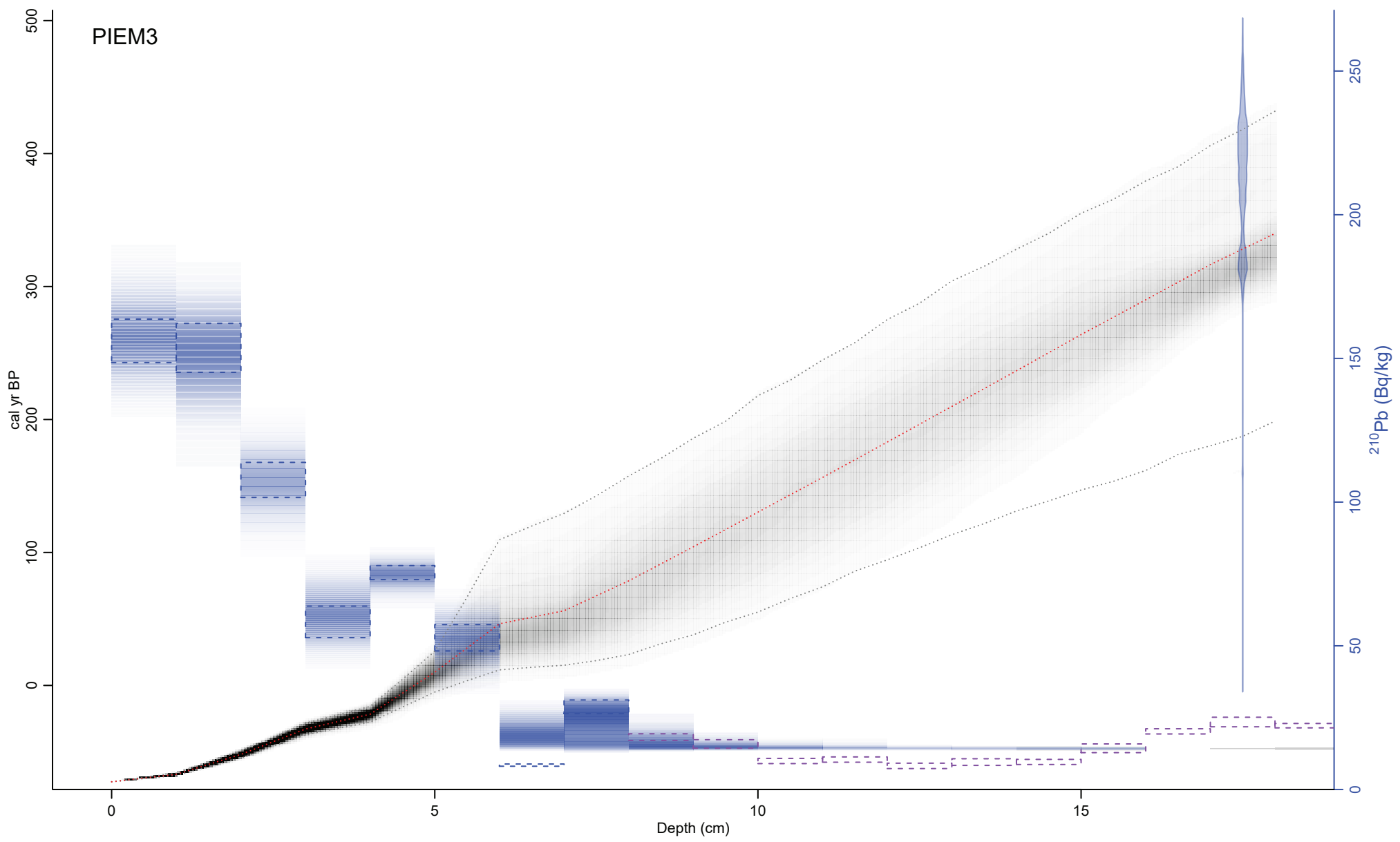

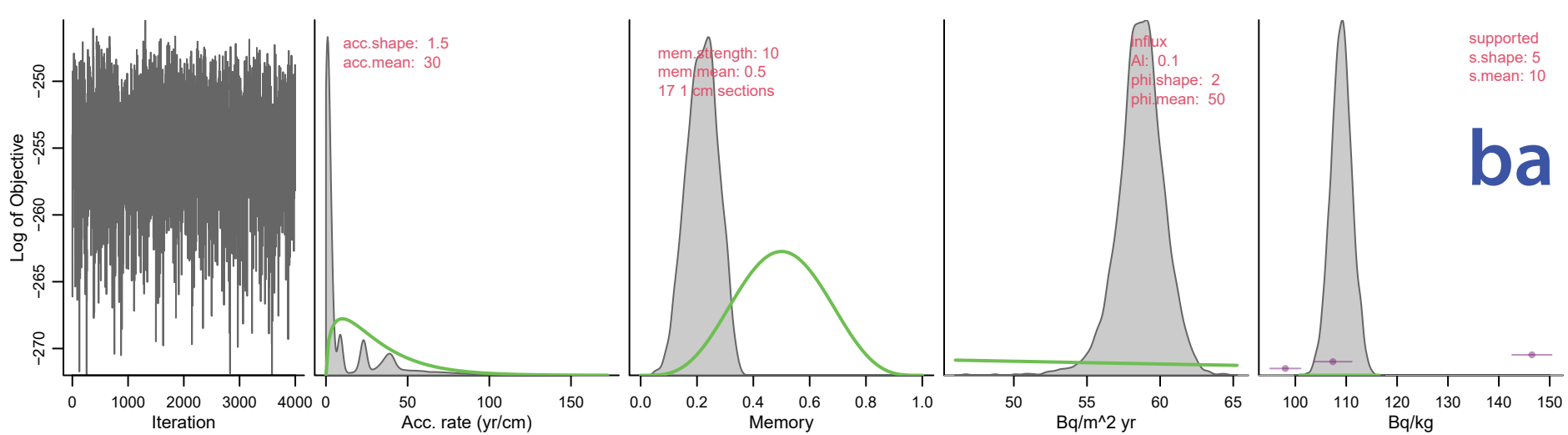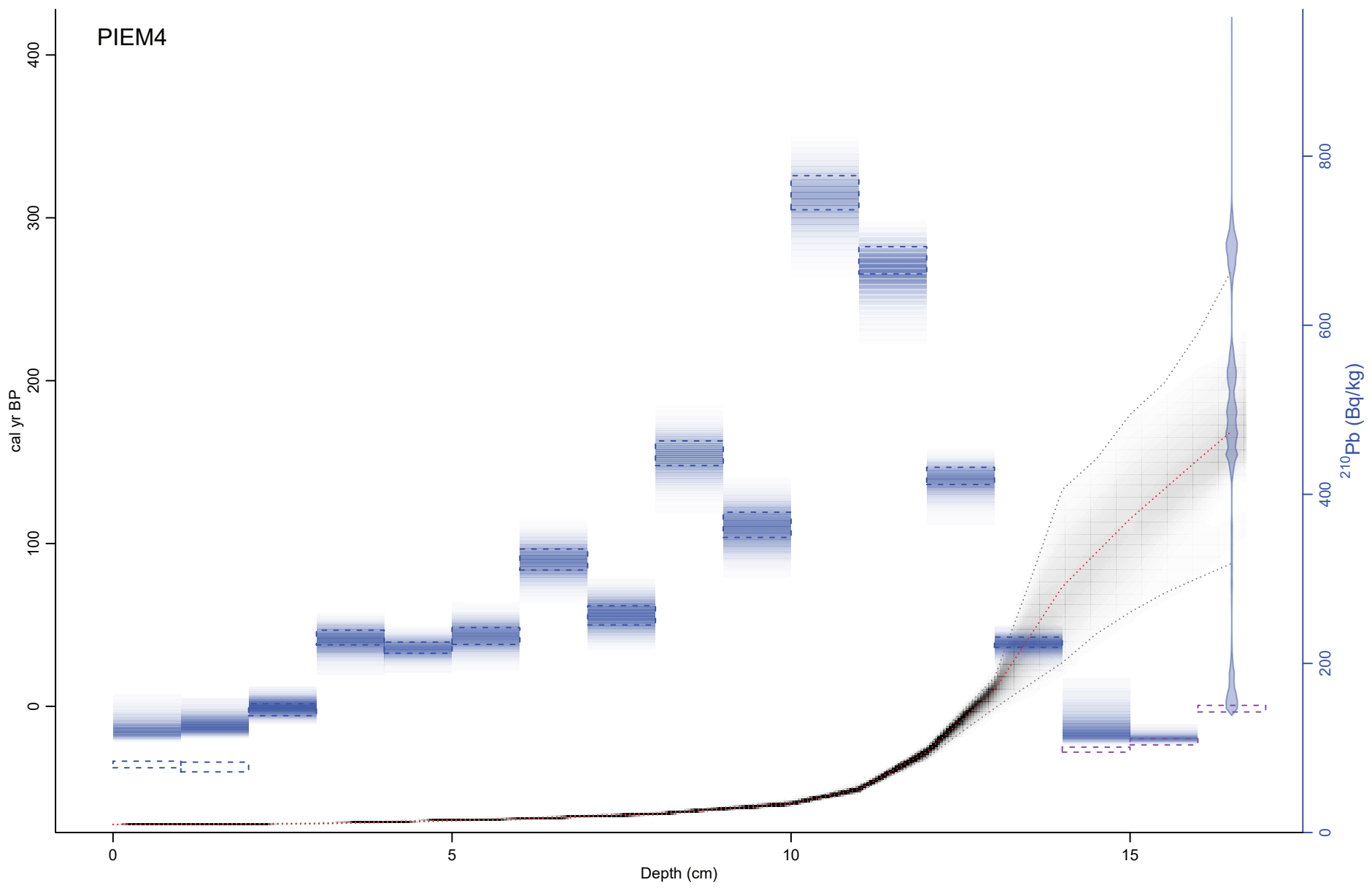

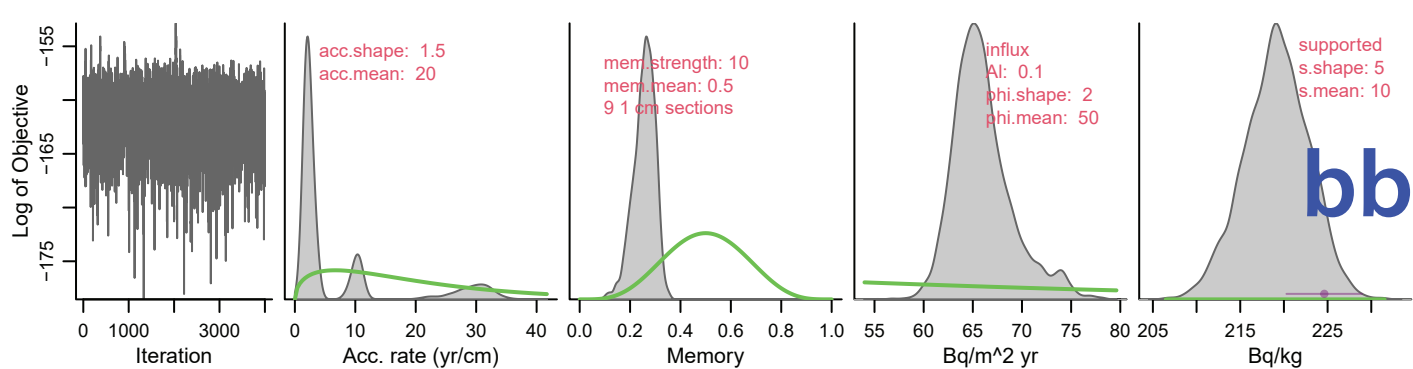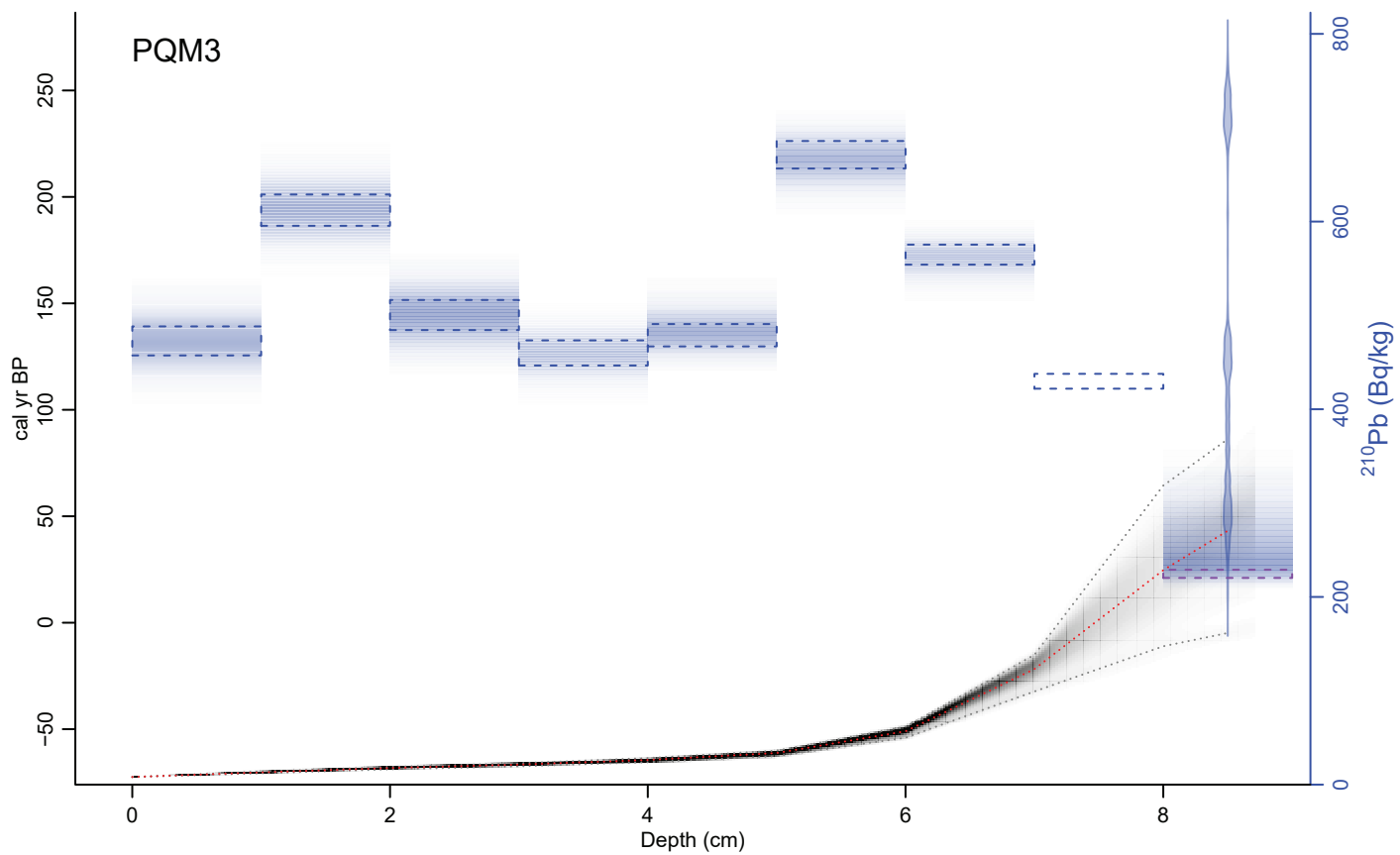

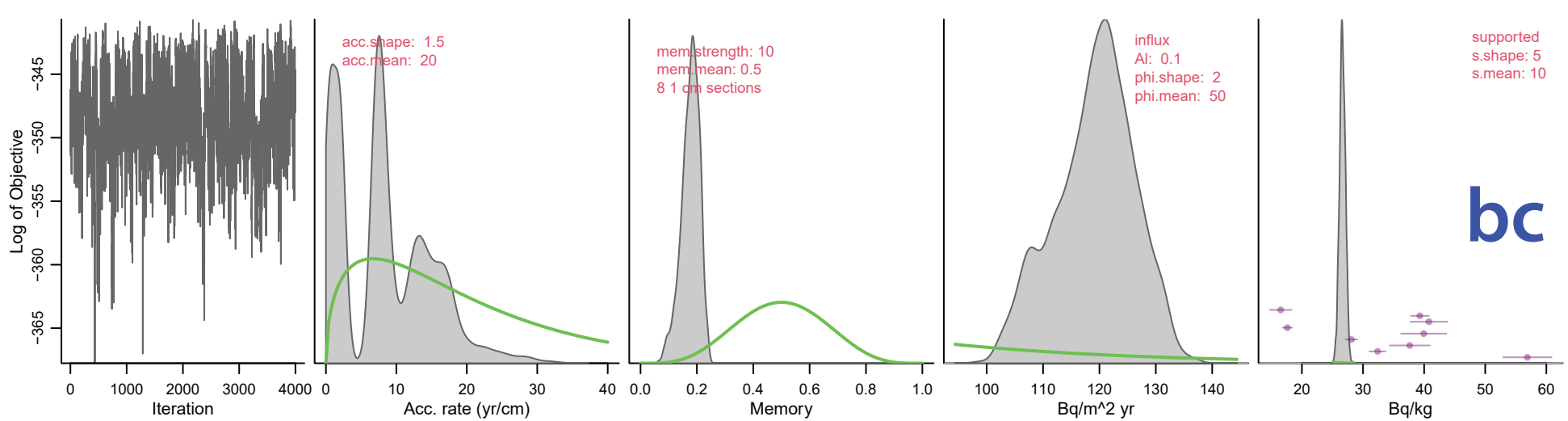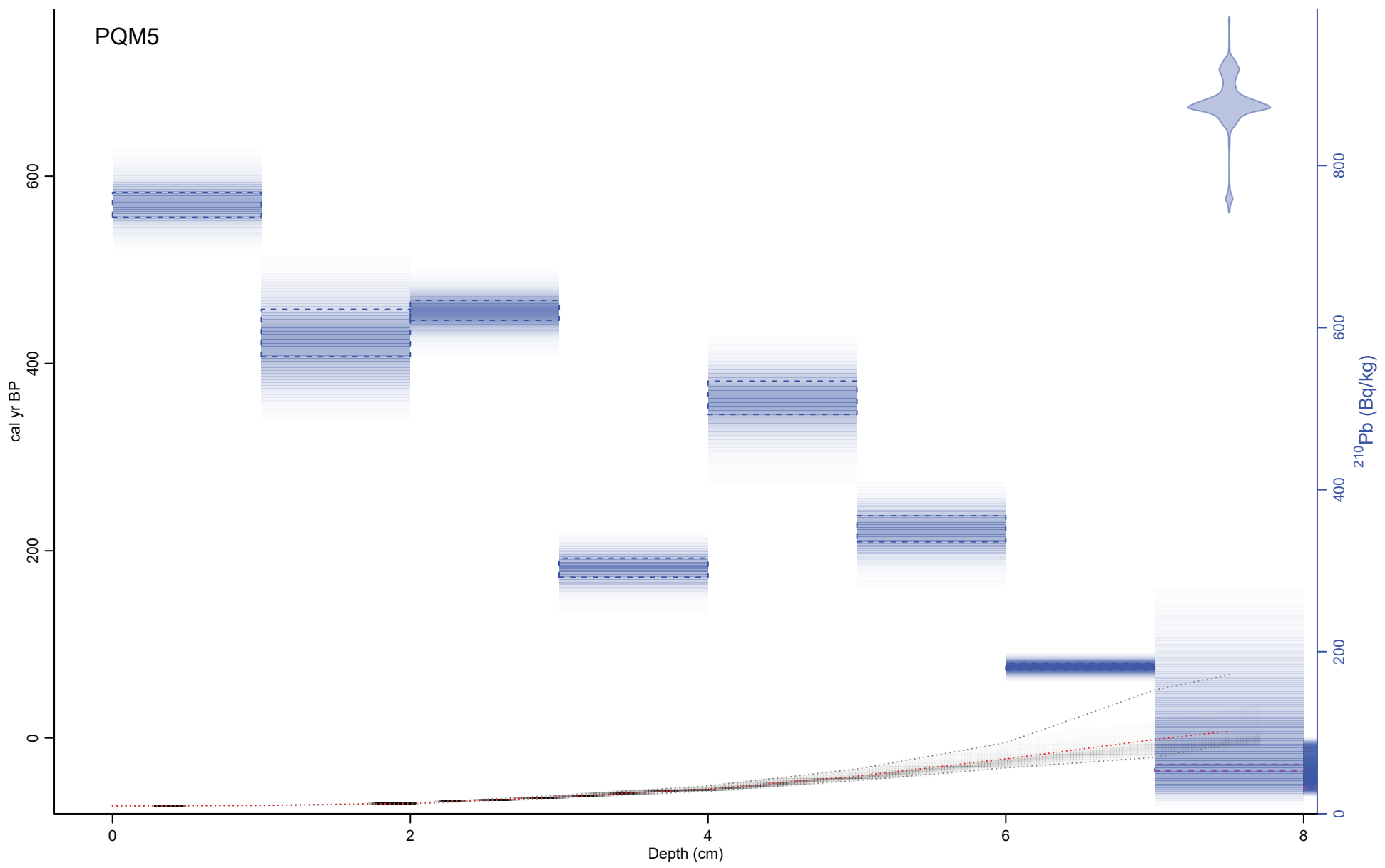

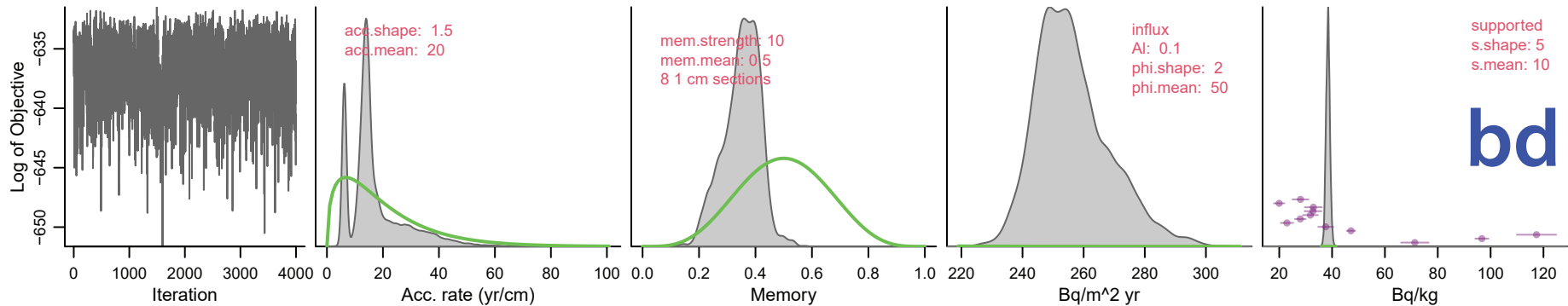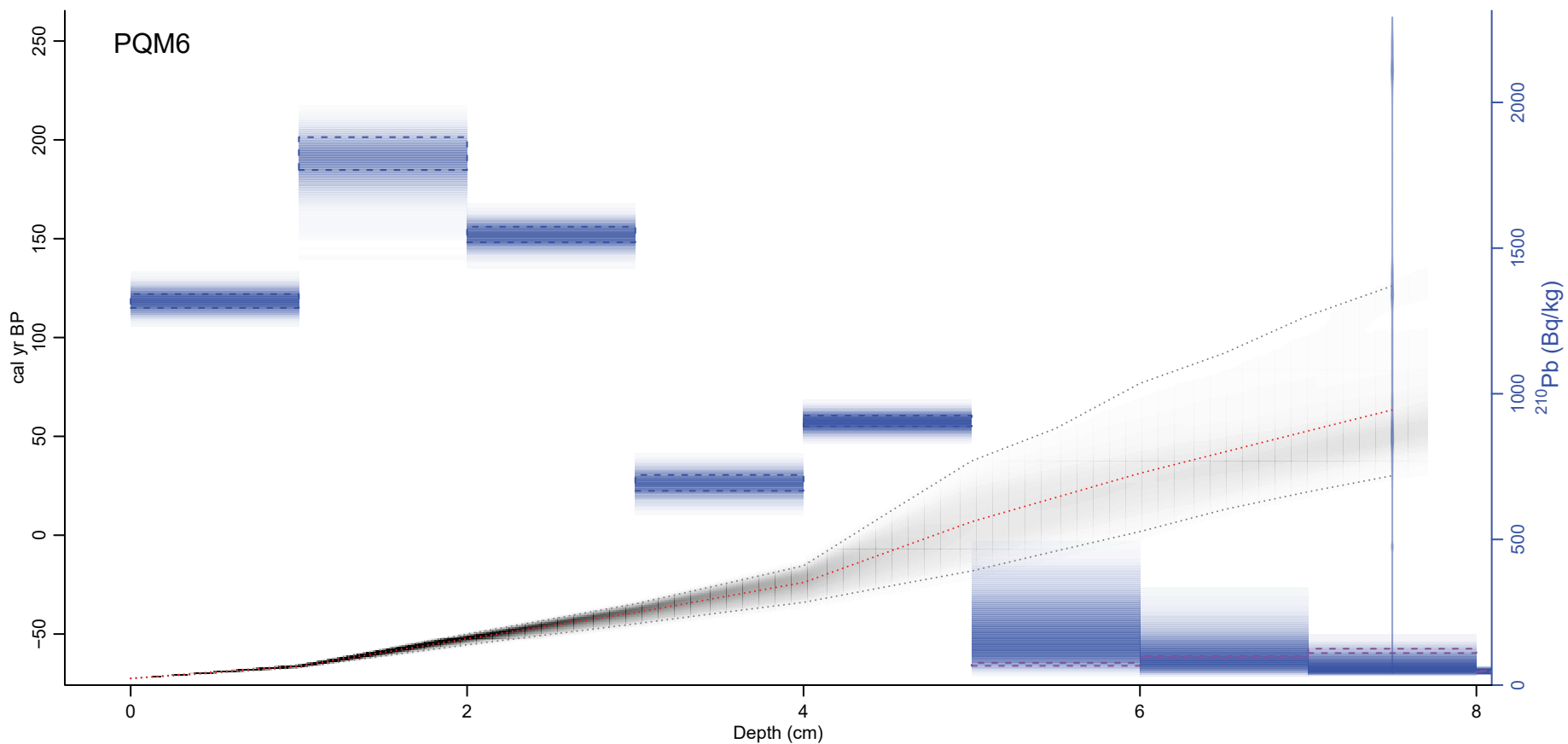

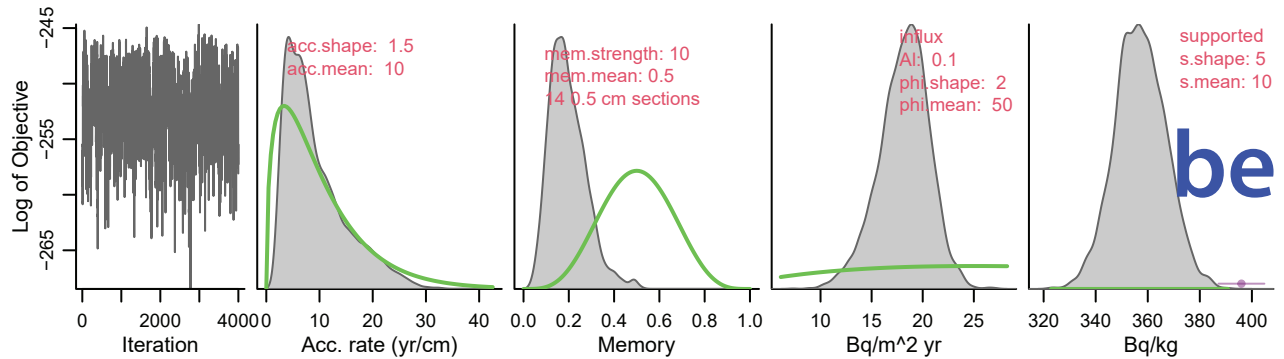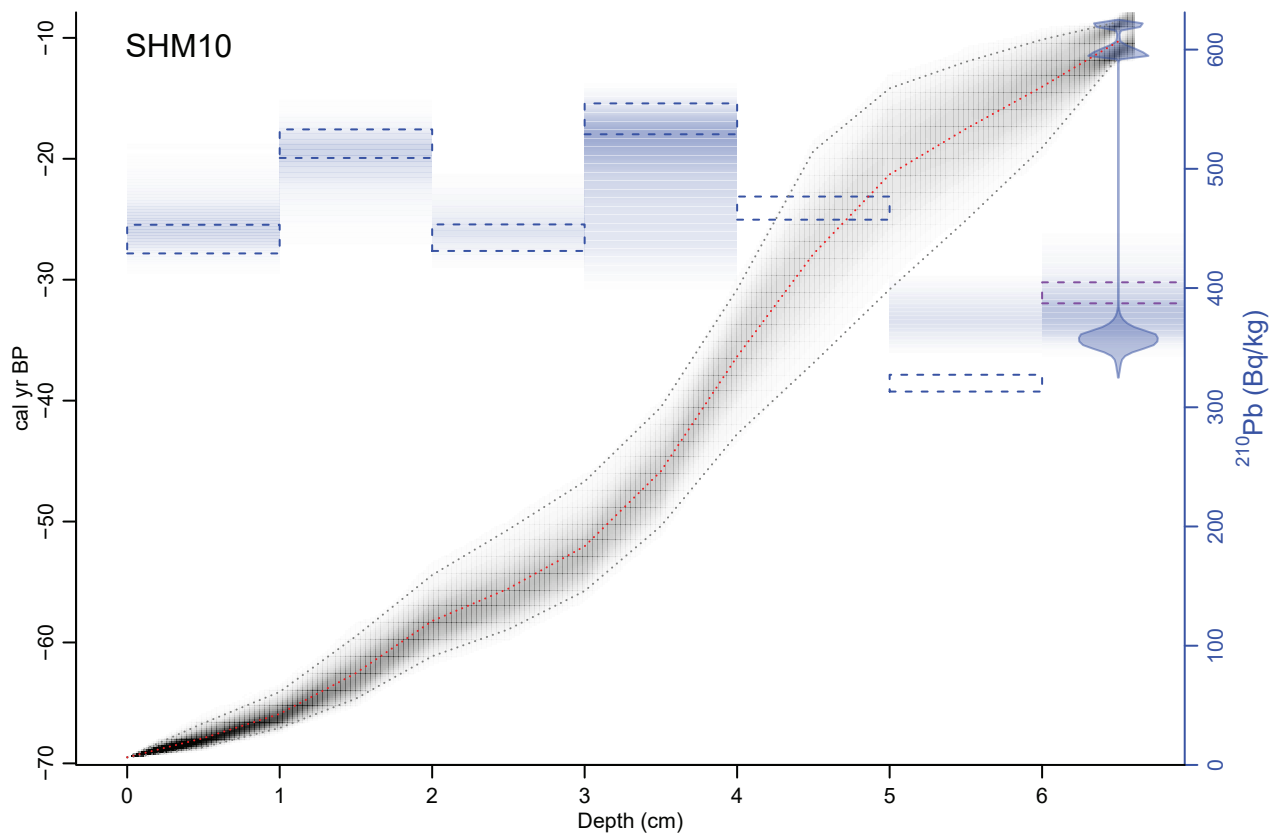

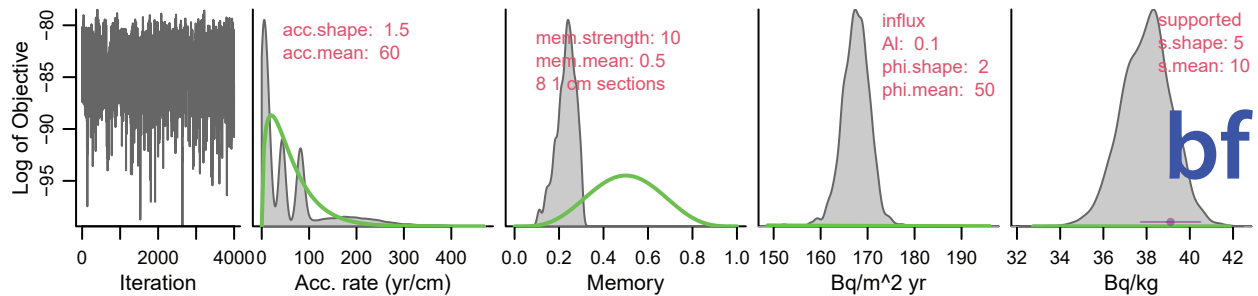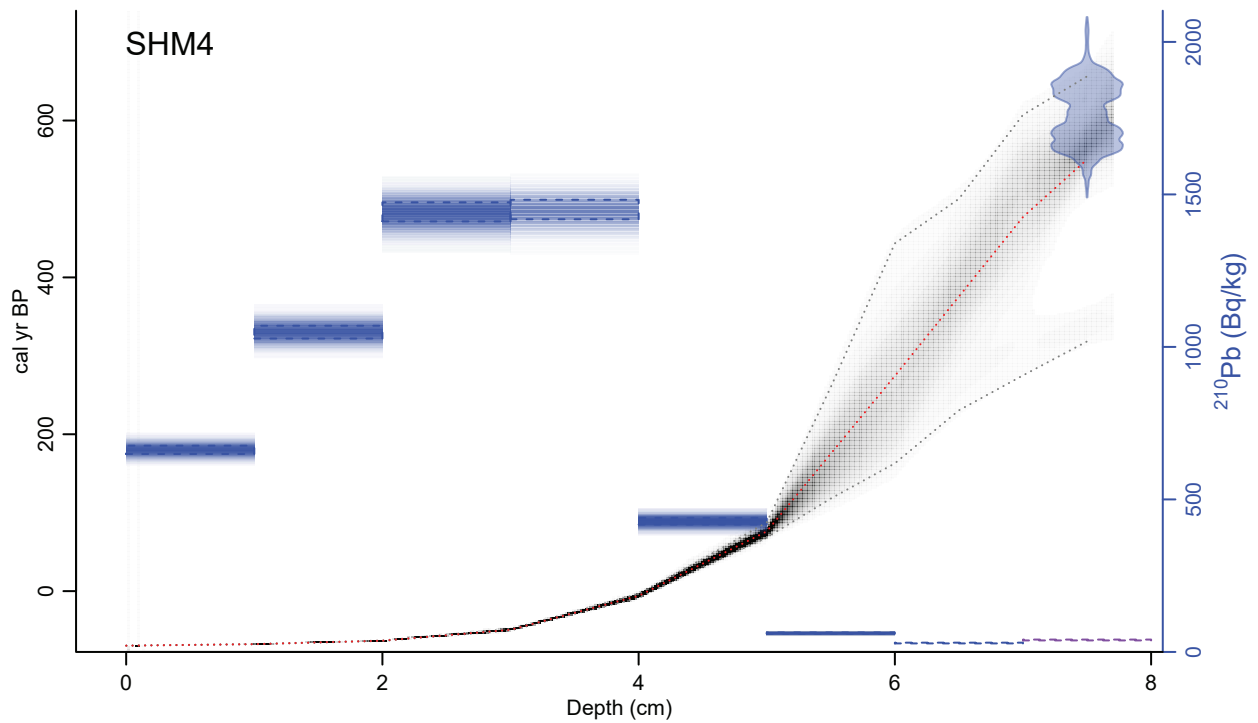

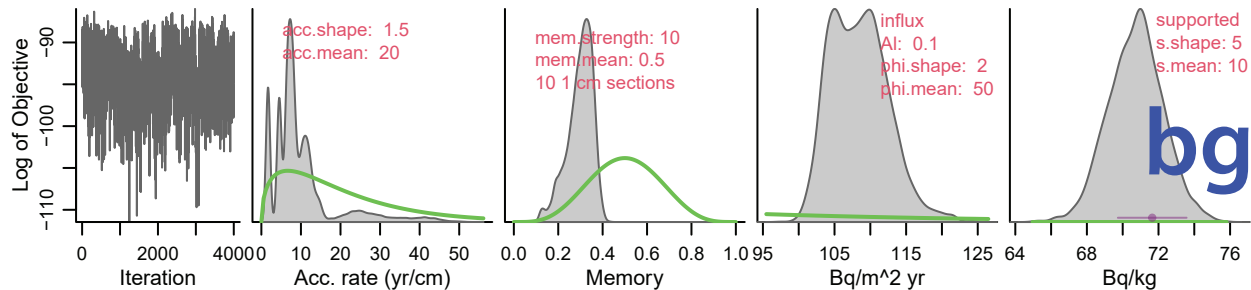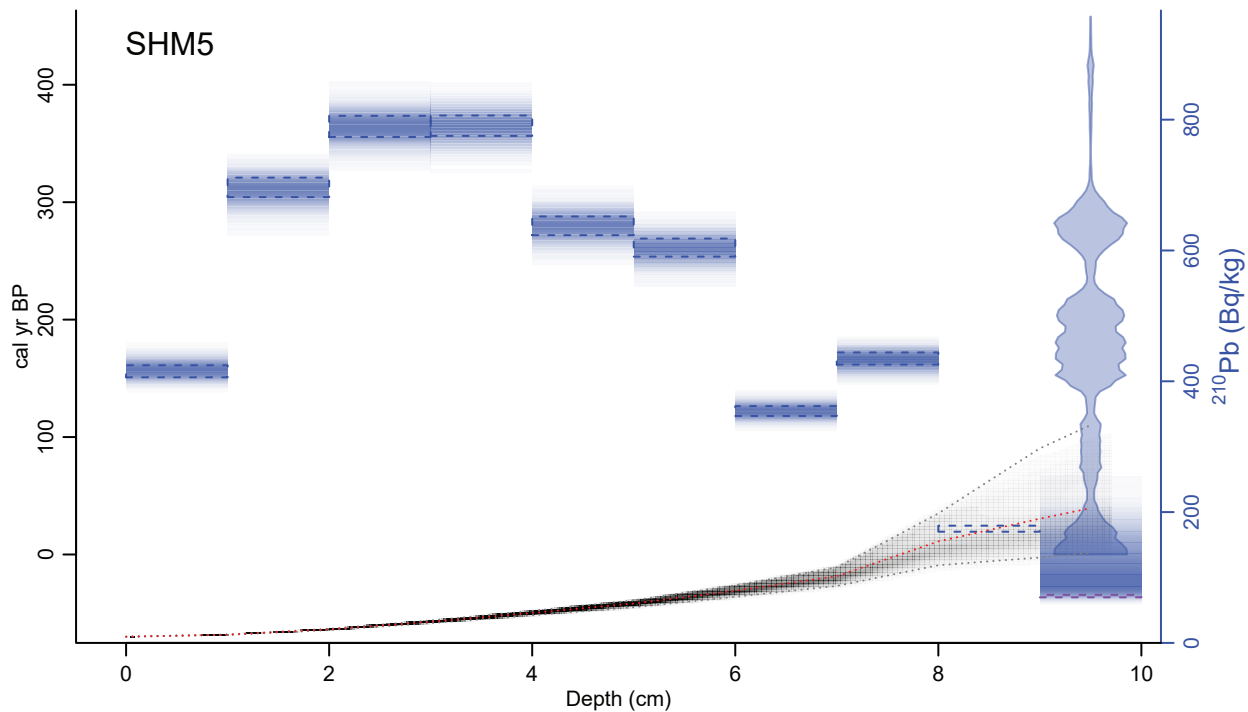

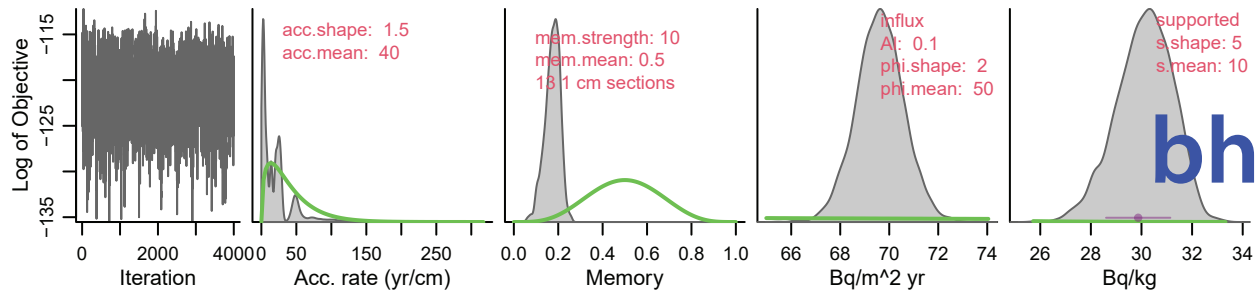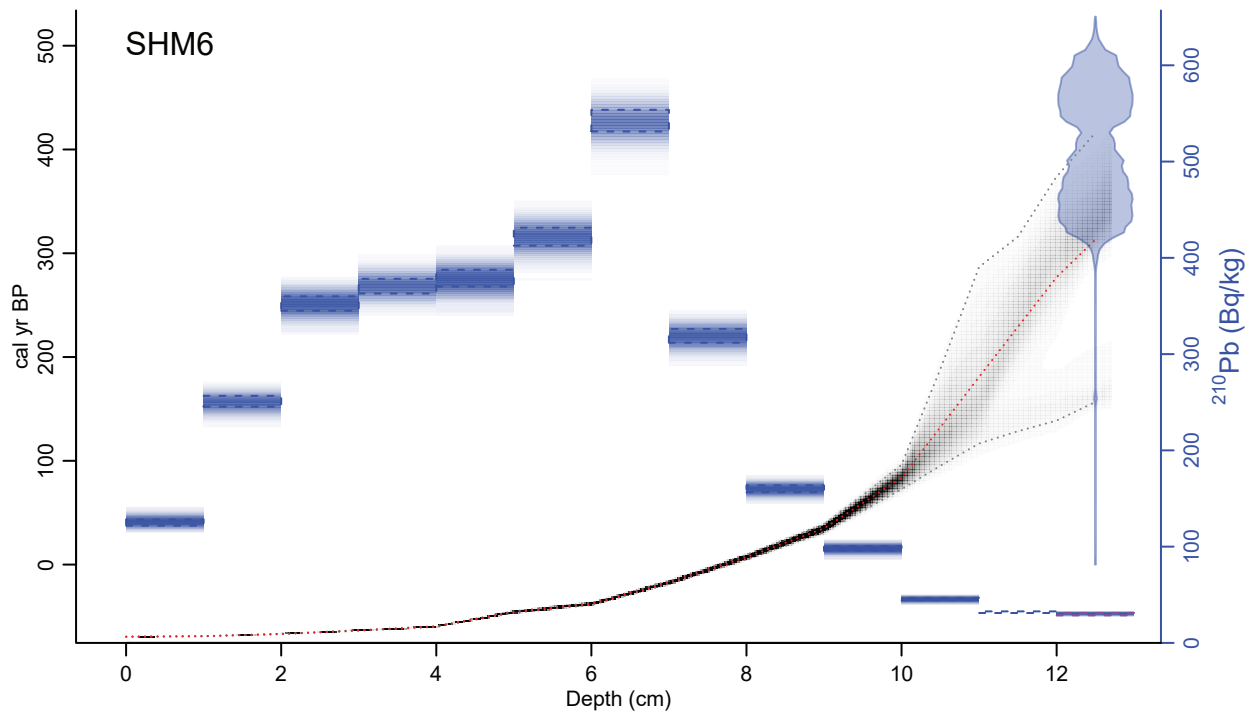

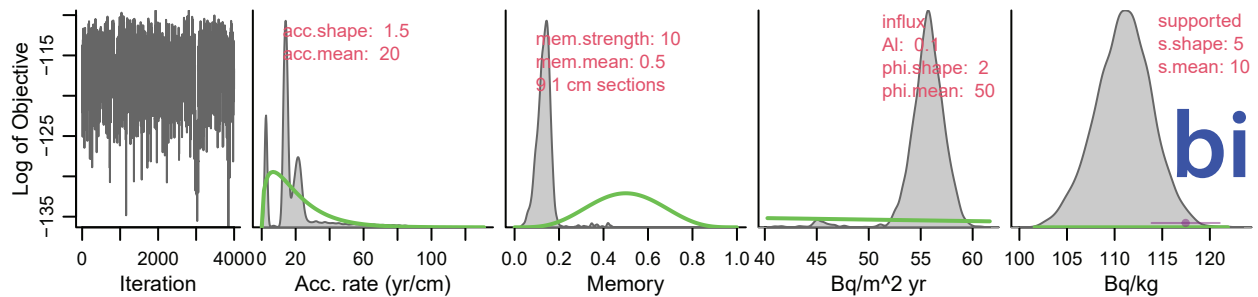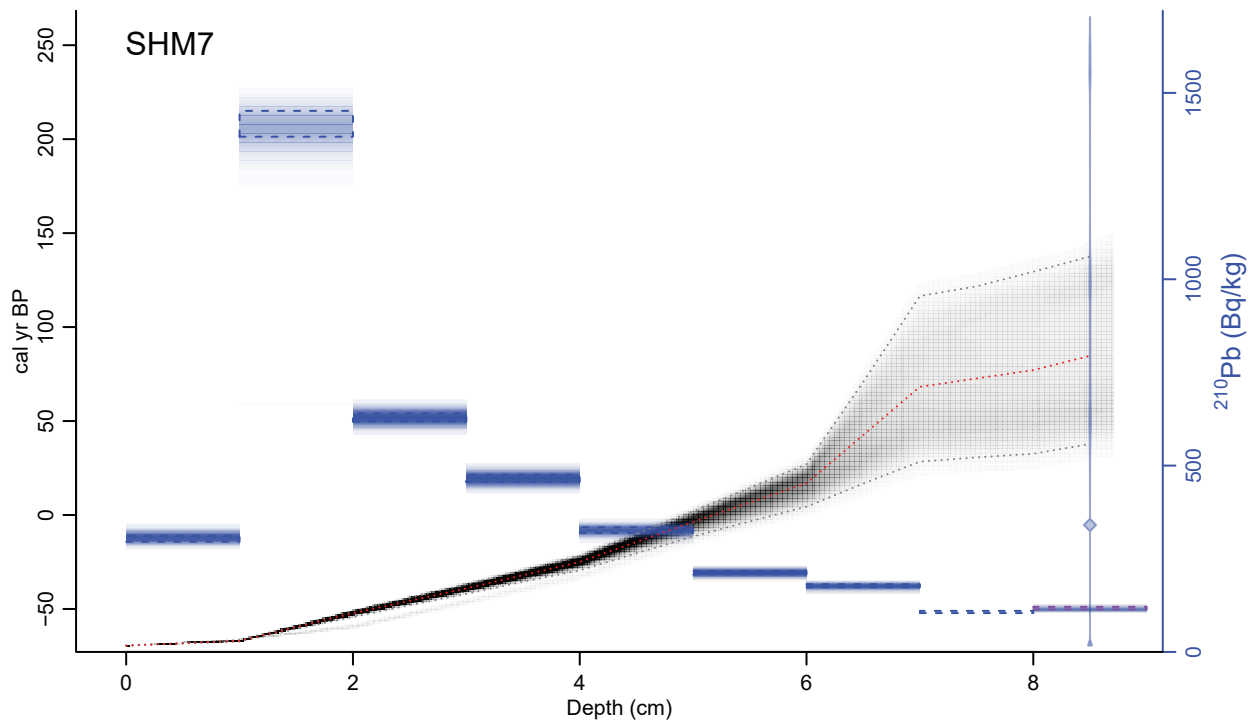

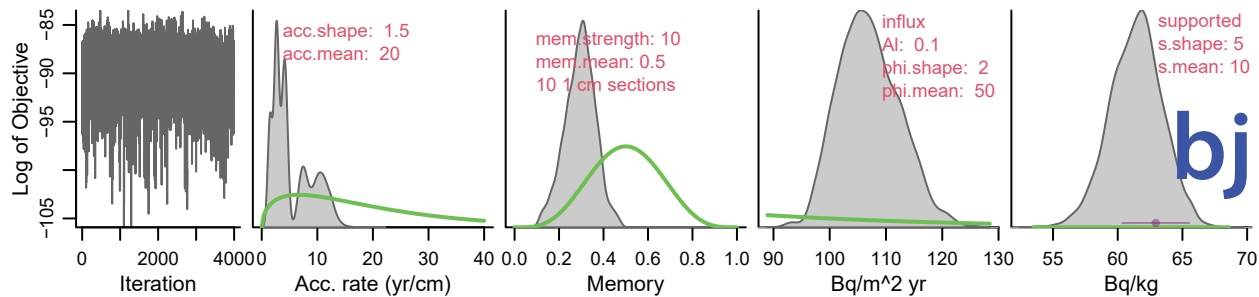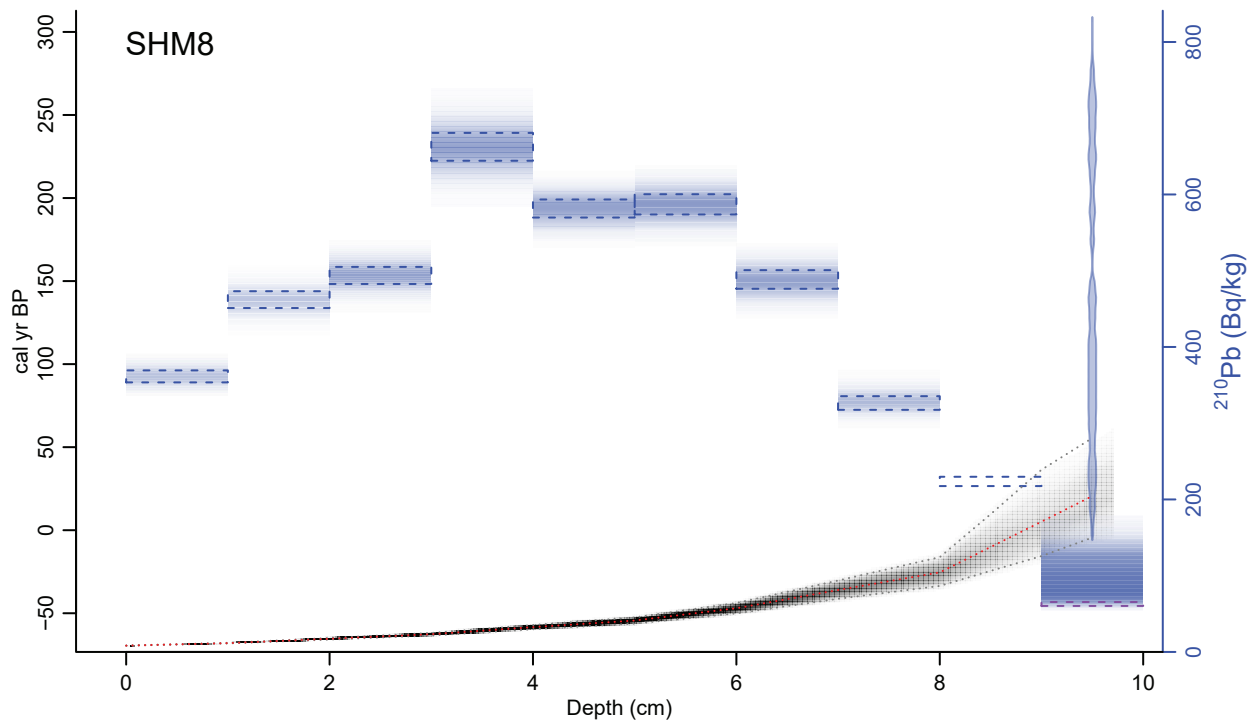

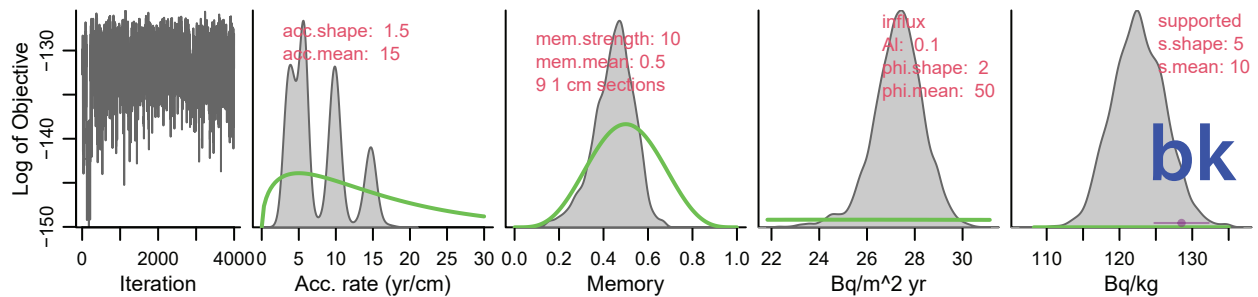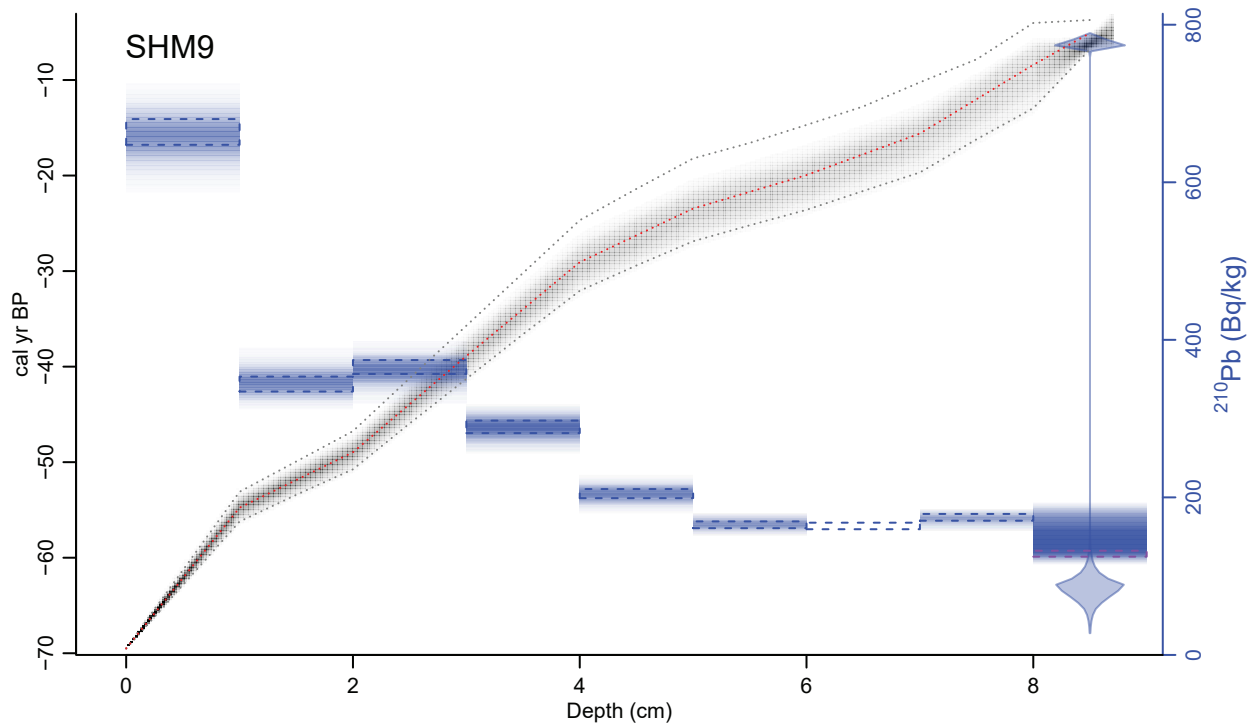

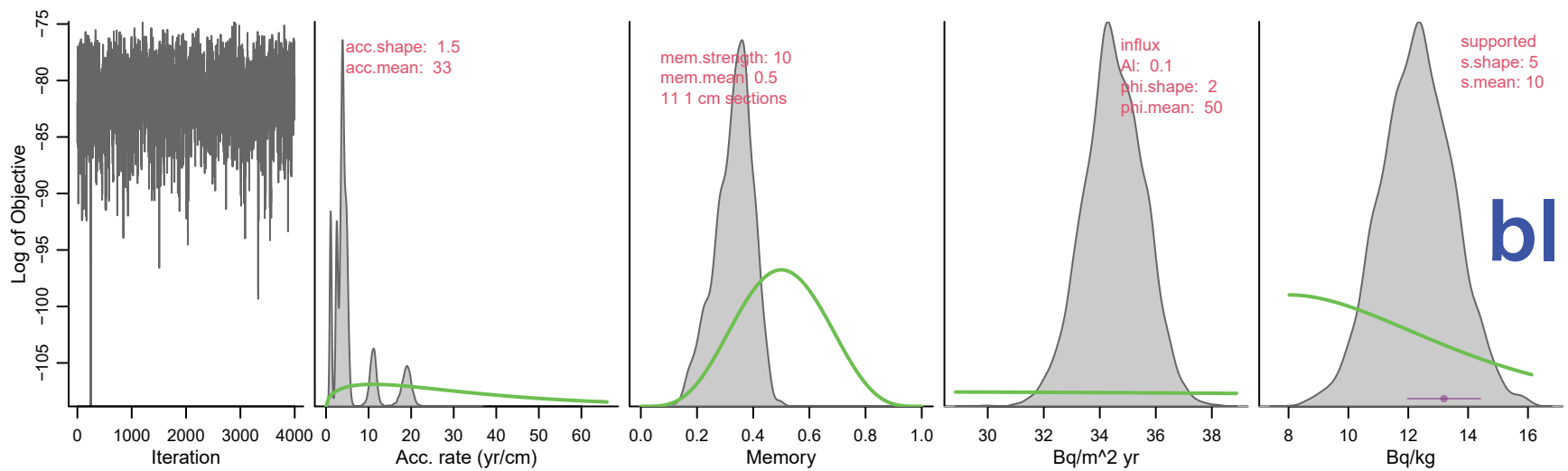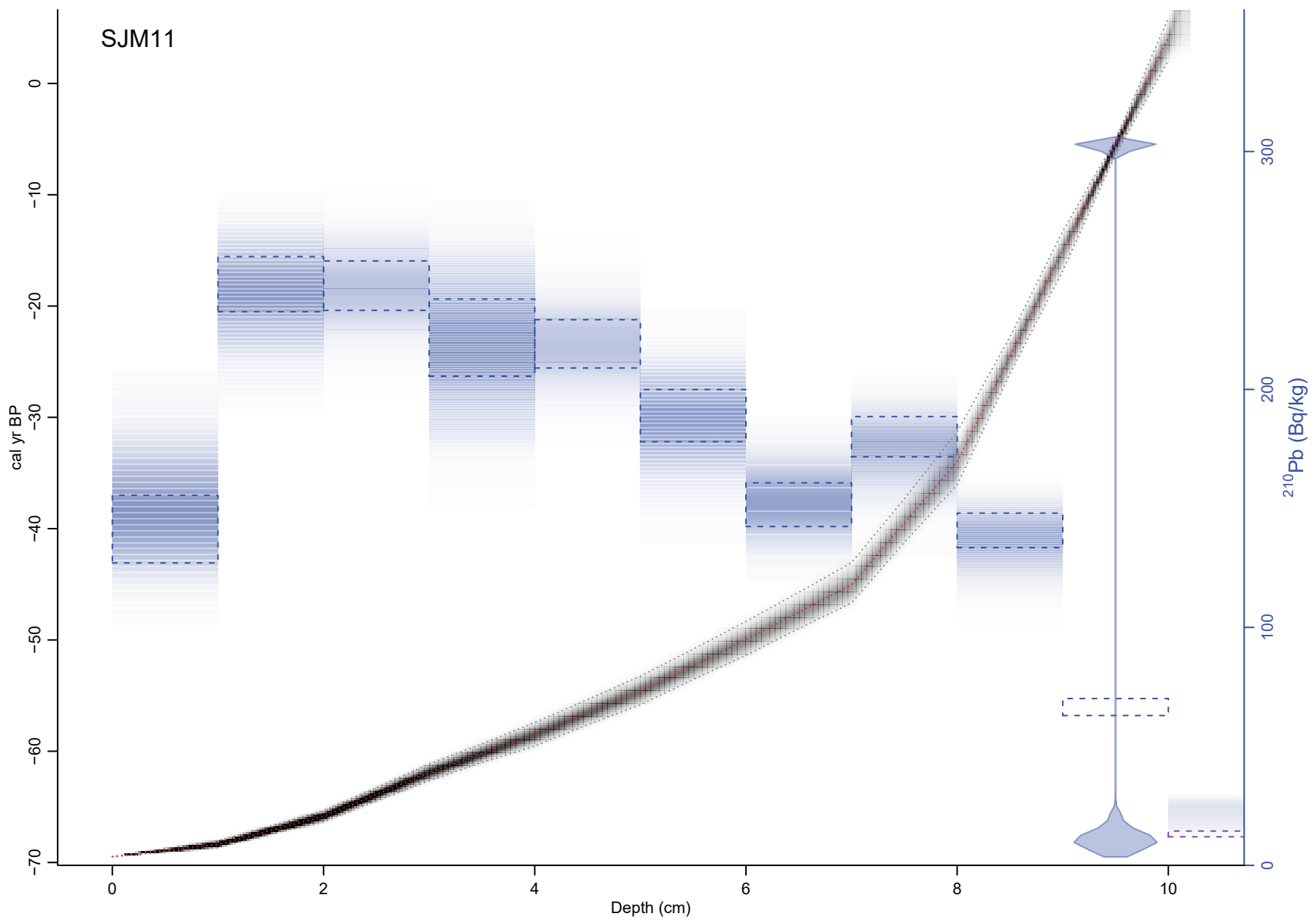

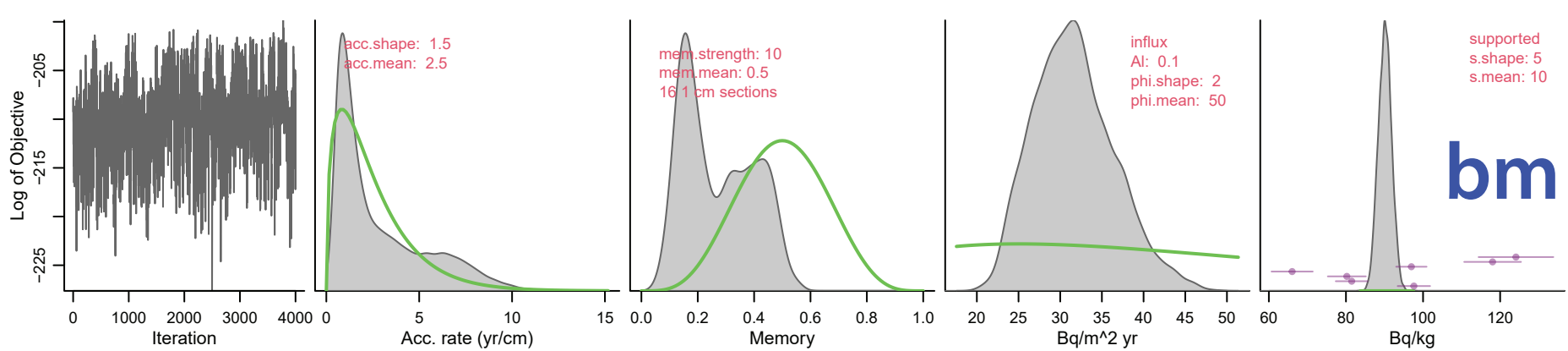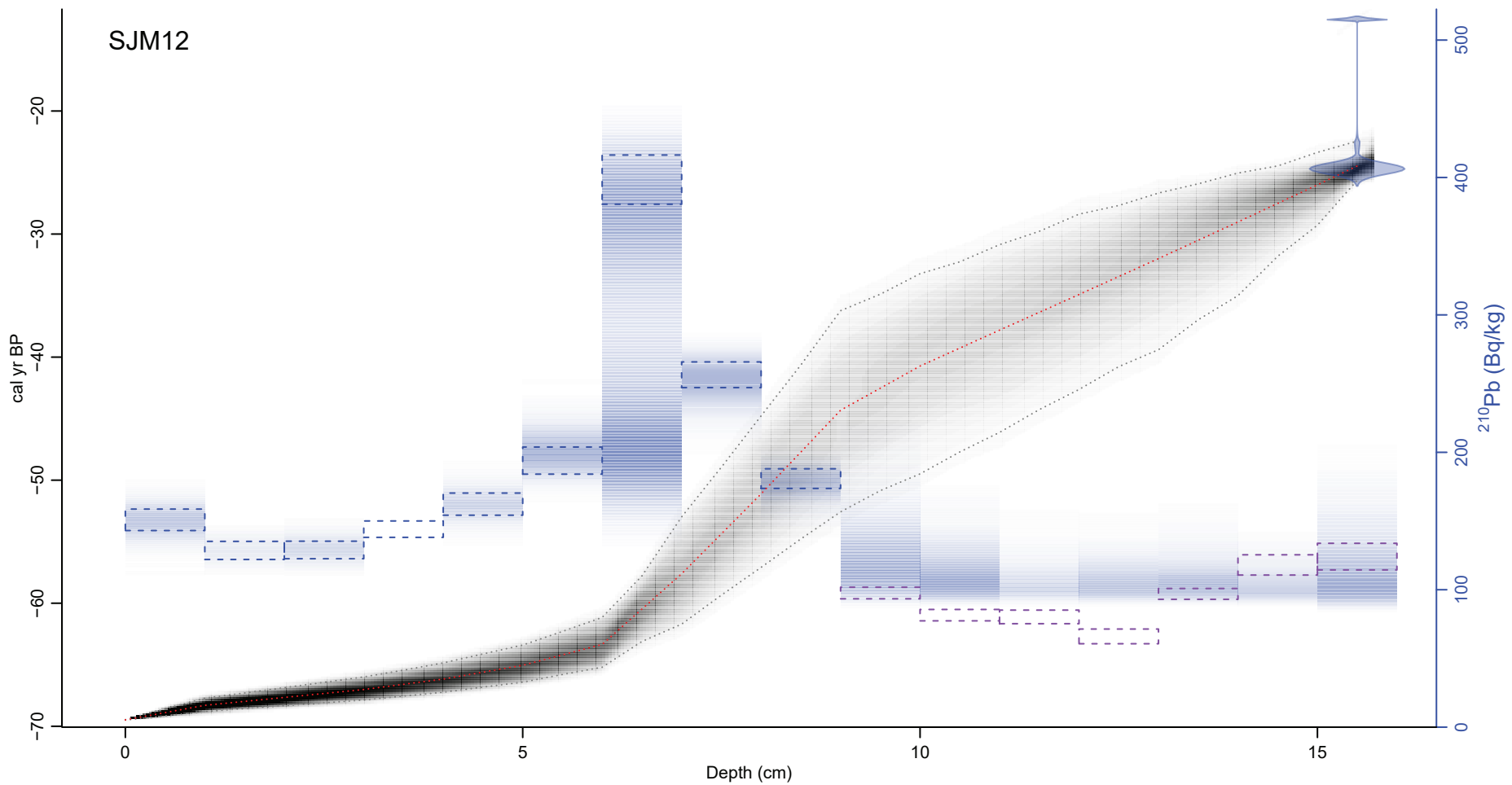

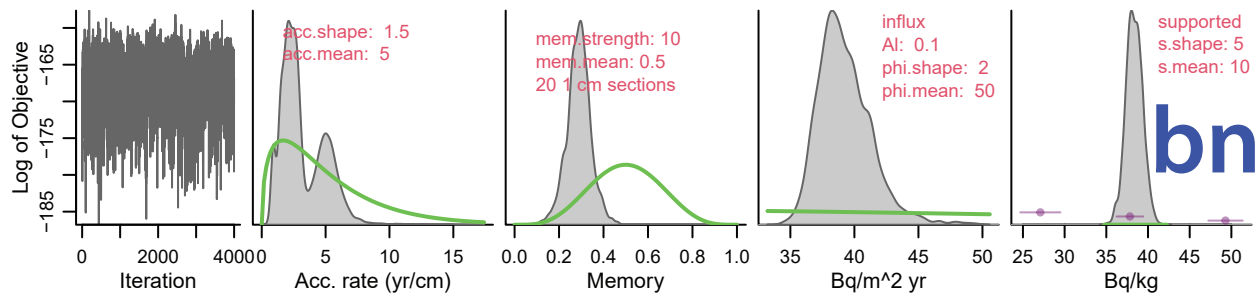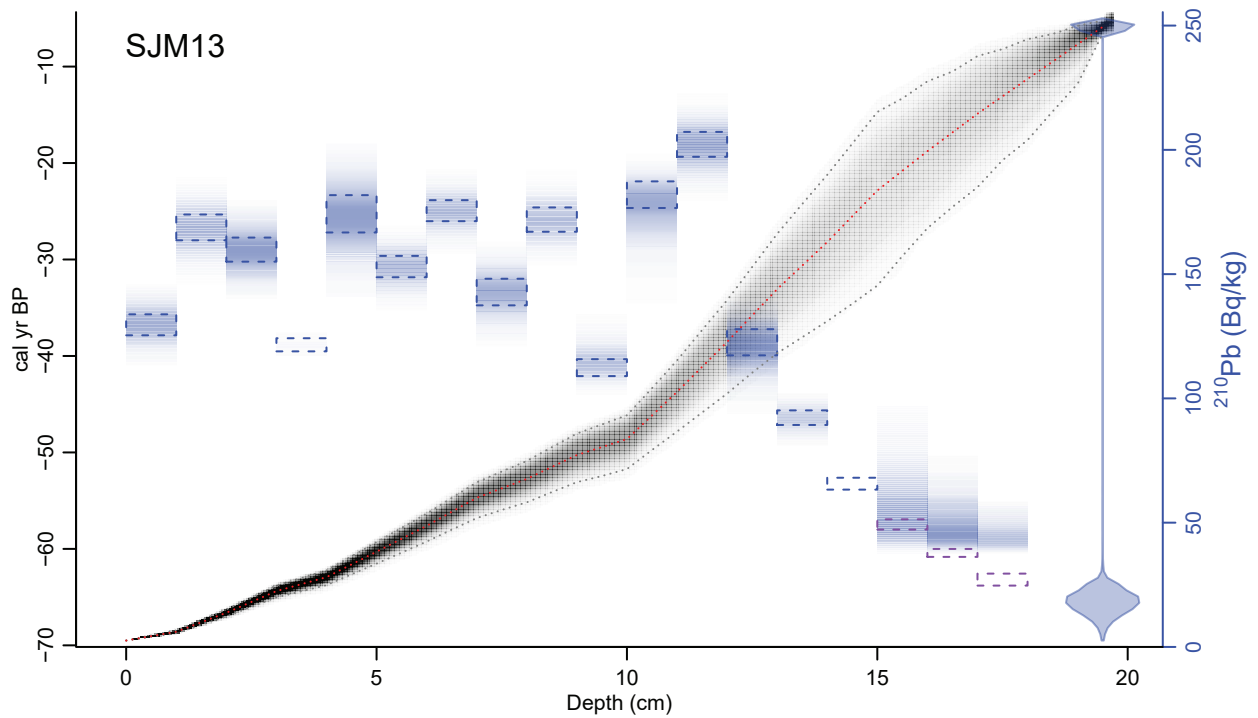

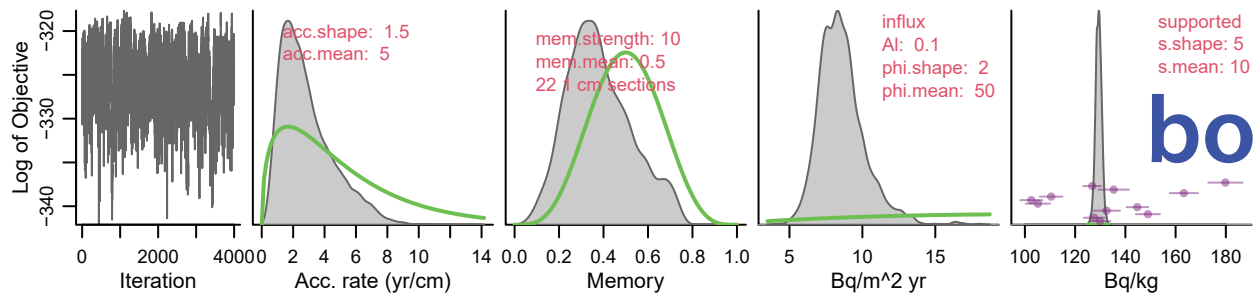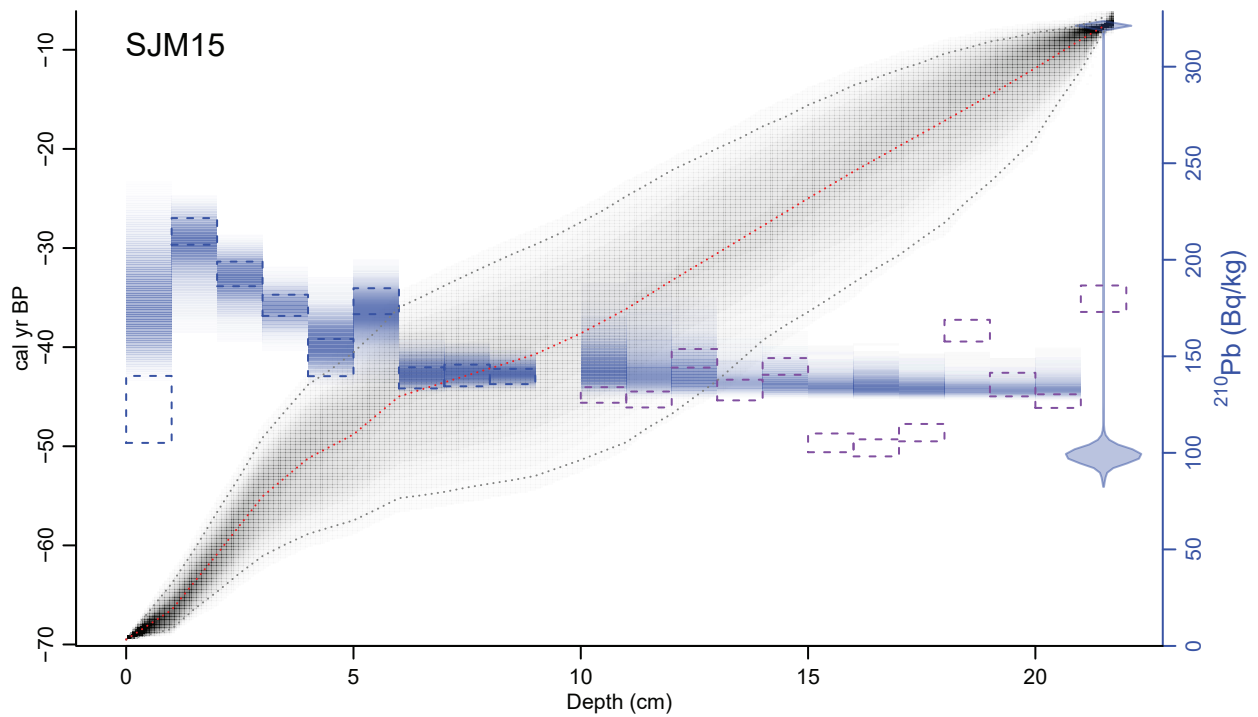

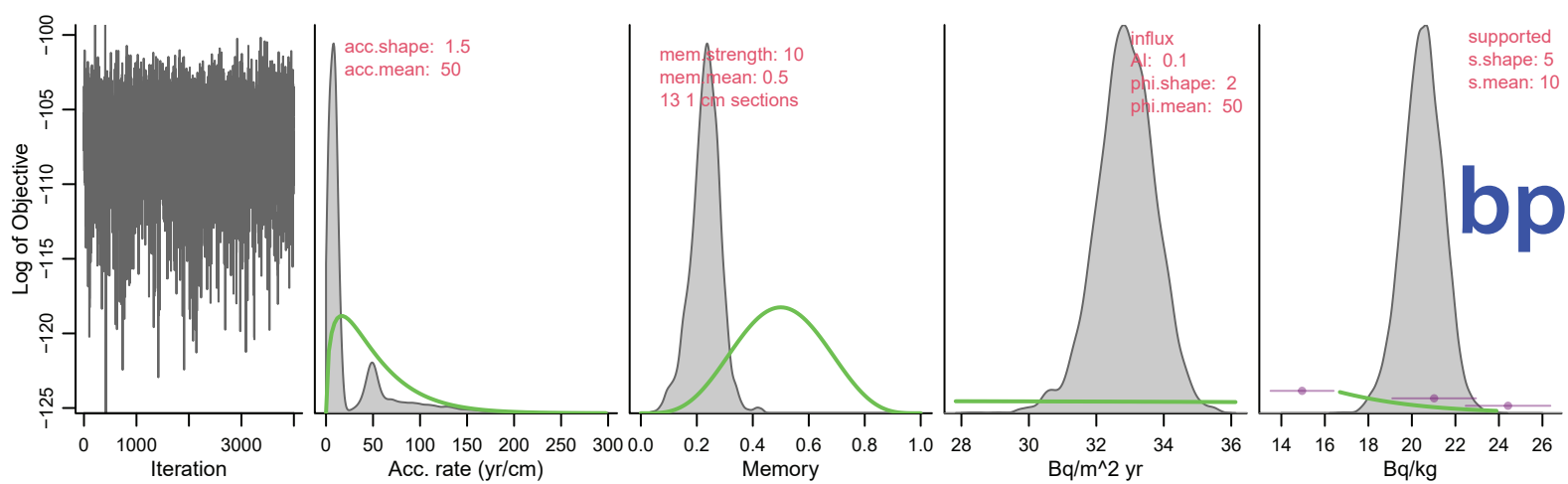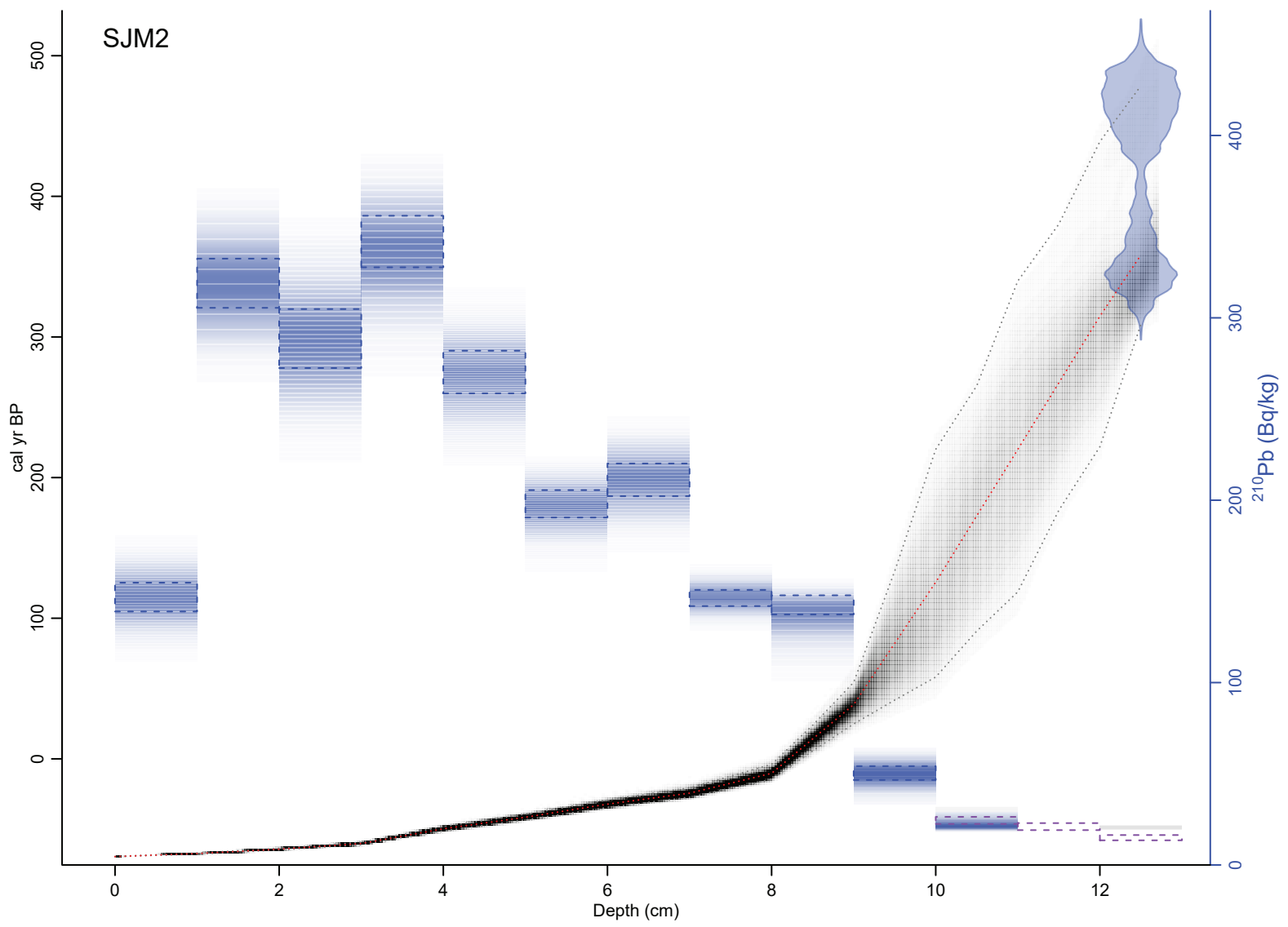

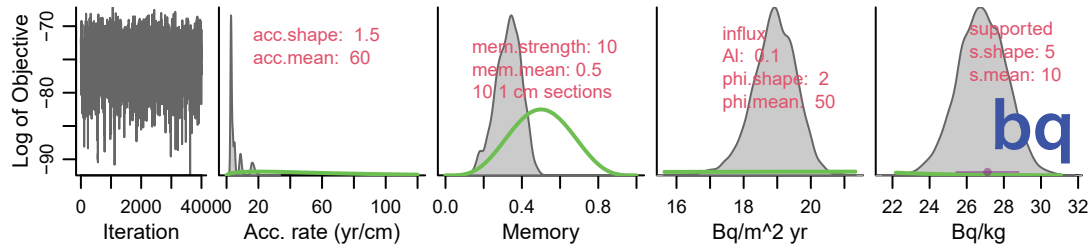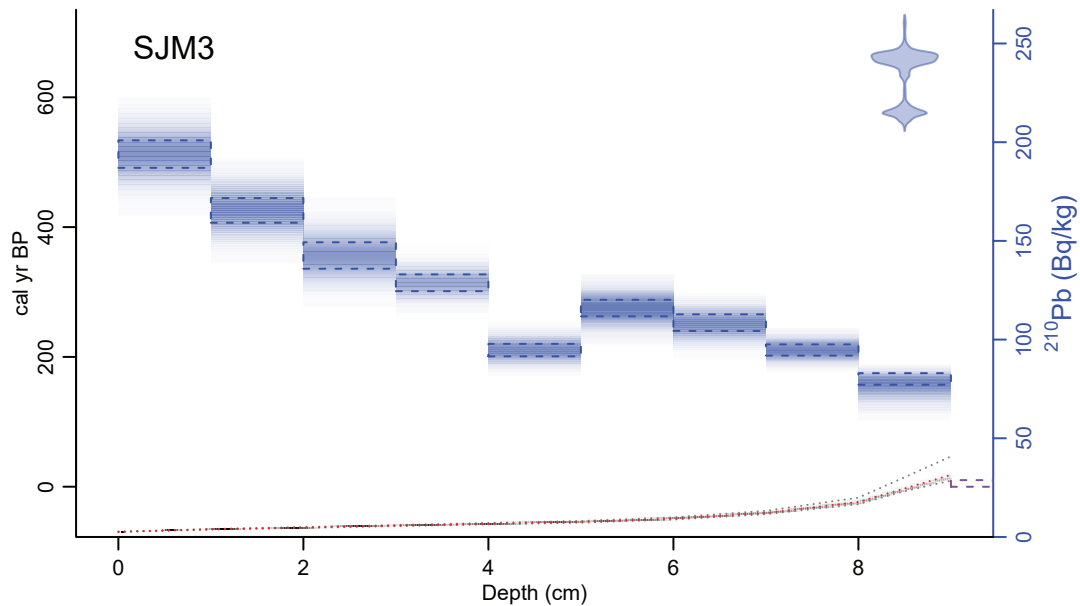

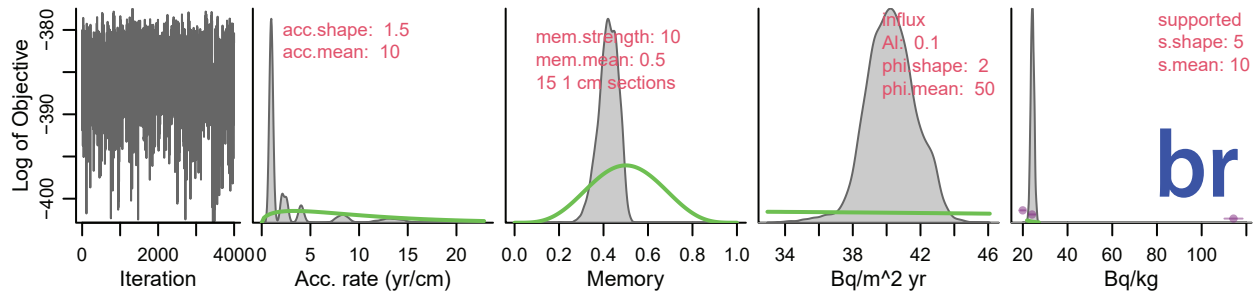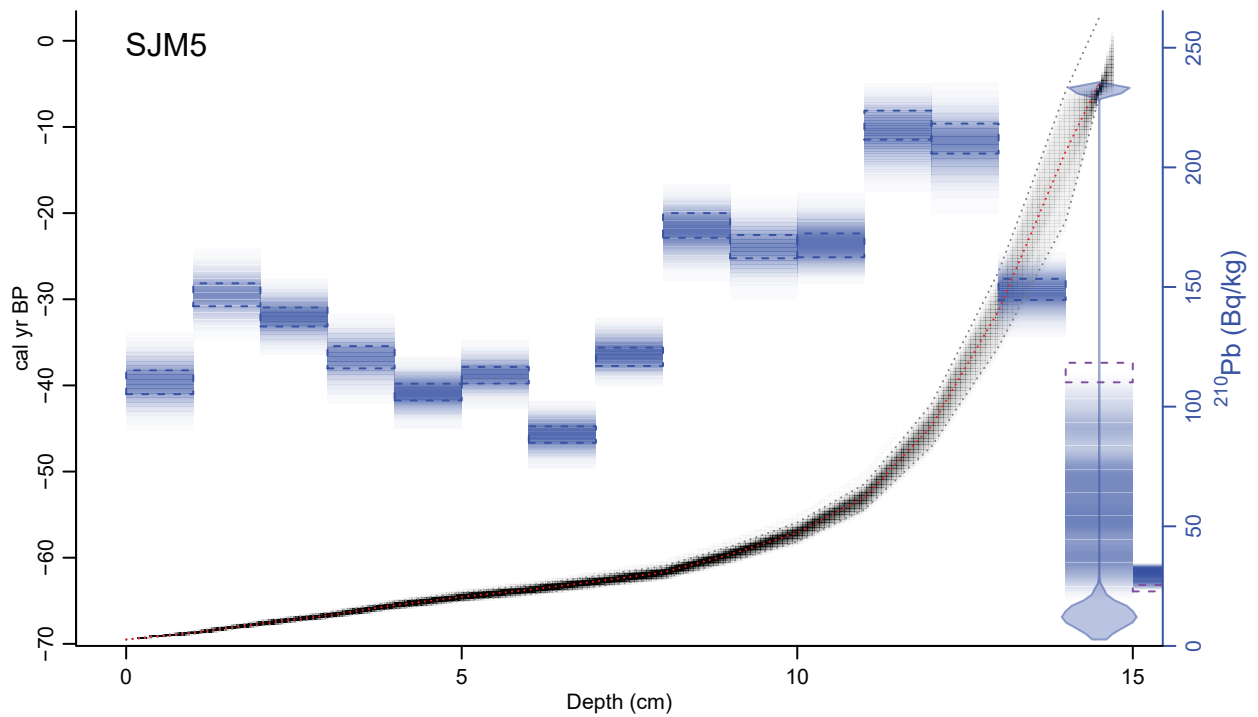

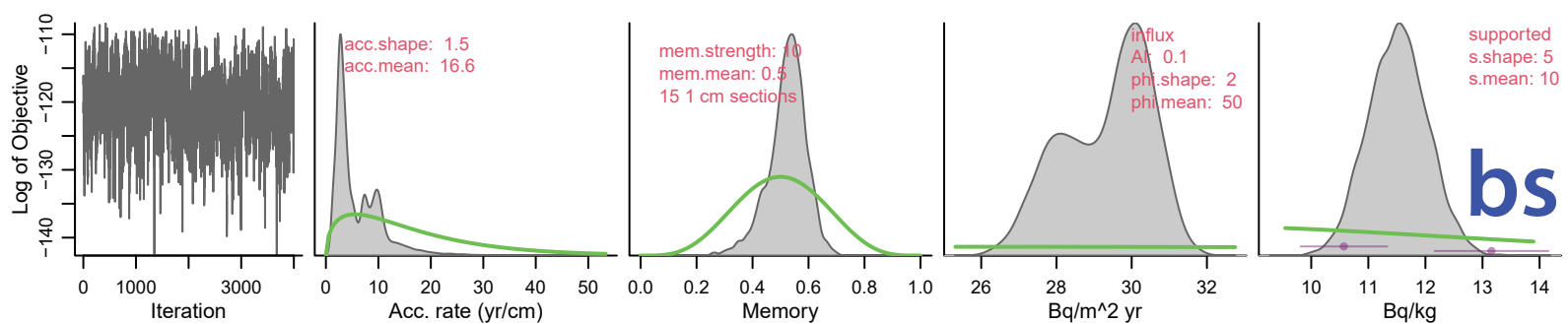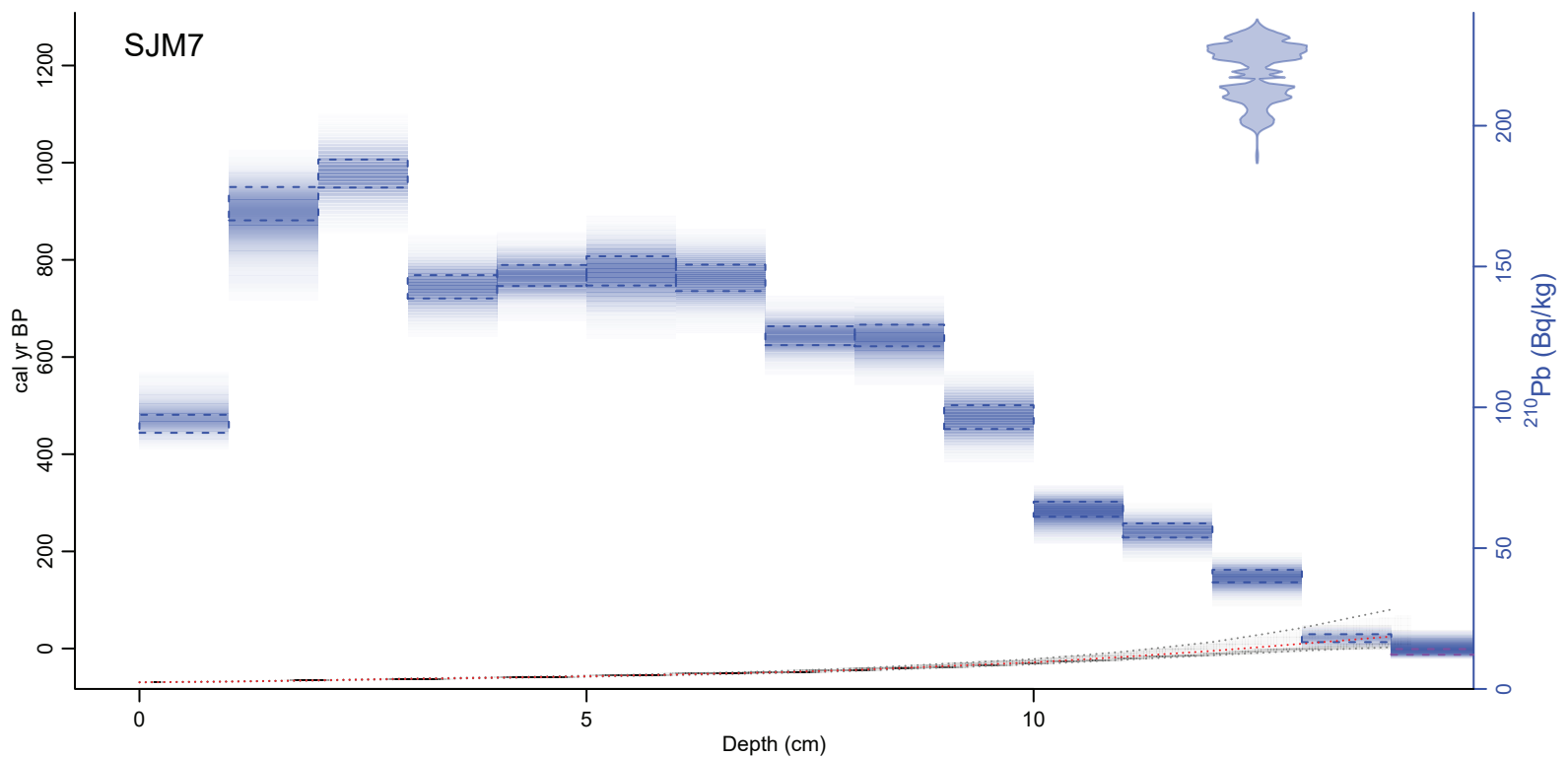

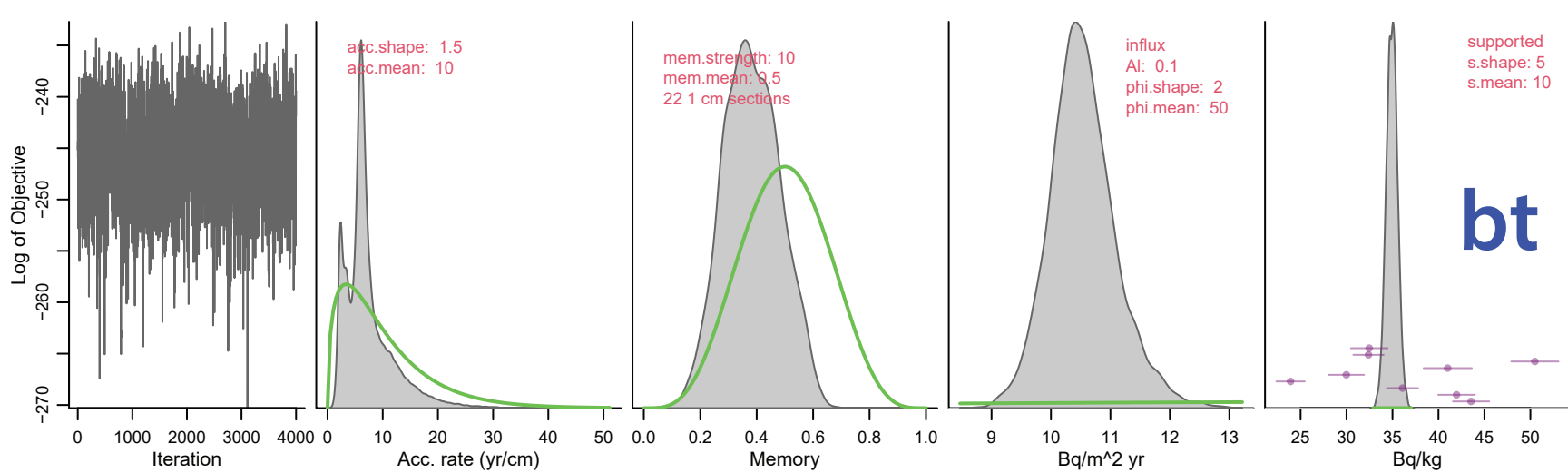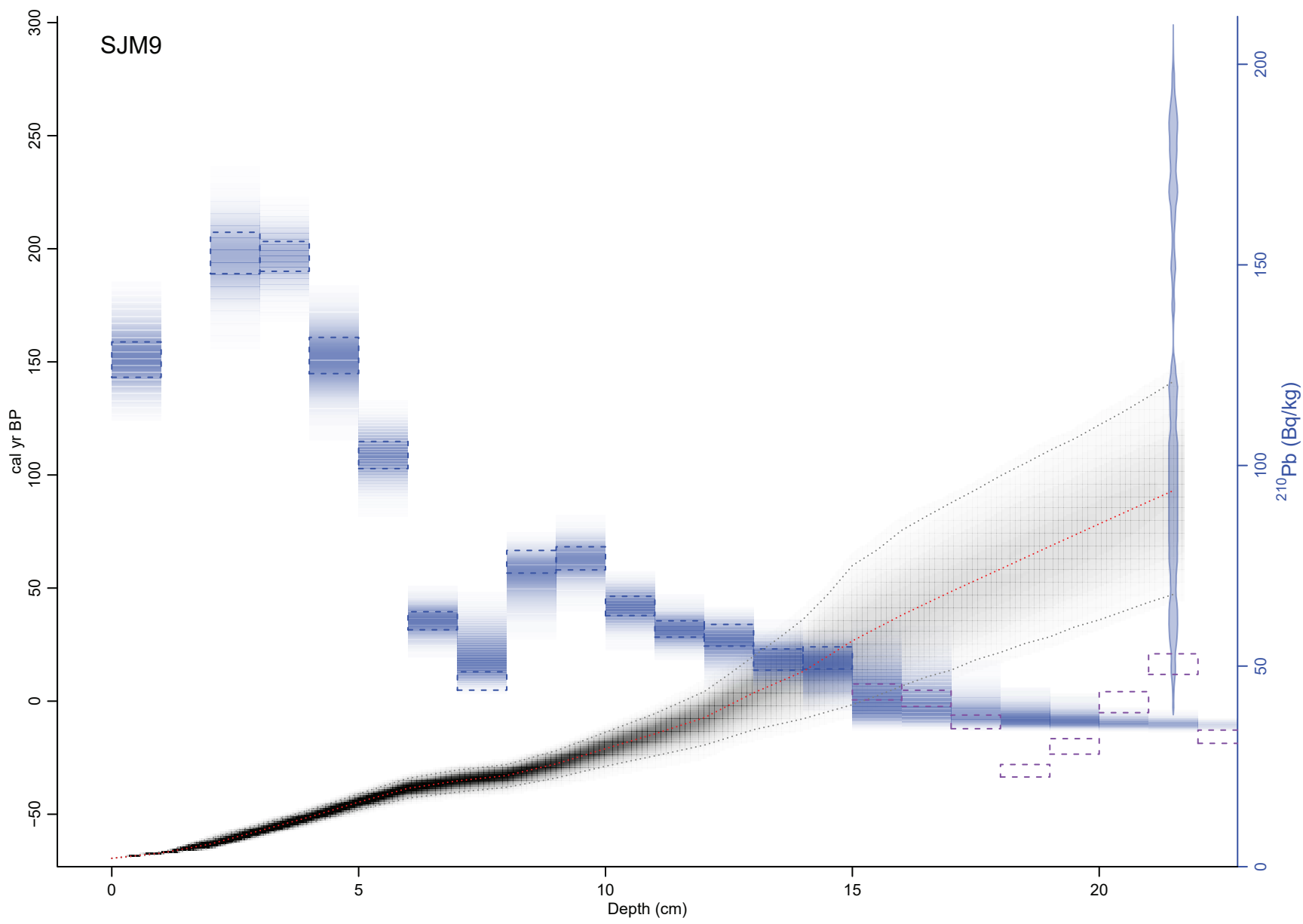

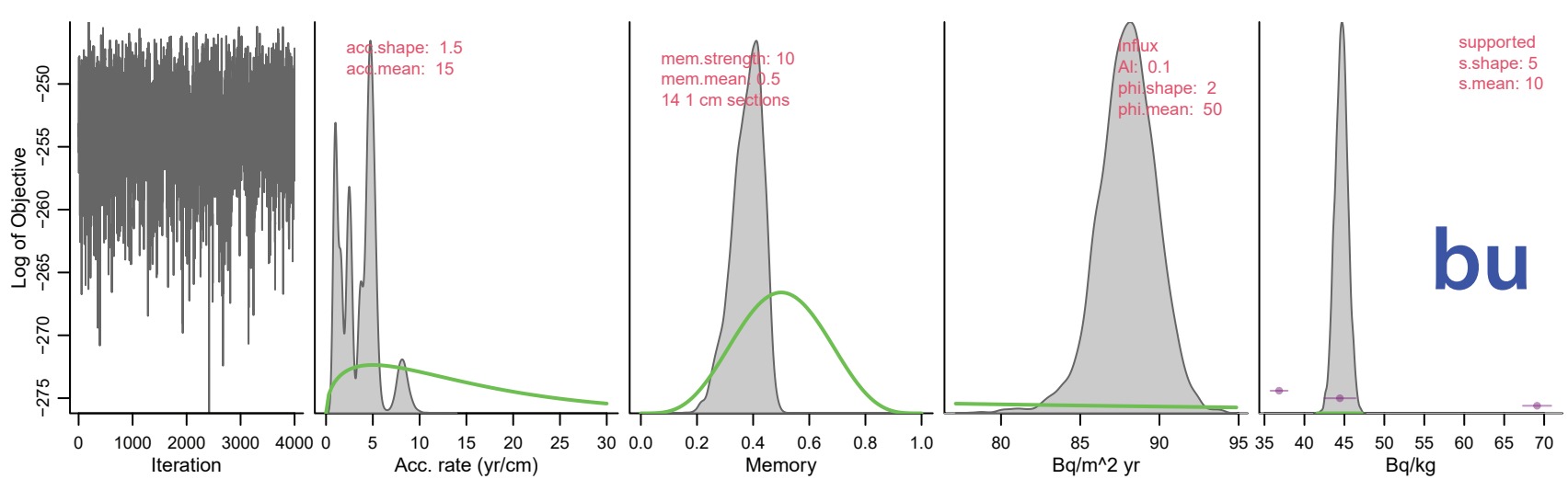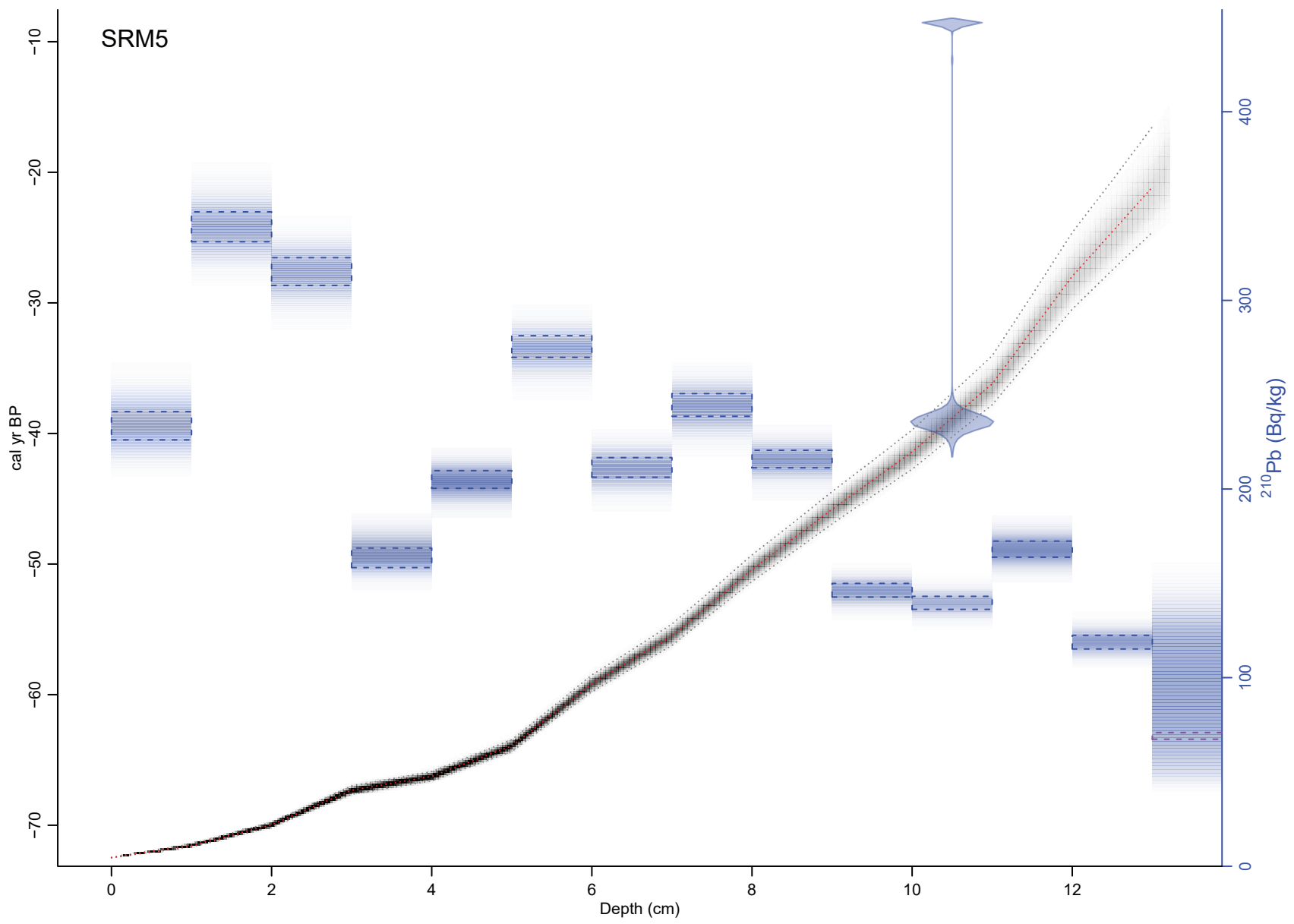

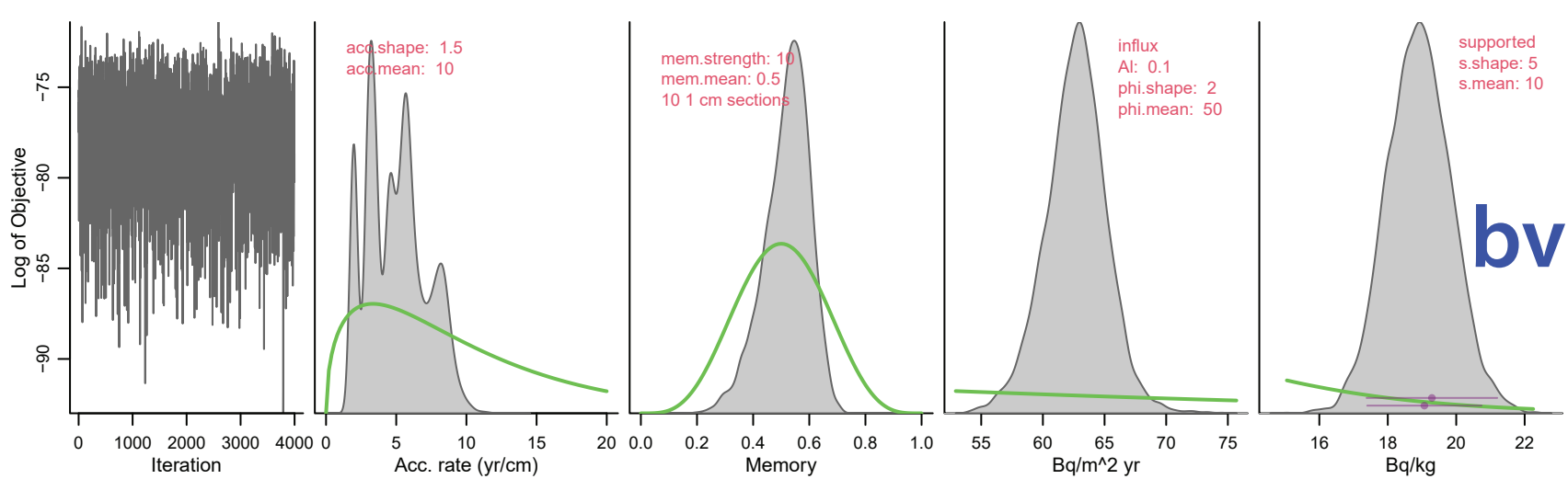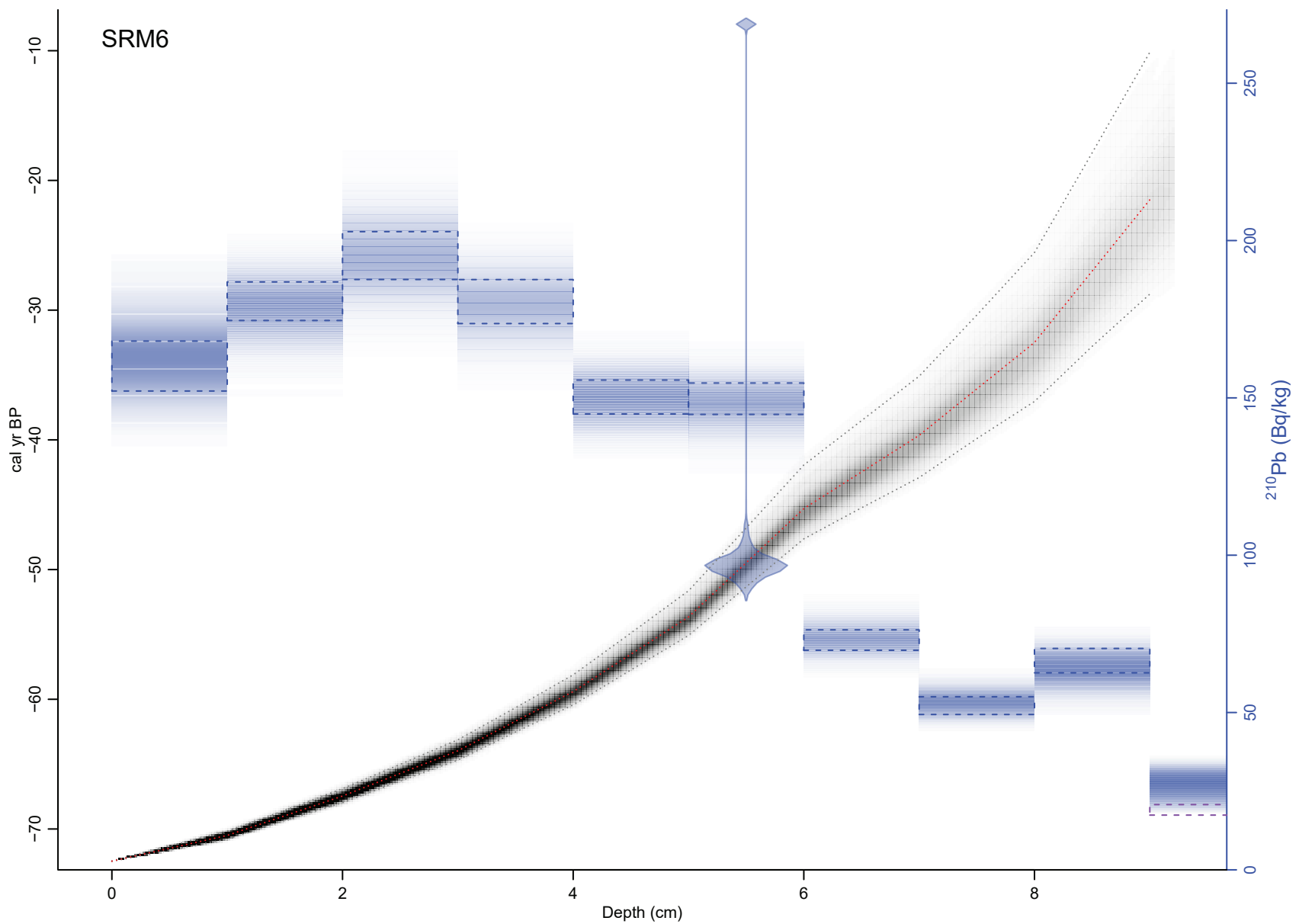

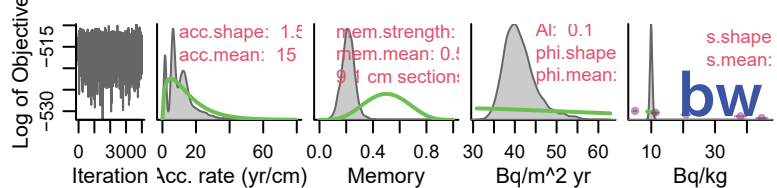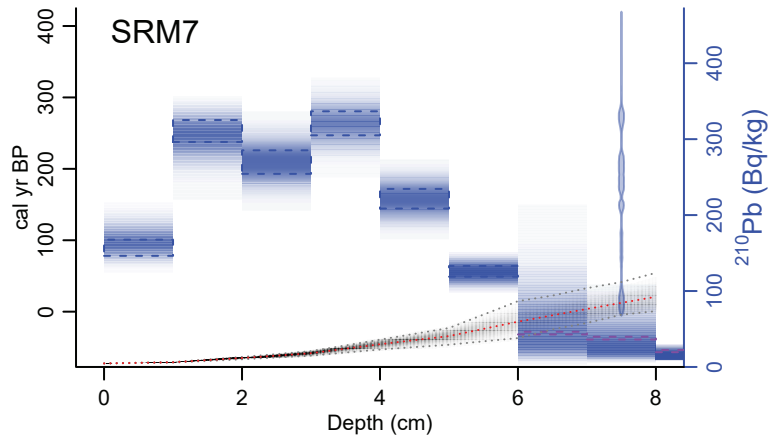

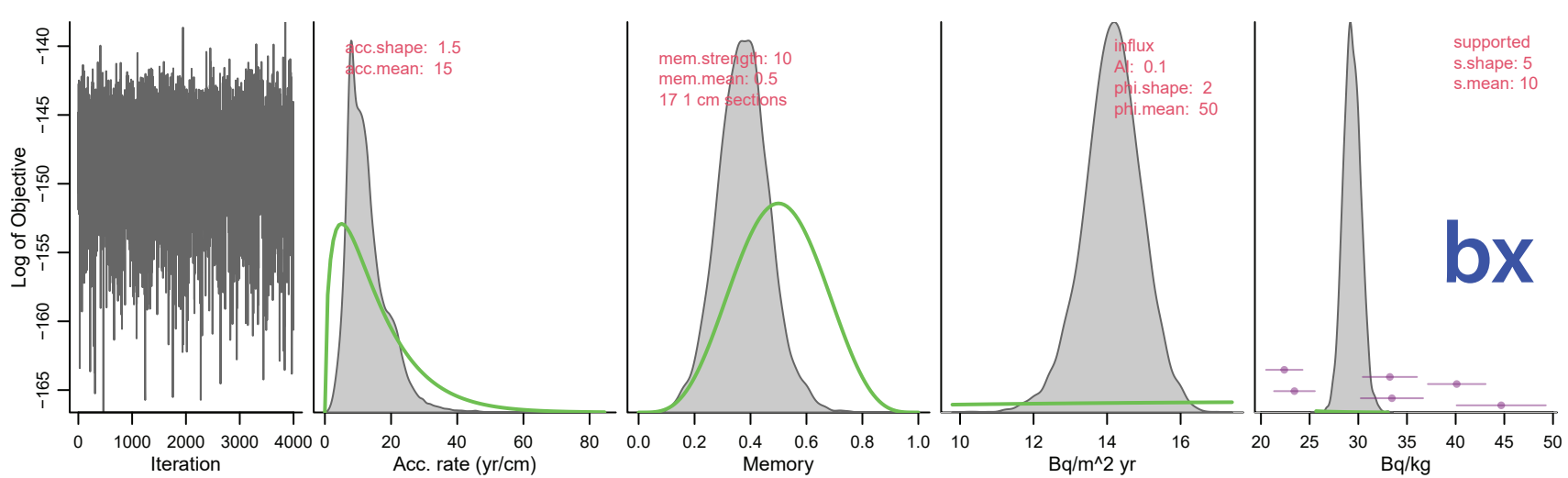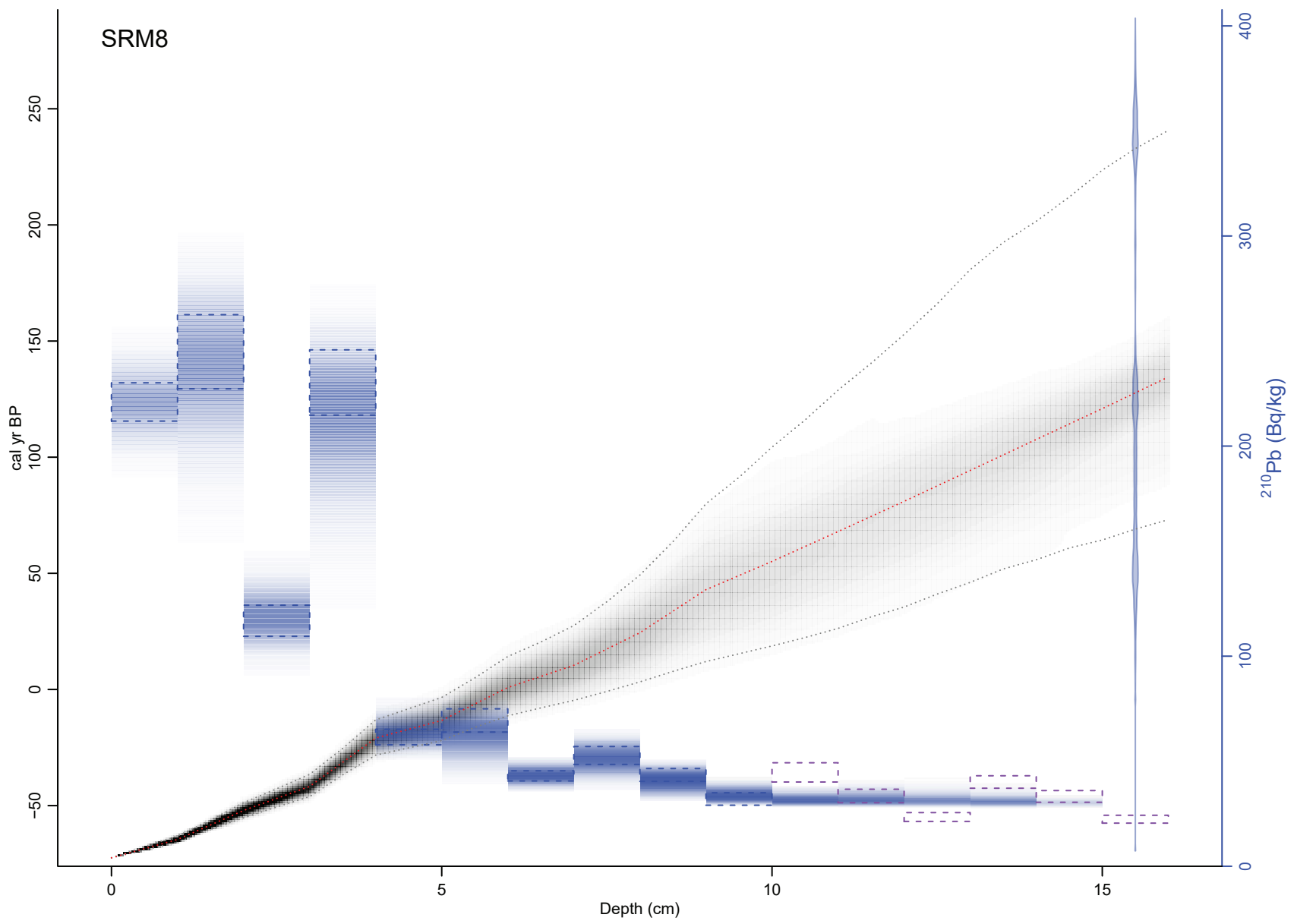

Supplement: Supplementary file 3 — Figure S1: gcb70684‐sup‐0003‐FigureS1.pdf. [file GCB-32-e70684-s002.pdf]
